# [Tc(NO)(Cp)(PPh_3_)Cl] and [Tc(NO)(Cp)(PPh_3_)(NCCH_3_)](PF_6_), and Their Reactions with Pyridine and Chalcogen Donors

**DOI:** 10.3390/molecules29051114

**Published:** 2024-03-01

**Authors:** Moritz Johannes Ernst, Abdullah Abdulkader, Adelheid Hagenbach, Guilhem Claude, Maximilian Roca Jungfer, Ulrich Abram

**Affiliations:** 1 Institute of Chemistry and Biochemistry, Freie Universität Berlin, Fabeckstr. 34/36, D-14195 Berlin, Germany; ernstm96@zedat.fu-berlin.de (M.J.E.); abdullah.abdulkader@admin.uni-giessen.de (A.A.); adelheid.hagenbach@uni-tuebingen.de (A.H.); guilhem.claude@charite.de (G.C.); 2 Ruprecht-Karls Universität Heidelberg, Im Neuenheimer Feld 271, D-69120 Heidelberg, Germany

**Keywords:** technetium, cyclopentadienyl compounds, nitrosyl complexes, ^99^Tc NMR, DFT

## Abstract

Reactions of the technetium(I) nitrosyl complex [Tc(NO)(Cp)(PPh_3_)Cl] with triphenylphosphine chalcogenides EPPh_3_ (E = O, S, Se), and Ag(PF_6_) in a CH_2_Cl_2_/MeOH mixture (*v*/*v*, 2/1) result in an exchange of the chlorido ligand and the formation of [Tc(NO)(Cp)(PPh_3_)(EPPh_3_)](PF_6_) compounds. The cationic acetonitrile complex [Tc(NO)(Cp)(PPh_3_)(NCCH_3_)]^+^ is formed when the reaction is conducted in NCCH_3_ without additional ligands. During the isolation of the corresponding PF_6_^−^ salt a gradual decomposition of the anion was detected in the solvent mixture applied. The yields and the purity of the product increase when the BF_4_^−^ salt is used instead. The acetonitrile ligand is bound remarkably strongly to technetium and exchange reactions readily proceed only with strong donors, such as pyridine or ligands with ‘soft’ donor atoms, such as the thioether thioxane. Substitutions on the cyclopentadienyl ring do not significantly influence the ligand exchange behavior of the starting materia**l**. ^99^Tc NMR spectroscopy is a valuable tool for the evaluation of reactions of the complexes of the present study. The extremely large chemical shift range of this method allows the ready detection of corresponding ligand exchange reactions. The observed ^99^Tc chemical shifts depend on the donor properties of the ligands. DFT calculations support the discussions about the experimental results and provide explanations for some of the unusual findings.

## 1. Introduction

**Scheme 1:**
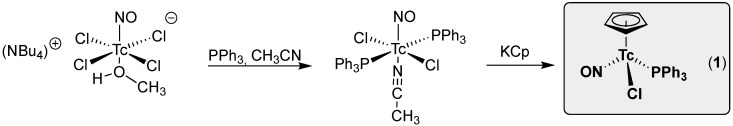
Synthesis of [Tc(NO)(Cp)(PPh_3_)Cl] [31,32,33].

The chemistry of the {Re(NO)(Cp)(PPh_3_)}^+^ fragment has been comprehensively studied by John Gladysz et al. and by some other groups, and the results have been published in more than a hundred papers [1,2,3,4,5,6,7,8,9,10,11,12,13,14,15,16,17,18,19,20,21,22,23,24,25,26,27,28,29,30]. The chlorido ligand of the chiral parent complex [Re(NO)(Cp)(PPh_3_)Cl] can readily be replaced by numerous inorganic and organic ligands. The analogous technetium(I) complex [Tc(NO)(Cp)(PPh_3_)Cl] (**1**) can be prepared in a simple two-step procedure starting from (NBu_4_)[Tc(NO)Cl_4_(MeOH)] [31,32,33] (Scheme 1).

Compound **1** is a suitable starting material for the synthesis of other complexes having the {Tc(NO)(Cp)(PPh_3_)}^+^ core, and a few neutral and cationic complexes have been synthesized with monodentate ligands, such as Br^−^, CO, and phenyl [33], or I^−^, I_3_^−^, SCN^−^, CF_3_COO^−^, and CF_3_SO_3_^−^ [34]. But derivatives with two phosphine ligands, dimeric products, and carbenes are also accessible [35].

Ligand exchange reactions of **1** with neutral chalcogen–donor ligands have so far not been reported and corresponding reactions with the analogous rhenium complex [Re(NO)(Cp)(PPh_3_)Cl] are also rare, where only a few products with ketones [36,37,38,39], lactones [40,41], disulfides [42,43], S-bonded dimethyl disulfide [42], and a side-on bonded thioketone [44] have been published. In the present study, we report reactions of [Tc(NO)(Cp)(PPh_3_)Cl] (**1**) with triphenylphosphine chalcogenides and acetonitrile. With the latter reaction, a ready approach to the cationic ‘solvent complex’ [Tc(NO)(Cp)(PPh_3_)(NCCH_3_)]^+^ was sought, which might have potential as a precursor for ongoing ligand exchange reactions.

Structural and reactivity studies with the long-lived technetium isotope ^99^Tc (weak β^–^-emitter, half-life: approximately 2 × 10^5^ years) are important to understand general trends in the Periodic Table of elements. There is persuasive evidence that, particularly in their medium and high oxidation states, the coordination chemistry of the homologous elements technetium and rhenium shows remarkable differences. This is mainly attributed to variations in their redox potentials [45,46]. More recently, such differences have also become evident for low-valent technetium and rhenium complexes and for corresponding organometallic compounds [47,48,49,50]. In addition to such basic considerations, novel technetium compounds are always interesting as potential tracers for diagnostic nuclear medicine. The short-lived, meta-stable nuclide ^99m^Tc (pure γ-emitter, half-life 6 h) is the workhorse in diagnostic nuclear medicine, with approximately 40 million annual administrations worldwide [51,52,53,54,55,56,57]. Due to the low concentrations of ^99m^Tc solutions (commercial ^99^Mo/^99m^Tc generator systems deliver this nuclide at an approximate nanomolar concentration level), chemical approaches towards the development of potentially novel technetium-based radiopharmaceuticals are frequently pursued by means of the long-lived isotope. Thus, in the present article, some experiments with substituted cyclopentadienyl ligands are also described, which may lead to compounds for potential bioconjugation.

## 2. Results and Discussion

### 2.1. [Tc(NO)(Cp)(PPh_3_)(EPPh_3_)]^+^ (E = O, S, Se) Complexes

The replacement of the chlorido ligand of compound **1** by triphenylphosphine chalcogenides proceeds only slowly and remains incomplete when no chloride scavenger is present. The addition of one equivalent of Ag(PF_6_), however, activates compound **1** and results in an immediate precipitation of AgCl as a light grey powder. Supposably, an unstable solvent complex is formed intermediately. Similar compounds are known for rhenium, where such compounds with dichloromethane and alkoxides have been reported [37,58]. ^99^Tc NMR spectra taken from the reaction mixture after the precipitation of AgCl show a new resonance at −211 ppm ([Tc(NO)(Cp)(PPh_3_)Cl] (**1**): −231 ppm), hinting at the potential formation of a related reactive intermediate with a loosely bonded ClCH_2_Cl ligand or the formation of [{Tc(NO)(Cp)(PPh_3_)}_2_Cl]^+^ (−220 ppm) [35]. The chemical shift difference between the three signals is only small with regard to the large scale of ^99^Tc NMR shifts [59,60,61,62,63,64], which can be attributed to the essentially unchanged first coordination sphere of technetium or a potential dynamic equilibrium between labile solvent complexes, [Tc(NO)(Cp)(PPh_3_)Cl], and the dimeric cation.

**Scheme 2:**
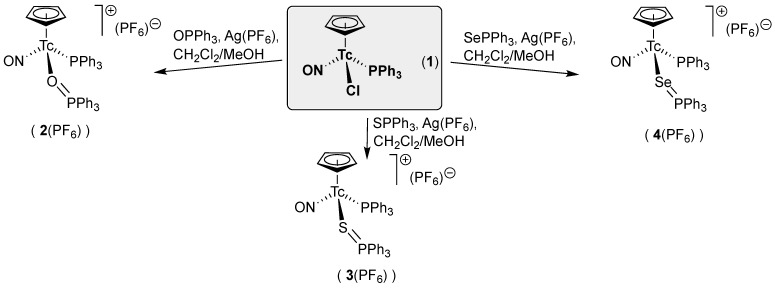
Synthesis of the [Tc(NO)(Cp)(PPh_3_)(EPPh_3_)](PF_6_) (E = O, S, Se) complexes.

An exchange of the chlorido ligand of **1** in favor of oxygen donor ligands, such as OSO_2_CF_3_^−^ or OOCCF_3_^−^, results in chemical shift changes of several hundred ppm [34]. After addition of the triphenylphosphine oxide, sulfide, or selenide, the color of such solutions turned to orange-red upon heating under reflux, and crystalline products could be isolated after filtration, removal of all volatiles, and recrystallization from CH_2_Cl_2_/diethyl ether solutions (Scheme 2). 

The products are air-stable complexes, which are readily soluble in solvents such as CH_2_Cl_2_ or THF. The ν_NO_ IR stretches appear between 1686 and 1705 cm^−1^, which is in the common range for Tc(I) compounds and reflect a considerable degree of back-donation from the electron-rich d^6^ metal ions [65]. Clear differences between the three complexes are observed in their ^31^P and ^99^Tc NMR spectra. While no significant changes for the positions of the ^31^P{^1^H} signals of the triphenylphosphine ligands are visible (they are all extremely broad and are found between 30 and 50 ppm), the chemical shifts obtained for the phosphine chalcogenide ligands appear at 57.8 (oxide), 51.2 (sulfide), and 30.0 ppm (selenide). Similarly, the experimental ^99^Tc NMR chemical shift obtained for the cation with triphenylphosphine oxide (**2**) is found at +254 ppm, whereas those for the corresponding sulfide (**3**) and selenide (**4**) complexes are significantly shifted up-field (−781 and −891 ppm). These experimental findings suggest noticeable differences in the electronic situation in the structurally related [Tc(NO)(Cp)(PPh_3_)(EPPh_3_)](PF_6_) (E = O, S, Se) complexes and shall be a matter of more detailed considerations of the bonding situation inside the compounds by means of computational methods (vide infra).

**Figure 1:**
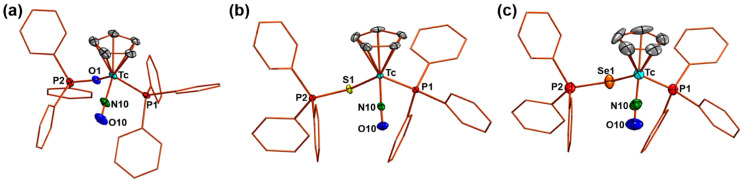
Representations of (**a**) [Tc(NO)(Cp)(PPh_3_)(OPPh_3_)]^+^ (**2**), (**b**) [Tc(NO)(Cp)(PPh_3_)(SPPh_3_)]^+^ (**3**), and (**c**) [Tc(NO)(Cp)(PPh_3_)(SePPh_3_)]^+^ (**4**). Thermal ellipsoids represent 50 percent probability.

Some of the spectroscopic differences are also reflected by the bond lengths and angles determined for the solid-state structures of the complexes. Single crystals suitable for X-ray diffraction were obtained from CH_2_Cl_2_/diethyl ether mixtures. Figure 1 depicts structural plots of the complex cations. Selected bond lengths and angles are summarized in Table 1. 

Compounds **2**–**4** represent the first triple of structurally characterized triphenylphosphine chalcogenide complexes of the ‘group 7’ elements comprising oxides, sulfides, and selenides at an identical basic structure. Thus, they may allow some conclusions about the influence of the chalcogenide donor atoms on the coordination situation. A first remarkable structural feature is provided by the differences in the Tc-E-P angles, which are close to the tetrahedral angle in the sulfide and selenide complexes, while the Tc-O-P angle of 133.3(2)° in compound **4** is significantly widened. This is in line with the bonding situation in the few other structurally characterized phosphine oxide complexes of technetium, where Tc-O-P angles between 138.5 and 155.3° were observed [33,60,62,66,67]. Similar results are observed for the corresponding triphenylphosphine oxide complexes of rhenium, for which 91 entries were found in the CCDC database with clearly widened Re-O-P angles (131.7–172.5°) [11]. Corresponding (non-restrained) phosphine sulfide or selenide complexes of technetium are not available for comparison. But in the hitherto only two studied triphenylphosphine sulfide complexes of rhenium, [Re(CO)_4_(µ-H)(µ-P(cyclohexyl)_2_}-Re(CO)_3_(SPPh_3_)] and [Re(CO)_4_(µ-H)(µ-S-naphtalene)(Re(CO)_3_(SPPh_3_)], relatively small Re-S-P angles of 121.5 and 112.8° were observed [68,69]. 

The structural features agree with the ^99^Tc NMR chemical shifts reported above, which show a closer similarity between the electronic situation in the phosphine sulfide and selenide complexes (**3** and **4**) compared with the triphenylphosphine oxide complex **2**. Therefore, the structurally related [Tc(NO)(Cp)(PPh_3_)(EPPh_3_)](PF_6_) (E = O, S, Se) complexes were studied by computational means on the B3LYP/Stuttgart 1997(Tc)/LANL2DZ(N,P,S,Se)/6-311 + G**(remaining atoms) level. The fuzzy bond order of the P-E bond decreases in the order O > Se > S, while the corresponding Tc-E bond order increases in the same order. Compared to the free phosphine chalcogenides, the P-S bond order is reduced most drastically upon coordination from 1.60 to 1.07 (∆ = 0.53). The P-O bond order, which is reduced from 1.90 to 1.48 (∆ = 0.42), and the P-Se bond order, which changes from 1.53 to 1.12 (∆ = 0.40), are less affected by coordination. To further understand which electronic implications the coordination has, we calculated the corresponding atomic dipole-corrected Hirshfeld atomic charges (ADCH). The charge at the coordinated chalcogen atom increases linearly along the group from oxygen to selenium, which is similar to the trend in the free phosphine chalcogenides. Interestingly, a similar trend is seen for the charge at the phosphorus atom of the triphenylphosphine ligand that coordinates to technetium. The respective phosphorus atom is most positive in the case of the selenium co-donor and the least positive in the presence of the oxygen co-donor atom. Simultaneously, the charge at the two atoms bonded directly to the chalcogen atom, the phosphine chalcogenides phosphorus atom and the technetium atom, are very similar for selenium and sulfur, while the technetium atom in the oxygen-donor compound is considerably less negatively polarized and the phosphorus atom is much more positively polarized in comparison. These results neatly correspond to the shifts observed for the ^31^P{^1^H} and ^99^Tc NMR spectra of the compounds, where the (E=)P and Tc atoms are deshielded in the case of oxygen and more shielded in the case of S or Se. Exemplarily, a nearly linear correlation between the charge at the chalcogen-bonded phosphorus atom as well as the charge of the technetium atom and the ^99^Tc chemical shift is found, while the situation for the ^31^P{^1^H} shifts is more complex, emphasizing the mutual influence of many variables on the total electronic structure at the metal. A graphical representation is given in the Supplementary Materials. Similarly, and although the absolute values could not be reproduced, theoretical gas-phase GIAO-based isotropic NMR tensor calculations on the B3BP86/x2c-TZVPPall-s level show a linear correlation (R² ≈ 0.9988, see Supplementary Materials) with the experimentally observed chemical shifts for the series of structurally closely related compounds **1**–**4**. Hence, the relative trends between these compounds should be reliably represented by the applied theoretical methods.

**Figure 2:**
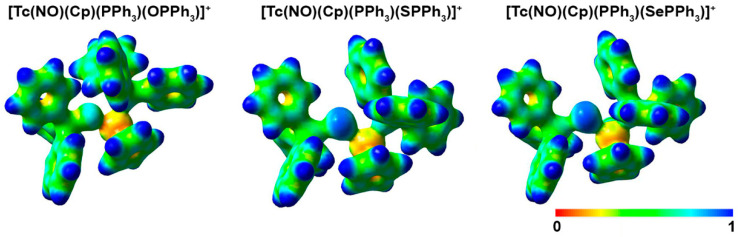
Mapping of the dimensionless electron localization function (ELF), where a perfectly localized pair of electrons is described by 1 (blue), while a lack of electron pairs (or empty orbital lobe) is described by 0 (red).

The difference between the charge at the E=PPh_3_ phosphorus atom upon coordination of the respective chalcogen atom to technetium increases in the order S < O < Se (Figure 2). The average influence of the identity of the chalcogen atom and the coordination to technetium can be readily understood by studying the electron localization function (ELF) at the chalcogen atoms. The corresponding 3D representations clearly visualize the influence of the chalcogen atoms identity on the electron-pair distribution within the Tc-E-P unit in accordance with the electronic charges and bond order considerations: less charge accumulation gives more neutral colors, while charge accumulation gives darker colors; thicker bond regions emphasizes higher absolute bond order; evenly distributed electron density gives large patches of similar color, while polarized regions (e.g., those with located lone-pairs) give smaller patches of deeper colors (Figure 2).

The nitrosyl ligands are essentially linearly bonded in all three compounds. Thus, they can be understood formally as NO^+^ units. This is in perfect agreement with all other hitherto structurally characterized nitrosyl compounds of technetium [32,33,34,35,66,70,71,72,73,74,75,76,77,78,79,80,81,82,83,84,85,86] and the short N10-O10 bonds reflect the π-backdonation discussed above. 

### 2.2. Synthesis and Reactions of [Tc(NO)(Cp)(PPh_3_)(NCCH_3_)]^+^ Salts

**Scheme 3:**
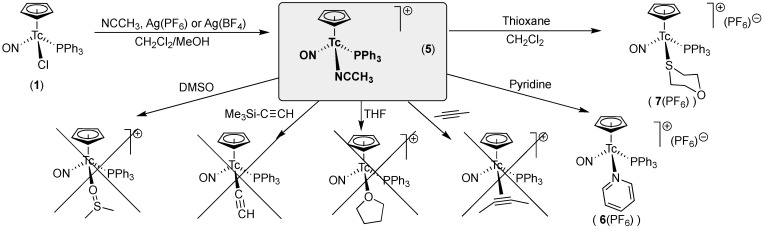
Synthesis of [Tc(NO)(Cp)(PPh_3_)(NCCH_3_)]^+^ and its reactions.

The general procedure for the synthesis of the cationic phosphine chalcogenide complexes **2**–**4** (Scheme 2) also provides a suitable approach to ‘solvent’ complexes, such as [Tc(NO)(Cp)(PPh_3_)(NCCH_3_)]^+^ (**5**), having an acetonitrile ligand. The compound can be prepared by the addition of acetonitrile to a mixture of Ag(PF_6_) and **1** in CH_2_Cl_2_/MeOH and prolonged heating (Scheme 3). The use of the dichloromethane/methanol mixture promotes the dissolution of the starting materials. Long reaction times of several hours are necessary to complete the reaction but also cause problems concerning the purity of the products. Subsequent hydrolysis is observed due to residual water in the solvents and coming from the glass walls, which causes considerable problems with the isolation of the product(s). Particularly, the gradual formation of hydrolysis products of the PF_6_^−^ counter ion proved to be a problem in the isolation of a pure, crystalline product since the formed phosphorus oxyfluorido anions, such as PO_2_F_2_^−^, can act as ligands in competition to acetonitrile and/or partially replace PF_6_^−^ as the counter ion. Similar effects have been observed before for cyclopentadienyl complexes of iridium and rhodium [87,88], but also during the PF_6_^−^ hydrolysis in other media [89]. ^19^F NMR spectra of crude reaction mixtures confirm the gradual decomposition of PF_6_^−^ and the formation of a number of phosphorus-containing compounds. One example with the appearance of new doublets between −65 and −85 ppm is shown in the Supplementary Materials. It confirms the presence of PO_2_F_2_^−^ (with a signal at −81.1 ppm) and HF (with a signal at −137.9 ppm) amongst other degradation products of hexafluorophosphate in such solutions. In a very recent paper, there are also the first reports of the coordination of PO_2_F_2_^−^ ligands originating from the undesired hydrolysis of PF_6_^−^ anions in Tc(I) complexes [49].

**Figure 3:**
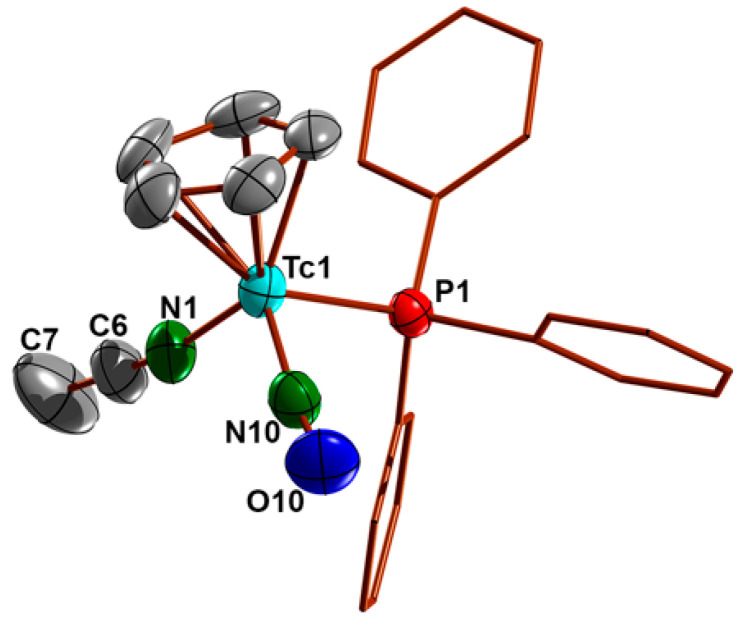
Structure representation of the [Tc(NO)(Cp)(PPh_3_)(NCCH_3_)]^+^ cation (**5**) taken from the BF_4_^−^ salt. Selected bond lengths and angles: Tc1-N10 1.750(4), N10-O10 1.196(5), Tc1-P1 2.385(1), Tc1-N1 2.093(4), N1-C6 1.136(6), C6-C7 1.453(8) Å; Tc1-N10-O10 168.4(4), Tc1-N1-C6 170.4(5)°.

The problems with the synthesis of [Tc(NO)(Cp)(PPh_3_)(NCCH_3_)](PF_6_) in high yields and purity can be avoided by a complete exclusion of water or the use of more robust counter ions. Good results were obtained by replacing Ag(PF_6_) by the corresponding tetrafluoroborate salt. [Tc(NO)(Cp)(PPh_3_)(NCCH_3_)](BF_4_) (**5**(BF_4_)) precipitated as an orange-red solid directly from the reaction mixture. Unlike those obtained for **5**(PF_6_), the single crystals of the BF_4_^−^ salt of compound **5** were of good quality and the corresponding X-ray diffraction study resulted in a dataset, which allowed a refinement with anisotropic thermal parameters. The structure of the complex cation from this refinement is shown in Figure 3 and some important bond lengths are given in the figure caption. Information on the structural data of **5**(PF_6_), with an isotropic refinement only of the carbon, oxygen, and nitrogen positions, is given in the Supplementary Materials.

The bonding situations in both [Tc(NO)(Cp)(PPh_3_)(NCCH_3_)]^+^ salts are similar and unexceptional. They contain almost linearly bonded nitrosyl ligands and the Tc-N1 bond of 2.093(4) Å is of the same magnitude as observed for other Tc(I) complexes with acetonitrile ligands, where they were found between 2.016 and 2.181 Å depending on their *trans* ligands [62,70,90,91,92,93]. The longest Tc-N(acetonitrile) bonds have been found for carbonyl complexes [70,91,92]. But despite the obvious weakening of the Tc-N bonds in such compounds, they are not readily replaced by other monodentate ligands. Such behavior has also been detected for compound **5** of the present study. Attempted reactions with other solvent ligands, such as THF or DMSO, or alkynes (see Scheme 3), did not result in the formation of the targeted ligand-exchange products, even in cases where the corresponding rhenium analogs are well known [94,95,96]. These findings are in a line with reports, in which reactions of compound **1** with various alkynes gave products without any components coming from the alkynes used, such as the dimeric, chlorido-bridged product [{Tc(NO)(Cp)(PPh_3_)}_2_Cl](PF_6_), or compounds with reaction products thereof, such as the carbene-type compound [Tc(NO)(Cp)(PPh_3_){C(OMe)C_2_H_4_PPh_3_}](PF_6_)_2_ [35]. Products with side-on or end-on bonded alkynes, which are common in the corresponding rhenium chemistry, have hitherto not been isolated with the {Tc(NO)(Cp)(PPh_3_)}^+^ fragment. Ligand-exchange reactions starting from **5** with the strong donor pyridine and the thioether thioxane were more successful (vide infra). 

**Figure 4:**
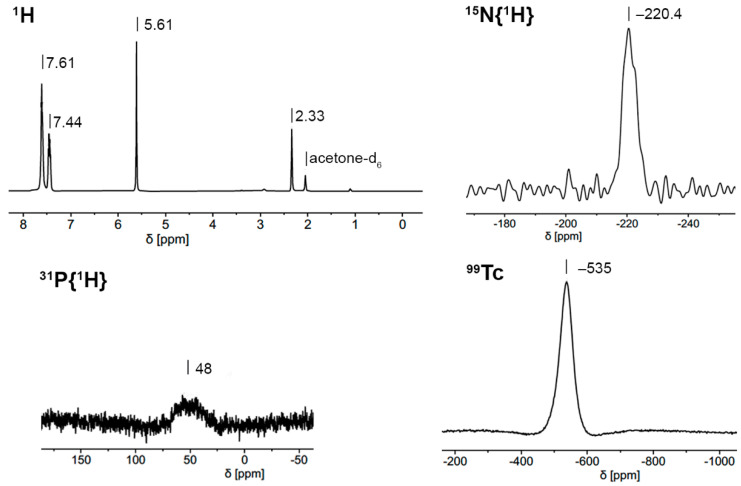
Multinuclear NMR spectra of [Tc(NO)(Cp)(PPh_3_)(^15^N-NCCH_3_)](BF_4_) (**5**(BF_4_)) in acetone-d_6_. For full range spectra see Supplementary Materials.

Having in mind the relative inertness of [Tc(NO)(Cp)(PPh_3_)(NCCH_3_)]^+^, some experiments were designed to understand this behavior. First, we synthesized compound **5**(BF_4_) with ^15^N-labeled acetonitrile (^15^N-enrichment: 95%), ^15^N-**5**(BF_4_) in order to study the exchange of the NCCH_3_ ligands by means of ^15^N NMR spectroscopy. ^15^N-**5**(BF_4_) could be isolated as an orange-red solid. Figure 4 depicts the NMR spectra of the compound. The proton spectrum clearly shows the expected narrow signals for the diamagnetic d^6^ complex, while the lines observed for the hetero nuclei ^15^N and ^31^P are remarkably broadened. Similar observations have been made before for other (non-symmetric) phosphine complexes of technetium(I), -(III), and -(V), and have been assigned to scalar couplings of the ^31^P nucleus with the large quadrupole moment of ^99^Tc [34,59,61,62,81,97,98,99]. The resulting line broadenings make the detection of reasonable ^31^P NMR spectra in some cases almost impossible (see also Figure 4). Unexpectedly, this is also observed for the ^15^N{^1^H} signal of the coordinated acetonitrile ligand in compound **5**. It appears at −220 ppm with a half-line width (ν_1/2_) of ≈215 Hz, while the signal of the uncoordinated acetonitrile is detected at −134.1 ppm with a ν_1/2_ value of only 5.5 Hz (see Supplementary Materials). The ^15^N line width in ^15^N-**5**(BF_4_) is similar to that observed for the ^15^N ammine ligand in [Tc(^15^N-NH_3_)_2_(CO)_2_(PPh_3_)_2_](BF_4_) (≈150 Hz) [62]. The difference between the ^15^N chemical shifts and the line widths of coordinated and uncoordinated acetonitrile allows the evaluation of the corresponding ligand exchange by the ready detection of the narrow signal of the released ^15^N-NCCH_3_ when [Tc(NO)(Cp)(PPh_3_)(^15^N-NCCH_3_)](BF_4_) is treated with acetonitrile having the natural isotope abundance. It became obvious that this ligand exchange is very slow at room temperature and higher exchange rates are only available at elevated temperatures. ^15^N{^1^H} NMR spectra monitoring such reactions are shown in the Supplementary Materials. They illustrate (a) that after stirring a mixture of ^15^N-**5**(BF_4_) and one equivalent of acetonitrile with natural isotope abundance in acetone at room temperature, less than 10 percent of the coordinated ^15^N-acetonitrile is released within several hours, and (b) subsequent heating on reflux increases the amount of released ^15^N-acetonitrile.

**Figure 5:**
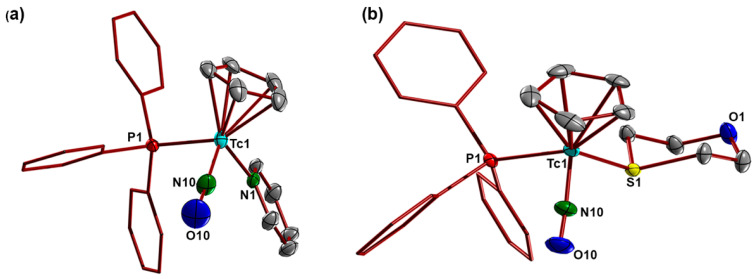
Structure representations of (**a**) the [Tc(NO)(Cp)(PPh_3_)(py)]^+^ (**6**) and (**b**) the [Tc(NO)(Cp)(PPh_3_)(*S*-thioxane)]^+^ (**7**) cations.

The slow exchange of the acetonitrile ligand nicely explains the failed reactions of the acetonitrile complex **5** with other donor solvents, such as THF or DMSO, or with alkynes (Scheme 3). Although the exchange of the acetonitrile is slow, reactions with a large excess of pyridine or thioxane led to the corresponding ligand exchange products [Tc(NO)(Cp)(PPh_3_)(py)](PF_6_) (**6**(PF_6_)) and [Tc(NO)(Cp)(PPh_3_)(*S*-thioxane)](PF_6_) (**7**(PF_6_)) in satisfactory yields in room temperature-reactions when a large excess of the ligands was used. Orange-red to red single crystals of the PF_6_^−^ salts were obtained after removal of the solvents/reactants from CH_2_Cl_2_/diethyl ether mixtures. Figure 5 depicts the corresponding complex cations and selected bond lengths and angles are summarized in Table 2.

The structures of the two complex cations are unexceptional and the Tc-N1 and Tc-S2 bonds (2.161 and 2.405 Å) are similar to other Tc(I)-pyridine (2.141–2.249 Å) [62,68,82,100,101] and Tc(I)-thioether (2.440–2.541 Å) bonds [62,102,103,104]. The coordination of thioxane via its thioether unit is not unexpected given that there are very few technetium complexes with certainly proved ether ligands [66,105,106] and none of them contain a ‘soft’ technetium(I) metal ion. Calculations at the B3LYP/Stuttgart 1997(Tc)/LANL2DZ(N,P,S)/6-311 + G**(remaining atoms) level indicate a clear thermodynamic preference of the *S*-bonded isomer with a free energy difference of 40 kJ/mol in the gas phase, while it is lower in an implicit solvent model for THF (30 kJ/mol). Contributions from *O*-bonded thioxane to an on-going equilibrium are, therefore, expected to play a role, if any, only at highly elevated temperatures (Boltzmann factor at 120 °C in solution: 2 × 10^−2^, in the gas-phase: 5 × 10^−4^). The theoretical, calculated stabilization energy of the complexation of *S*-thioxane to the hypothetical {Tc(NO)(Cp)(PPh_3_)}^+^ fragment is 24 kJ/mol, while the ΔΔ*G* is destabilizing by 5 kJ/mol for the oxygen-coordinated isomer, suggesting a ready dissociation of the *O*-bound ligand. In comparison, the acetonitrile complex shows a much higher stabilizing ΔΔ*G* of complexation of 83 kJ/mol, which explains the required high temperatures for ligand exchange procedures in a preferential dissociative mechanism combined with kinetic trapping by the large excess of incoming pyridine or thioether, which form less stable complexes. The stabilization of the unsaturated fragment by pyridine is ca. 70 kJ/mol, while the formation of the dimeric chlorido-bridged complex is similar to the stabilization by thioxane with 23 kJ/mol. In contrast, the chlorido ligand in **1** is bound very tightly with much higher complexation energies, which may be partially attributed to charge compensation. We envision that systematic computational studies, combined with the development of systematic, theoretical models for the ^99^Tc NMR parameters, will guide and enhance our understanding of the ligand exchange processes at the {Tc(NO)(Cp)(PPh_3_)}^+^ fragment in the future.

To confirm the exclusive formation of the S-bonded isomer of compound **7**, we recorded ^99^Tc NMR spectra of the reaction mixture during the ligand exchange between **5** and thioxane. This method is a suitable tool to monitor such reactions, since the chemical shifts of such signals are sensitive to the composition of the coordination sphere of complexes with the [Tc(NO)(Cp)(PPh_3_)(L)]^0,+^ skeleton (for a more detailed discussion about this point see Section 2.4 of the present paper). In the course of the reaction, the signal of the acetonitrile complex **5** at −534 ppm gradually decreases in intensity and that of the S-bonded compound **7** increases at −866 ppm. There was no evidence for the formation of an O-bonded compound at room temperature.

### 2.3. [Tc(NO)(Cp^R^)(PPh_3_)(L)]^0,+^ Complexes

One of the main motivations to foster synthetic technetium chemistry is the use of the metastable nuclide ^99m^Tc in diagnostic nuclear medicine [107]. In recent years, organometallic approaches with arene or cyclopentadienyl ligands have also been considered for such applications [107,108,109,110,111,112,113]. Molecules relevant for such applications frequently possess a reactive group in their periphery on which targeting biomolecules can be attached. Carboxylic or amino groups allow common peptide couplings, but also click reactions and the formation of thiourea linkers via isocyanates have been utilized [107,114,115,116].

**Scheme 4:**
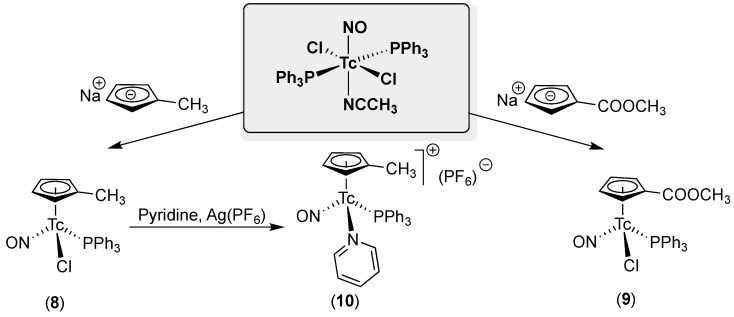
Synthesis of [Tc(NO)(Cp^R^)(PPh_3_)(L)]^0,+^ complexes (L = Cl^−^, py; R = Me, COOCH_3_).

Although reactions with the short-lived ^99m^Tc (pure γ-emitter, half-life: 6 h) are not the subject of the present publication, we undertook a few ‘proof of principle’ experiments with methyl- and methylcarboxylato-substituted cyclopentadienides to demonstrate that the shown reactivity is not restricted to unsubstituted cyclopentadienides. Thus, we isolated [Tc(NO)(Cp^Me^)(PPh_3_)Cl] (**8**) and [Tc(NO)(Cp^COOMe^)(PPh_3_)Cl] (**9**) and reacted compound **8** with pyridine and AgPF_6_ giving [Tc(NO)(Cp^Me^)(PPh_3_)(py)](PF_6_) (**10**(PF_6_), Scheme 4). 

Complexes **8** and **9** can be prepared following the procedure described for complex **1** by prolonged heating of the sodium salts of the substituted cyclopentadienyls with [Tc(NO)Cl_2_(PPh_3_)_2_(NCCH_3_)] in toluene. After extraction with CH_2_Cl_2_ and filtration over silica, the red solids can be recrystallized from CH_2_Cl_2_/hexane solutions. The IR stretches of ν_NO_ 1684 cm^−1^ (**8**) and 1697 cm^−1^ (**9**), as well as the ^99^Tc NMR resonances of −277 ppm (**8**) and −260 ppm (**9**), recorded for the product are very similar to the ones obtained for compound **1** (IR: ν_NO_ 1682 cm^−1^, ^99^Tc: NMR −231 ppm), which indicates that the substitution on the Cp ring has only a small influence on the electronic situation of the technetium atom and the nitrosyl ligand. Due to the introduction of the substituents at the Cp rings, the symmetry becomes lower by hindering the rotation of the corresponding ring. Thus, the proton signals in the ^1^H NMR spectra of the novel complexes are resolved into four resonances in the chiral complexes under study. Similar findings have been described before for some iron complexes [117,118]. 

A reaction of the chlorido complex **8** with AgPF_6_ and pyridine in a CH_2_Cl_2_/MeOH mixture gives the pyridine complex **10**(PF_6_) in reasonable yields. The red product is soluble in CHCl_3_ and its ^99^Tc NMR appears at almost the same chemical shift and line widths, like its unsubstituted analog **6**(PF_6_) described above (compound **6**: −287 ppm, ν_1/2_ = 4700 Hz; compound **10**: −301 ppm, ν_1/2_ = 4850 Hz), indicating no significant influence of the substitution on the electronic situation at the central technetium atom. The proton NMR spectrum shows the same splitting of the signals of the Cp^Me^ protons due to the hindered rotation around the Cp-Tc axis as discussed for compound **8**. 

**Figure 6:**
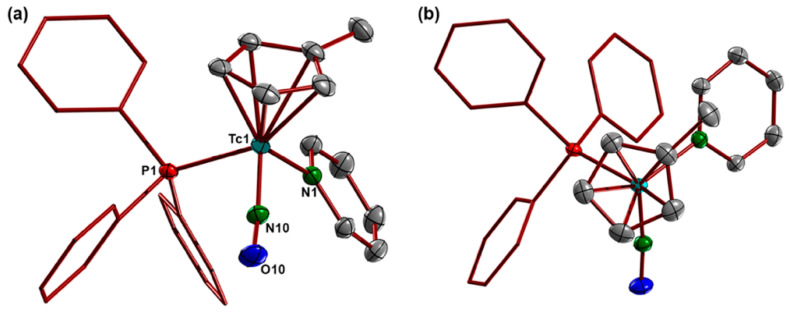
Structure representations of the [Tc(NO)(Cp^Me^)(PPh_3_)(py)]^+^ cation of **10**(PF_6_): (**a**) labeling scheme, (**b**) top view. Selected bond lengths and angles: Tc1-N10 1.762(2), N10-O10 1.175(3), Tc1-P1 2.3925(7), Tc1-N1 2.161(3) Å; Tc1-N10-O10 169.3(2)°.

Orange-red single crystals of [Tc(NO)(Cp^Me^)(PPh_3_)(py)](PF_6_) suitable for X-ray diffraction were grown by slow diffusion of diethyl ether into a solution of the complex in CH_2_Cl_2_. The structure of the complex cation is depicted in Figure 6. The bond lengths and angles are unexceptional and similar to those in **6**. The top view shows that the methyl substituent of the Cp ring is arranged in a position in which steric interactions with the PPh_3_ ligand are avoided, resulting in the discussed rigidity and, hence, the splitting of the ^1^H resonances for the aromatic C-H protons of the Cp ring.

With the isolation of complexes **8** to **10**, there is proof that technetium compounds of the general type [Tc(NO)(Cp^R^)(PPh_3_)(L)]^0/+^ are also accessible for substituted cyclopentadienyl derivatives. This finding is not as trivial as it seems to be. Even the large collection of corresponding rhenium complexes [Re(NO)(Cp^R^)(PPh_3_)(L)]^0,+^, which can be found in the Cambridge Crystallographic Database with almost 200 entries, contains only three examples with monosubstituted (two methyl, one iodide) cyclopentadienyl ligands [119,120,121]. Particularly, the isolation of complex **9** with a molecular position for the attachment of a biomarker might stimulate future studies with ^99m^Tc towards potential applications in nuclear medicine.

### 2.4. ^99^Tc NMR Spectra of Complexes with {Tc(NO)(Cp)(PPh_3_)}^+^ Units

The nuclear properties of ^99^Tc (nuclear spin I = 9/2, magnetic moment 6.281 µ/µ_N_, gyromagnetic ratio 6.046 γ/10^7^ rad s^−1^ T^−1^ [122]) make this nucleus a valuable tool for the evaluation of diamagnetic technetium compounds and reaction mixtures, in which such species play a role as educt, product, or short-lived intermediate. A substantial drawback is the noticeable quadrupole moment of this nucleus (Q = −0.19(5) × 10^−28^ m^2^ [123]), which leads to a considerable line-broadening, especially for compounds having low local symmetry around the technetium nucleus. This drawback, however, is clearly compensated by the high sensitivity of the ^99^Tc resonances (relative receptivity of about 0.275 to ^1^H [59]), which allows the ready detection of ^99^Tc signals, and the fact that ^99^Tc NMR signals appear in an extremely large chemical shift range of several thousand ppm. Thus, ^99^Tc NMR signals of technetium(I) compounds can be unambiguously detected even when they show line widths of more than 15 kHz [124]. 

**Figure 7:**
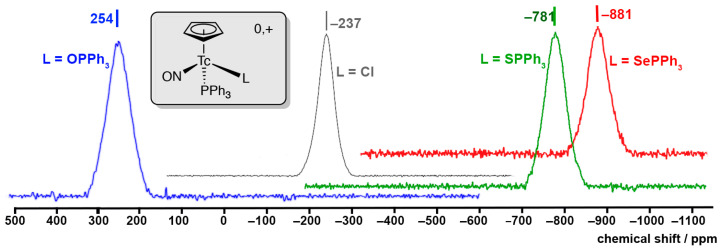
^99^Tc NMR spectra of [Tc(NO)(Cp)(PPh_3_)(OPPh_3_)]^+^ (**2**), [Tc(NO)(Cp)(PPh_3_)(SPPh_3_)]^+^ (**3**), and [Tc(NO)(Cp)(PPh_3_)(SePPh_3_)]^+^ (**4**), together with that of the starting material **1**.

The ^99^Tc NMR signals of the [Tc(NO)(Cp^R^)(PPh_3_)(L)]^0,+^ complexes of the present study show line widths between 3000 and 7000 Hz, which means that they are also relatively broad. Nevertheless, they allow the ready monitoring of ligand exchange reactions. Figure 7 shows the spectra of the series of [Tc(NO)(Cp^R^)(PPh_3_)(EPPh_3_)]^+^ complexes (E = O, S, Se) together with the spectrum of the starting material [Tc(NO)(Cp)(PPh_3_)Cl] (**1**). It is evident that the chemical shifts cover a large range and the shielding/deshielding of the technetium nucleus is strongly dependent on the individual donor atoms. In the present case, these differences are due to only one atom of the EPPh_3_ ligands, which causes chemical shift differences of more than 1000 ppm when going from triphenylphosphine oxide via the sulfide to the selenide. The observed trend also endures for ligands having donor atoms coming from other groups of the Periodic Table. 

**Figure 8:**
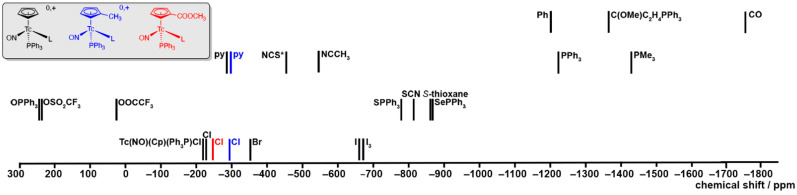
^99^Tc NMR chemical shifts recorded for [Tc(NO)(Cp)(PPh_3_)(L)]^0,+^ complexes. * The structure of the isothiocyanato complex has not yet been characterized by X-ray diffractometry. The used colors refer to the formulae shown in the box.

Figure 8 depicts a summary of the values measured for all the structurally characterized representatives of [Tc(NO)(Cp^R^)(PPh_3_)(L)]^0,+^ complexes, which span a remarkably large chemical shift range with the carbonyl compound having the most negative chemical shift at −1753 ppm [33]. It becomes clear that the trend observed for the phosphine chalcogenides is a general one. Thus, the sequence of the chemical shift within a related series can readily be predicted by the basic physical properties of the donors, such as the electronegativity of the donors (or the atom weight or Pearson hard/soft scale [125] or donor/acceptor properties). This is observed for the extended family of chalcogen donors (also including carboxylic and sulfonic acids, thiocyanate, and thioethers), but also for the series of halides and related ligands, where Cl^−^- and {Tc(NO)(Cp)PPh_3_)Cl}-substituted complexes appear at about −220 ppm, while the signals of the bromido and iodido species are clearly high-field shifted. Unfortunately, the corresponding fluorido compound has not yet been isolated and cannot be used for comparison. A similar trend, however, is observed for the pnictogens, where phosphines cause a higher shielding of the ^99^Tc nucleus than pyridine or N-bonded isothiocyanate.

The systematic trend in the ^99^Tc chemical shifts observed for the [Tc(NO)(Cp^R^)(PPh_3_)(L)]^0,+^ complexes following the general physical properties of the donor atoms is obvious and should be studied in more detail for the quantification of the observed effects. Probably, future DFT considerations can help to deliver reliable predictions for the entire series of compounds, as has similarly been demonstrated for the [Tc(NO)(Cp)(PPh_3_)(EPPh_3_)]^+^ (E = O, S, Se) complexes discussed above.

## 3. Materials and Methods

Unless otherwise stated, reagent-grade starting materials were purchased from commercial sources and either used as-received or purified by standard procedures. Solvents were dried and distilled prior to use. [Tc(NO)Cl_2_(PPh_3_)_2_(MeCN)] [31], [Tc(NO)Cl(Cp)(PPh_3_)] [33], KCp [126], Na(CpMe) [127] and Na(CpCOOMe) [128] were prepared as described in the literature. 

### 3.1. Radiation Precaution

All synthetic work with the long-lived isotope ^99^Tc was performed in a laboratory approved for the handling of radioactive material. The glass walls of the flasks provide appropriate protection of the primary beta emission of ^99^Tc. Secondary X-rays (bremsstrahlung) become important only when larger amounts of the compounds are handled as solids. All personnel working on this project were permanently monitored for potential contamination. 

### 3.2. Syntheses

*[Tc(NO)(Cp)(PPh_3_)(OPPh_3_)](PF_6_) (**2**(PF_6_)).* [Tc(NO)(Cp)(PPh_3_)Cl] (50 mg, 0.1 mmol) was dissolved in 10 mL CH_2_Cl_2_ and treated with a solution of AgPF_6_ (25 mg, 0.1 mmol) in 2 mL CH_2_Cl_2_/MeOH (2/1, *v*/*v*). A grey precipitate was formed. Triphenylphosphine oxide (28 mg, 0.1 mmol) was added to the reaction mixture, which was then heated under reflux for 3 h. The resulting mixture was filtered and the solvent was removed under vacuum. The residue was dissolved in 1 mL CH_2_Cl_2_, and the solution was covered with 3 mL diethyl ether. Red crystals were formed after slow diffusion of the solvents. They were isolated by filtration and dried in air. Yield 42 mg (49%). IR (KBr, cm^−1^): 3431(w), 3057(w), 2920(w), 1703(vs), 1481(w), 1437(vs), 1312(w), 1121(s), 1094(m), 1069(s), 1026(m), 839(vs), 748(m), 725(s), 694(s), 541(vs). ^1^H NMR (CDCl_3_, ppm): 7.73–7.25 (m, PPh_3_, OPPh_3_; overlapping with impurities, such as uncoordinated OPPh_3_), 4.84 (s, 5H, Cp). ^31^P{^1^H} NMR (CD_2_Cl_2_, ppm): 57.8 (s, OPPh_3_), 49 (very broad, PPh_3_), 144.5 (sept. J_P-F_ = 711 Hz, PF_6_). ^99^Tc NMR (CD_2_Cl_2_, ppm): 254.3 (ν_1/2_ = 5160 Hz).

*[Tc(NO)(Cp)(PPh_3_)(SPPh_3_)](PF_6_) (**3**(PF_6_)).* [Tc(NO)(Cp)(PPh_3_)Cl] (50 mg, 0.1 mmol) was dissolved in 10 mL CH_2_Cl_2_ and treated with a solution of AgPF_6_ (25 mg, 0.1 mmol) in 2 mL CH_2_Cl_2_/MeOH (2/1, *v*/*v*). A grey precipitate was formed. Triphenylphosphine sulfide (30 mg, 0.1 mmol) was added to the reaction mixture, which was then heated under reflux for 3 h, filtered and the solvent was removed under vacuum. The remaining residue was dissolved in 1 mL CH_2_Cl_2_ and covered with 3 mL diethyl ether. Red crystals were formed after slow diffusion of the solvents. They were isolated by filtration and dried in air. Yield: 39 mg (43%). IR (KBr, cm^−1^): 3431(w), 3037(w), 2918(w), 1705(vs), 1479(w), 1436(s), 1313(w), 1184(m), 1099(ss), 1072(w), 1028(w), 999(w), 839(vs), 748(m), 694(s), 586(m), 557(m), 524(m). ^1^H NMR (CD_2_Cl_2_, ppm): 7.75–7.40 (m, 30H, PPh_3_, SPPh_3_), 4.73 (s, 5H, Cp) ppm. ^31^P{^1^H} NMR (CD_2_Cl_2_, ppm): 50.2 (s, SPPh_3_), 50 (very broad, PPh_3_), 146.1 (sept. J_P-F_ = 711 Hz, PF_6_). ^99^Tc NMR (CD_2_Cl_2_, ppm): −780.5 (ν_1/2_ = 4870 Hz).

*[Tc(NO)(Cp)(PPh_3_)(SePPh_3_)](PF_6_) (**4**(PF_6_)).* [Tc(NO)(Cp)(PPh_3_)Cl] (50 mg, 0.1 mmol) was dissolved in 10 mL CH_2_Cl_2_ and treated with a solution of AgPF_6_ (25 mg, 0.1 mmol) in 2 mL CH_2_Cl_2_/MeOH (2/1, *v*/*v*). A grey precipitate was formed. Triphenylphosphine selenide (35 mg, 0.1mmol) was added and the reaction mixture was heated under reflux for 4 h. The solution was filtered, and the solvent was removed under vacuum. The residue was dissolved in 1 mL CH_2_Cl_2_ and covered with 3 mL diethyl ether. Orange-red crystals were formed after slow diffusion of the solvents. They were isolated by filtration and dried in air. Yield: 35 mg (38%). IR (KBr, cm^−1^): 3448(m), 3055(w), 2918(w), 1703(vs), 1479(m), 1437(vs), 1314(w), 1184(w), 1098(s), 999(w), 839(vs), 748(m), 692(s), 557(m), 540(m), 527(m), 511(m). ^1^H NMR (CD_2_Cl_2_, ppm): 7.74–736 (m, 30H, PPh_3_, SePPh_3_), 4.71 (s, 5H, Cp). ^31^P{^1^H} NMR (CD_2_Cl_2_, ppm): 30.0 (s, SePPh_3_), 48 (very broad, PPh_3_), 146.2 (sept. J_P-F_ = 711 Hz, PF_6_). ^99^Tc NMR (CD_2_Cl_2_, ppm): −881.2 (ν_1/2_ = 5080 Hz). 

*[Tc(NO)(Cp)(PPh_3_)(NCCH_3_)](PF_6_) (**5**(PF_6_)).* [Tc(NO)(Cp)(PPh_3_)Cl] (25 mg, 0.05 mmol) was dissolved in 2 mL CH_2_Cl_2_ and treated with a solution of AgPF_6_ (25 mg, 0.05 mmol) in 2 mL CH_2_Cl_2_/MeOH (2/1, *v*/*v*). CH_3_CN (15 mL) was added to the deep red solution and the reaction mixture was heated under reflux for 3 h. The solution was filtered, and the solvent was removed under vacuum. The residue was dissolved in 1 mL CH_2_Cl_2_ and covered with 4 mL *n*-hexane. Orange-red crystals were formed after slow diffusion of the solvents. They were isolated by filtration and dried in air. Yield: 17 mg (52%). IR (KBr, cm^−1^): 3397(w), 3126(w), 3061(w), 2938(w), 1707(vs), 1497(w), 1437(m), 1312(w), 1095(m), 841(vs), 756(m), 694(m), 604(m), 586(w), 557(m), 526(m). ^1^H NMR (CD_2_Cl_2_, ppm): 7.55–7.30 (m, 15H, Ph), 5.40 (s, 5H, Cp), 2.18 (s, 3H, CH_3_). ^31^P{^1^H} NMR (CD_2_Cl_2_, ppm): ≈50 (extremely broad, PPh_3_), 146.2 (sept. J_P-F_ = 711 Hz, PF_6_). ^99^Tc NMR (CD_2_Cl_2_, ppm): −536.7 (ν_1/2_ = 3800 Hz). 

*[Tc(NO)(Cp)(PPh_3_)(NCCH_3_)](BF_4_) (**5**(BF_4_)).* The compound was prepared as the PF_6_^−^ salt by using AgBF_4_ instead of AgPF_6_. Orange-red crystals. Yield: 50%. IR (KBr, cm^−1^): 3397(w), 3126(w), 3061(w), 2938(w), 1707(vs), 1497(w), 1437(m), 1312(w), 1095(m), 841(vs), 756(m), 694(m), 604(m), 586(w), 557(m), 526(m). ^1^H NMR (acetone-d_6_, ppm): 7.55–7.30 (m, 15H, Ph), 5.40 (s, 5H, Cp), 2.18 (s, 3H, CH_3_). ^31^P{^1^H} NMR (acetone-d_6_, ppm): ≈50 (extremely broad, PPh_3_). ^99^Tc NMR (acetone-d_6_, ppm): −535.3 (ν_1/2_ = 5900 Hz).

*[Tc(NO)(Cp)(PPh_3_)(^15^NCCH_3_)](BF_4_) (^15^N-**5**(BF_4_)).* The compound was prepared as **5**(BF_4_) using ^15^N-labeled acetonitrile (95% enrichment). Orange-red crystals. Yield: 50%. ^15^N NMR (acetone-d_6_, ppm): −220.4 (ν_1/2_ = 215 Hz).

*[Tc(NO)(Cp)(PPh_3_)(py)](PF_6_) (**6**(PF_6_)).* [Tc(NO)(Cp)(PPh_3_)(NCCH_3_)](PF_6_) (32 mg, 0.05 mmol) was dissolved in 2 mL CH_2_Cl_2_. Pyridine (0.5 mL) was added, and the mixture was stirred for 1 h at room temperature. The solvent was removed under vacuum. The residue was dissolved in 1 mL CH_2_Cl_2_ and covered with 4 mL diethyl ether. Orange-red single crystals were formed after slow diffusion of the solvents. They were isolated by filtration and dried in air. Yield: 22 mg (64%). IR (KBr, cm^−1^): 3410(w), 3116(w), 3060(w), 2920(w), 1719(vs), 1481(m), 1437(m), 1311(m), 1186 (w), 1070(w), 841(vs), 750(m), 696(s), 585(w), 557(m), 527(m), 509(w). ^1^H NMR (CD_2_Cl_2_, ppm): 8.26–7.16 (m, 20H, Ph), 5.43 (s, 5H, Cp). ^31^P{^1^H} NMR (CDCl_3_, ppm): 35 (very broad, PPh_3_), 146.2 (sept. J_P-F_ = 711 Hz, PF_6_). ^99^Tc NMR (CDCl_3_, ppm): −286.6 (ν_1/2_ = 4700 Hz).

*[Tc(NO)(Cp)(PPh_3_)(S-thioxane)](PF_6_) (**7**(PF_6_)).* [Tc(NO)(Cp)(PPh_3_)(CH_3_CN)](PF_6_) (32 mg, 0.05 mmol) was dissolved in 2 mL CH_2_Cl_2_ and thioxane (0.5 mL) was added. The reaction mixture was stirred for 2 h at room temperature and the solvent was removed under vacuum. The residue was dissolved in 1 mL CH_2_Cl_2_ and covered with 4 mL diethyl ether. Red single crystals were formed after slow diffusion of the solvents. They were isolated by filtration and dried in air. Yield: 19 mg (55%). IR (KBr, cm^−1^): 3456(w), 3061(w), 2922(w), 2859(w), 1742(vs), 1479(m), 1435(m), 1411(w), 1382(w), 1311(w), 1279(m), 1186(m), 1061(w), 997(w), 1049(vs), 997(m), 966(m), 840(m), 829(m), 752(s), 694(s), 588(m), 527(s), 505(m), 448(w). ^1^H NMR (CDCl_3_, ppm): 7.56–7.31 (m, 15, Ph), 5.42 (s, 5H, Cp), 3.79 (m, 4H, thioxane), 2.58–2.40 (m, 4H, thioxane). ^31^P{^1^H} NMR (CDCl_3_, ppm): 53.0 (very broad, PPh_3_), 144.5 (sept. J_P-F_ = 711 Hz, PF_6_). ^99^Tc NMR (CDCl_3_, ppm): −871 (ν_1/2_ = 5400 Hz).

*[Tc(NO)(Cp^Me^)(PPh_3_)Cl] (**8**).* [Tc(NO)Cl_2_(PPh_3_)_2_(NCCH_3_)] (230 mg, 0.3 mmol) was suspended in 5 mL toluene. Na(Cp^Me^) (102 mg, 1 mmol) was dissolved in 5 mL toluene and added to the light orange-red suspension. The reaction mixture was heated under reflux for 2 h. The solvent was removed under vacuum and the red residue was dissolved in CH_2_Cl_2_ (2 mL) and filtered over a 2 cm layer of silica gel. *n*-Hexane (2 mL) was added, and the solvents were slowly evaporated. The resulting red solid of [Tc(NO)(Cp^Me^)(PPh_3_)Cl] was isolated by filtration. Yield. 110 mg (66%). IR (KBr, cm^−1^): 3445(w), 3055(w), 2963(m), 2922(w), 2855(w), 1686(vs), 1479(m), 1433(s), 1307(w), 1261(vs), 1179(m), 1094(vs), 1024(vs), 835(s), 803(vs), 746(m), 694(s), 538(m), 499(m). ^1^H NMR (CDCl_3_, ppm): 7.67–7.28 (m, Ph; overlapping with OPPh_3_-based impurities), 5.14 (1H, Cp), 4.97 (1H, Cp), 4.63 (1H, Cp), 4.49 (1H, Cp), 1.82 (3H, Cp-CH_3_). ^31^P{^1^H} NMR (CDCl_3_, ppm): 27.3 (s, PPh_3_). ^99^Tc NMR (CDCl_3_, ppm): −277 (ν_1/2_ = 4580 Hz). 

*[Tc(NO)(Cp^COOMe^)(PPh_3_)Cl] (**9**).* [Tc(NO)Cl_2_(PPh_3_)_2_(NCCH_3_)] (150 mg, 0.2 mmol) was suspended in 5 mL toluene. Na(Cp^COOMe^) (100 mg, 0.7 mmol) was dissolved in 5 mL toluene and added to the light orange-red suspension. The reaction mixture was heated under reflux for 2 h. The solvent was removed under vacuum. The red residue was dissolved in CH_2_Cl_2_ (2 mL) and filtered over a 2 cm layer of silica gel. *n*-Hexane (2 mL) was added, and the solvents were slowly evaporated. The resulting deep red solid was isolated by filtration and dried in vacuo. Yield: 70 mg (63%). IR (KBr, cm^−1^): 3393(w), 3053(m), 2959(w), 2920(w), 1697(vs), 1479(s), 1433(vs), 1286(s), 1190(s), 1144(m), 1117(m), 1094(m), 1026(m), 804(m), 756(s), 723(m), 694(vs), 540(vs), 449(w). ^1^H NMR (CDCl_3_, ppm): 7.65–7.27 (m, PPh_3_ overlapping with impurities due to toluene, OPPh_3_ and PPh_3_), 5.77 (1H, dt, *J* = 3.37 Hz and 1.73 Hz, Cp-H), 5.40 (1H, td, *J* = 3.30 Hz and 1.81 Hz, Cp-H), 5.05 (1H, td, *J* = 2.98 Hz and 1.80 Hz, Cp-H), 4.99 (1H, q, *J* = 2.59 Hz, Cp-H), 3.68 (3H, Cp-CH_3_). ^31^P{^1^H} NMR (CDCl_3_, ppm): 40.9 (broad, PPh_3_). ^99^Tc NMR (CDCl_3_, ppm): −260.2 (ν_1/2_ = 3350 Hz). 

*[Tc(NO)(Cp^Me^)(PPh_3_)(py)](PF_6_) (**10**(PF_6_)).* [Tc(NO)(Cp^Me^)(PPh_3_)Cl] (55 mg, 0.1 mmol) was dissolved in 2 mL CH_2_Cl_2_ and treated with a solution of AgPF_6_ (25 mg, 0.1 mmol) in 2 mL CH_2_Cl_2_/MeOH (2/1, *v*/*v*). A grey precipitate was formed. Pyridine (0.5 mL) was added, and the reaction mixture was stirred at room temperature for 2 h. The solution was filtered, and the solvent was removed under vacuum. The residue was dissolved in 1 mL CH_2_Cl_2_ and covered with 4 mL diethyl ether. Orange-red crystals were formed after slow diffusion of the solvents. They were isolated by filtration and dried in air. Yield: 44 mg (62%). IR (KBr, cm^−1^): 3445(vs), 3055(w), 2961(w), 2922(w), 2854(w), 1678(vs), 1481(m), 1435(s), 1261(w), 1186(m), 1119(s), 1094(m), 1026(w), 841(s), 748(m), 722(m), 694(vs), 540(s). ^1^H NMR (CDCl_3_, ppm): 8.33–7.10 (m, Ph, py overlapping with impurities due to uncoordinated pyridine and PPh_3_ and OPPh_3_), 5.42 (1H, s, Cp), 5.34 (1H, s, Cp), 5.18 (1H, s, Cp), 4.99 (1H, s, Cp), 1.69 (3H, CH_3_). ^31^P{^1^H} NMR (CDCl_3_, ppm): 10.0 (s, PPh_3_), 145.2 (sept. J_P-F_ = 711 Hz, PF_6_). ^99^Tc NMR (CDCl_3_, ppm): −301 (ν_1/2_ = 4850 Hz).

### 3.3. Spectroscopic Methods

IR spectra were recorded on a Shimadzu IR Affinity-1 spectrometer (Shimadzu, Kyoto, Japan) measured as KBr pellets with 50 scans per sample (resolution: 4 cm^−1^) between 400 and 4000 cm^–1^. Multinuclear NMR spectra were recorded on JEOL ECS 400 and JEOL ECZ 400 spectrometers (JEOL, Kyoto, Japan). A solution of KTcO_4_ in D_2_O was used as reference for the ^99^Tc spectra. 

### 3.4. X-ray Crystallography

X-ray data were collected on a STOE IPDS-2T (STOE, Darmstadt, Germany) or Bruker CCD instruments (Bruker, Billerica, MA, USA) with Mo/Kα radiation. SADABS or X-RED32 were used for absorption correction [129,130]. The SHELX programs [131,132] included in the WinGX [133] or OLEX2 [134] program packages were used for structure solution and refinement. The ‘riding model’ option of SHELXL was used to treat the hydrogen atoms. Diffuse and/or strongly disordered solvent molecules were treated using the SQUEEZE option installed in the program PLATON or the solvent mask option of OLEX2. Details are outlined in the Supplementary Materials. Molecular graphics were produced with the program DIAMOND, vers. 5.0 [135].

### 3.5. Computational Chemistry

DFT calculations were performed on the high-performance computing systems of the Freie Universität Berlin ZEDAT (Soroban, Curta) using the program package GAUSSIAN 16 Rev. A.03 [136]. Units are provided as atomic units ([a.u.]) unless specified. The gas-phase geometry optimizations in vacuum were performed using coordinates derived from the X-ray crystal structures or were modeled with the use of crystal structure fragments using GAUSSVIEW, while initial guesses for calculations involving an implicit polarizable continuum model with integral equation formalism (IEF-PCM) for the solvent tetrahydrofurane were derived from the gas-phase optimized structures [137]. The calculations were performed by using the hybrid density functional B3LYP [138,139,140]. The relativistic small-core basis set Stuttgart RSC 1997 with the respective effective core potential (ECP) was applied to Tc [141,142]. The double-ζ pseudopotential LANL2DZ basis set was applied to N, P, S, Se, and Cl with the respective ECP for the heavier atoms [143,144]. The 6-311+G** basis set was applied for all other atoms [145,146]. The hybrid functional B3LYP was chosen based on robustness and with regard to a compromise between computational cost and reliable geometry optimization. Although relativistic effects should be small for Tc, as stated in the original benchmarking of the basic constituents of the Stuttgart basis set and ECP, the relativistic small core basis set was proven as a robust yet versatile basis set for Tc in B3LYP (see e.g., [34,61,63,72]). For all other atoms, the choice of the basis functions was a compromise between size/accuracy and computational time, and although the results do not differ much, we preferably used the bigger LANL2DZ for donor atoms directly coordinated to technetium due to its increased accuracy (see e.g., [34,61,63,72]). NMR tensors were calculated for the B3LYP-level optimized gas-phase structures using the B3P86 functional [139,147], combined with the dedicated all-electron NMR basis set x2c-TZVPPall-s for all atoms [148]. Similar approaches have recently been suggested; however, such methods commonly revolve around the exact replication of experimental chemical shifts by exact modeling of solvent, relativistic, and quadrupolar effects with methods of high computational cost and expert knowledge requirements [149,150,151,152,153,154], whereas the presented approach of using low-cost functionals combined with low-cost modern basis sets is of much lower computational cost that could allow routine implementation to complement experimental studies by non-experts after in-depth benchmarking. Such benchmarking is currently on-going, and a corresponding manuscript is in preparation. All basis sets and ECPs were obtained from the basis set exchange database [155].

The convergence of the optimized geometries was verified by frequency calculations. The absence of negative frequencies characterizes the obtained geometries as energetic minima. The convergence criteria for the frequency calculations were as follows: maximum force 0.00045; RMS force 0.00030; maximum displacement 0.0018; RMS displacement 0.0012. The region on the energy hypersurface was commonly very flat for the present systems, so sometimes the formal convergences of single parameters with minor deviations from the above criteria was not tightly adhered to. Structures showing neglectable predicted changes in energy (<5 × 10^−8^ Hartree) were accepted as converged structures if no imaginary frequencies were obtained.

Further analysis of the obtained wave functions was performed using MultiWFN [156]. For further details on specific aspects, see atomic dipole moment corrected Hirshfeld (ADCH) charges [157] or fuzzy bond order [158,159].

## 4. Conclusions

The {Tc^I^(NO)(Cp)(PPh_3_)}^+^ unit is shown to be a ‘core structure’ for the synthesis of an increasing number of novel technetium(I) complexes. New representatives can readily be prepared by ligand exchange procedures starting from the chlorido compound [Tc(NO)(Cp)(PPh_3_)Cl] or the cationic acetonitrile complex [Tc(NO)(Cp)(PPh_3_)(NCCH_3_)]^+^. Incoming ligands were recruited from anionic or neutral compounds with donor atoms ligands coming from ‘group 4’ to ‘group 7’ of the Periodic Table, including organometallic compounds. ^99^Tc NMR spectroscopy is a helpful tool for the monitoring of corresponding ligand exchange reactions on [Tc^I^(NO)(Cp)(PPh_3_)(L)]^0,+^ complexes since the chemical shifts of the individual compounds are spread over a range of more than 2000 ppm, strictly depending on the nature of the ligand L. The products are stable in air and water, which recommends this class of technetium compounds for the development of corresponding ^99m^Tc derivatives. Such compounds may become interesting as a platform for novel Tc-based radiopharmaceuticals as soon as a synthetic approach for the nanomolar level of the ^99m^Tc chemistry has been found.

## Figures and Tables

**Table 1:** Selected bond lengths (Å) and angles in the [Tc(NO)(Cp)(PPh_3_)(E=PPh_3_)](PF_6_) (E = O (**2**), S (**3**), Se (**4**)) complexes under study.

	Tc-N10	N10-O10	Tc-E	Tc-P1	E-P2	Tc-N10-O10	Tc-E-P2	N10-Tc-E	N10-Tc-P1
**2**(PF_6_)	1.755(4)	1.184(6)	2.149(3)	2.399(1)	1.519(3)	170.4(4)	133.3(2)	102.4(2)	91.4(2)
**3**(PF_6_)	1.768(2)	1.177(3)	2.424(1)	2.373(1)	2.024(1)	174.0(2)	109.22(3)	98.65(6)	91.45(6)
**4**(PF_6_)	1.757(7)	1.186(8)	2.530(1)	2.381(2)	2.183(2)	174.8(6)	107.36(7)	98.9(2)	91.5(2)

**Table 2:** Selected bond lengths (Å) and angles in the [Tc(NO)(Cp)(PPh_3_)(py)]^+^ (**6**) and [Tc(NO)(Cp)(PPh_3_)(*S*-thioxane)]^+^ (**7**) cations.

	Tc-N10	N10-O10	Tc-N1/S1	Tc-P1	Tc-N10-O10	N10-Tc-N1/S1	N10-Tc-P1
**6**(PF_6_)	1.762(2)	1.175(3)	2.161(2)	2.3925(7)	169.3(2)	98.10(8)	90.22(6)
**7**(PF_6_)	1.771(2)	1.174/3)	2.4047(6)	2.3805(6)	172.7(2)	93.20(7)	90.03(7)

## Data Availability

Data are contained within the article and Supplementary Materials.

## Supplementary Materials

The following supporting information can be downloaded at: https://www.mdpi.com/article/10.3390/molecules29051114/s1: Table S1: Crystallographic data and data collection parameters; Figure S1. Ellipsoid representation of [Tc(NO)(Cp)(PPh_3_)(OPPh_3_)](PF_6_) × CH_2_Cl_2_ including the positional disorder in the PF_6_^−^ counter ion. The thermal ellipsoids are set at a 50% probability level. Hydrogen atoms are omitted for clarity; Table S2. Selected bond lengths (Å) and angles (°) in the [Tc(NO)(Cp)(PPh_3_)(OPPh_3_)]^+^ cation; Figure S2. Ellipsoid representation of [Tc(NO)(Cp)(PPh_3_)(SPPh_3_)](PF_6_). The thermal ellipsoids are set at a 50% probability level. Hydrogen atoms are omitted for clarity; Table S3. Selected bond lengths (Å) and angles (°) in the [Tc(NO)(Cp)(PPh_3_)(SPPh_3_)]^+^ cation; Figure S3. Ellipsoid representation of [Tc(NO)(Cp)(PPh_3_)(SePPh_3_)](PF_6_) including the positional disorder in the PF_6_^−^ counter ion. The thermal ellipsoids are set at a 50% probability level. Hydrogen atoms are omitted for clarity; Table S4. Selected bond lengths (Å) and angles (°) in the [Tc(NO)(Cp)(PPh_3_)(SePPh_3_)]^+^ cation; Figure S4. (a) Ellipsoid representation of [Tc(NO)(Cp)(PPh_3_)(NCCH_3_)](BF_4_) including the positional disorder in the BF_4_^−^ counter ion. The thermal ellipsoids are set at a 50% probability level. (b) Ellipsoid representation of [Tc(NO)(Cp)(PPh_3_)(NCCH_3_)](PF_6_) including the positional disorder in the PF_6_^−^ counter ion. The thermal ellipsoids are set at a 50% probability level. Hydrogen atoms are omitted for clarity. The refinement converged at an unsatisfactory R_1_ value of 0.1625, for which reason the structural data should not been deposited with the CCDC database; Table S5. Selected bond lengths (Å) and angles (°) in the [Tc(NO)(Cp)(PPh_3_)(NCCH_3_)]^+^ cations in the BF_4_^−^ and PF_6_^−^ salts; Figure S5. Ellipsoid representation of [Tc(NO)(Cp)(PPh_3_)(py)](PF_6_). Including the positional disorder in the PF_6_^−^ counter ion. The thermal ellipsoids are set at a 50% probability level. Hydrogen atoms are omitted for clarity; Table S6. Selected bond lengths (Å) and angles (°) in the [Tc(NO)(Cp)(PPh_3_)(py)]^+^ cation; Figure S6. Ellipsoid representation of [Tc(NO)(Cp)(PPh_3_)(thioxane)](PF_6_). Including the positional disorder of the S(CH_2_)_2_ unit of the thioxane ligand. The thermal ellipsoids are set at a 50% probability level. Hydrogen atoms are omitted for clarity; Table S7. Selected bond lengths (Å) and angles (°) in the [Tc(NO)(Cp)(PPh_3_)(thioxane)]^+^ cation; Figure S7. Ellipsoid representation of [Tc(NO)(MeCp)(PPh_3_)(py)](PF_6_). The thermal ellipsoids are set at a 50% probability level. Hydrogen atoms are omitted for clarity; Table S8. Selected bond lengths (Å) and angles (°) in the [Tc(NO)(MeCp)(PPh_3_)(py)]^+^ cation.; Figure S8: IR (KBr) spectrum of [Tc(NO)(Cp)(PPh_3_)(OPPh_3_)](PF_6_); Figure S9: ^1^H NMR spectrum of [Tc(NO)(Cp)(PPh_3_)(OPPh_3_)](PF_6_) in CDCl_3_ (* traces of potentially formed *o*-OPPh_3_CH_2_Cl or similar decomposition products; ** OPPh_3_ impurity). Identified impurities and solvents are annotated; Figure S10: ^31^P{^1^H} NMR spectrum of [Tc(NO)(Cp)(PPh_3_)(OPPh_3_)](PF_6_) in CDCl_3_ (*potentially formed *o*-OPPh_3_CH_2_Cl or similar decomposition products). Identified impurities are annotated. An uncommonly large exponential apodization function for ^31^P NMR was applied (100 Hz) after truncation of the FID at 30k points and zero-filling to the original 256k points to enable an interpretation of the very broad resonance for the coordinated PPh_3_ ligand; Figure S11: ^99^Tc NMR spectrum of [Tc(NO)(Cp)(PPh_3_)(OPPh_3_)](PF_6_) in CDCl_3_; Figure S12: ^19^F NMR spectrum of [Tc(NO)(Cp)(PPh_3_)(OPPh_3_)](PF_6_) in CDCl_3_; Figure S13: IR (KBr) spectrum of [Tc(NO)(Cp)(PPh_3_)(SPPh_3_)](PF_6_); Figure S14: ^1^H NMR spectrum of [Tc(NO)(Cp)(PPh_3_)(SPPh_3_)](PF_6_) in CD_2_Cl_2_. Identified solvents are annotated; Figure S15: ^31^P{^1^H} NMR spectrum of [Tc(NO)(Cp)(PPh_3_)(SPPh_3_)](PF_6_) in CD_2_Cl_2_; Figure S16: ^99^Tc NMR spectrum of [Tc(NO)(Cp)(PPh_3_)(SPPh_3_)](PF_6_) in CD_2_Cl_2_; Figure S17: ^19^F NMR spectrum of [Tc(NO)(Cp)(PPh_3_)(SPPh_3_)](PF_6_) in CD_2_Cl_2_; Figure S18: IR (KBr) spectrum of [Tc(NO)(Cp)(PPh_3_)(SePPh_3_)](PF_6_); Figure S19: ^1^H NMR spectrum of [Tc(NO)(Cp)(PPh_3_)(SePPh_3_)](PF_6_) in CD_2_Cl_2_. Identified solvent impurities are annotated; Figure S20: ^31^P{^1^H} NMR spectrum of [Tc(NO)(Cp)(PPh_3_)(SePPh_3_)](PF_6_) in CD_2_Cl_2_. Identified impurities are annotated; Figure S21: ^99^Tc NMR spectrum of [Tc(NO)(Cp)(PPh_3_)(SePPh_3_)](PF_6_) in CD_2_Cl_2_; Figure S22: ^19^F NMR spectrum of [Tc(NO)(Cp)(PPh_3_)(SePPh_3_)](PF_6_) in CD_2_Cl_2_; Figure S23: IR (KBr) spectrum of [Tc(NO)(Cp)(PPh_3_)(NCCH_3_)](PF_6_); Figure S24: ^1^H NMR spectrum of [Tc(NO)(Cp)(PPh_3_)(NCCH_3_)](BF_4_) in CD_2_Cl_2_. Identified impurities and solvents are annotated; Figure S25: ^31^P{^1^H} NMR spectrum of [Tc(NO)(Cp)(PPh_3_)(NCCH_3_)](BF_4_) in CD_2_Cl_2_; Figure S26: ^99^Tc NMR spectrum of [Tc(NO)(Cp)(PPh_3_)(NCCH_3_)](BF_4_) in CD_2_Cl_2_; Figure S27: ^15^N NMR spectrum of [Tc(NO)(Cp)(PPh_3_)(^15^N-NCCH_3_)](BF_4_) in acetone-d_6_; Figure S28: ^19^F NMR spectrum of [Tc(NO)(Cp)(PPh_3_)(NCCH_3_)](BF_4_) in CD_2_Cl_2_; Figure S29: ^15^N NMR spectrum of a 1:1 reaction mixture of [Tc(NO)(Cp)(PPh_3_)(^15^N-NCCH_3_)](BF_4_) and acetonitrile with natural isotopic abundance at room temperature in acetone-d_6_; Figure S30: ^15^N NMR spectra of a 1:1 reaction mixture of [Tc(NO)(Cp)(PPh_3_)(^15^N-NCCH_3_)](BF_4_) and acetonitrile with natural isotopic abundance in acetone-d_6_ at various temperatures; Figure S31: IR (KBr) spectrum of [Tc(NO)(Cp)(PPh_3_)(thioxane)](BF_4_); Figure S32: ^1^H NMR spectrum of [Tc(NO)(Cp)(PPh_3_)(thioxane)](BF_4_) in CD_2_Cl_2_. Identified impurities and solvents are annotated; Figure S33: ^31^P{^1^H} NMR spectrum of [Tc(NO)(Cp)(PPh_3_)(thioxane)](BF_4_) in CD_2_Cl_2_; Figure S34: ^99^Tc NMR spectrum of [Tc(NO)(Cp)(PPh_3_)(thioxane)](BF_4_) in CD_2_Cl_2_; Figure S35: ^19^F NMR spectrum of [Tc(NO)(Cp)(PPh_3_)(thioxane)](BF_4_) in CD_2_Cl_2_; Figure S36: IR (KBr) spectrum of [Tc(NO)(Cp)(PPh_3_)(py)](PF_6_); Figure S37: ^1^H NMR spectrum of [Tc(NO)(Cp)(PPh_3_)(py)](PF_6_) in CDCl_3_. Identified solvents are annotated; Figure S38: ^31^P{^1^H} NMR spectrum of [Tc(NO)(Cp)(PPh_3_)(py)](PF_6_) in CDCl_3_; Figure S39: ^99^Tc NMR spectrum of [Tc(NO)(Cp)(PPh_3_)(py)](PF_6_) in CDCl_3_; Figure S40: ^19^F NMR spectrum of [Tc(NO)(Cp)(PPh_3_)(py)](PF_6_) in CDCl_3_; Figure S41: IR (KBr) spectrum of [Tc(NO)(Cp^Me^)(PPh_3_)Cl]; Figure S42: ^1^H NMR spectrum of [Tc(NO)(Cp^Me^)(PPh_3_)Cl] in CDCl_3_. Identified impurities and solvents are annotated; Figure S43: ^31^P{^1^H} NMR spectrum of [Tc(NO)(Cp^Me^)(PPh_3_)Cl] in CDCl_3_. The observed resonance is ambiguously assigned to the bound PPh_3_ ligand as no other (not even a very broad one) was observed; the narrow resonance would be in accordance with the narrow resonance observed for the pyridine derivative but should not be overvalued as the real resonance could be broad enough to vanish leaving only some OPPh_3_ impurities resonance; Figure S44: ^99^Tc NMR spectrum of [Tc(NO)(Cp^Me^)(PPh_3_)Cl] in CDCl_3_; Figure S45: IR (KBr) spectrum of [Tc(NO)(Cp^Me^)(PPh_3_)(py)](PF_6_); Figure S46: ^1^H NMR spectrum of [Tc(NO)(Cp^Me^)(PPh_3_)(py)](PF_6_) in CDCl_3_. Identified impurities and solvents are annotated; Figure S47: ^31^P{^1^H} NMR spectrum of [Tc(NO)(Cp^Me^)(PPh_3_)(py)](PF_6_) in CDCl_3_. Identified impurities are annotated. An uncommonly large exponential apodization function for ^31^P NMR was applied (20 Hz) after truncation of the FID at 5k points and zero-filling to the original 256k points to improve the interpretability of the somewhat broader resonance of the coordinated PPh_3_ ligand; Figure S48: ^99^Tc NMR spectrum of [Tc(NO)(Cp^Me^)(PPh_3_)(py)](PF_6_) in CDCl_3_; Figure S49: IR (KBr) spectrum of [Tc(NO)(Cp^COOMe^)(PPh_3_)Cl]; Figure S50: ^1^H NMR spectrum of [Tc(NO)(CpCOOMe)(PPh_3_)Cl] in CDCl_3_. Identified impurities and solvents are annotated; Figure S51: ^31^P{^1^H} NMR spectrum of [Tc(NO)(CpCOOMe)(PPh_3_)Cl] in CDCl_3_. Identified are annotated. An uncommonly large exponential apodization function for ^31^P NMR was applied (100 Hz) after truncation of the FID at 5k points and zero-filling to the original 256k points to improve the interpretability of the very broad resonance of the coordinated PPh_3_ ligand; Figure S52: ^99^Tc NMR spectrum of [Tc(NO)(CpCOOMe)(PPh_3_)Cl] in CDCl_3_; Figure S53: ^19^F NMR spectrum of a reaction mixture containing PF_6_^−^ anions together with H_2_O, MeOH and metal ions, showing the gradual degradation of hexafluorophosphate under formation of oxyfluorides and HF; Table S9. ^99^Tc NMR chemical shifts and line widths of [Tc(NO)(Cp^R^)(PPh_3_)(L)]^0,+^ complexes; Figure S54: Gas-phase optimized structure of TcO_4_^−^; Figure S55: Gas-phase optimized structure of [Tc(NO)(Cp)(PPh_3_)(*S*-thioxane)]^+^; Figure S56: Gas-phase optimized structure of [Tc(NO)(Cp)(PPh_3_)(*O*-thioxane)]^+^; Figure S57: Gas-phase optimized structure of [Tc(NO)(Cp)(PPh_3_)(OPPh_3_)]^+^; Figure S58: Gas-phase optimized structure of [Tc(NO)(Cp)(PPh_3_)(SPPh_3_)]^+^; Figure S59: Gas-phase optimized structure of [Tc(NO)(Cp)(PPh_3_)(SePPh_3_)]^+^; Figure S60: Gas-phase optimized structure of OPPh_3_; Figure S61: Gas-phase optimized structure of SPPh_3_; Figure S62: Gas-phase optimized structure of SePPh_3_; Figure S63: Electron localization function plot for the gas-phase optimized structure of free A) OPPh_3_, B) SPPh_3_ and C) SePPh_3_. Color scale: blue = 1, green = 0.5 and red = 0; Table S10: Comparison of the calculated adapted Hirshfeld (ADCH) charges, fuzzy bond orders and experimental NMR chemical shifts in the phosphine chalcogenides and their technetium(I) complexes; Figure S64: Correlation between ADCH charges and ^99^Tc chemical shift of the complexes. For the ADCH at technetium and the phosphorus atom in the phosphine chalcogenide ligand, trend lines are provided as they correlate linearly; Figure S65: Correlation between ADCH charges and ^99^Tc chemical shift of the complexes. For the ADCH at technetium and the phosphorus atom in the phosphine chalcogenide ligand, trend lines are provided as they correlate linearly; Figure S66: Theoretical IR spectrum (intensity cut-off for peak-labels: 2%) of the gas-phase optimized structure of TcO_4_^−^; Figure S67: Theoretical IR spectrum (intensity cut-off for peak-labels: 2%) of the gas-phase optimized structure of [Tc(NO)(Cp)(PPh_3_)(*S*-thioxane)]^+^; Figure S68: Theoretical IR spectrum (intensity cut-off for peak-labels: 2%) of the gas-phase optimized structure of [Tc(NO)(Cp)(PPh_3_)(*O*-thioxane)]^+^; Figure S69: Theoretical IR spectrum (intensity cut-off for peak-labels: 2%) of the gas-phase optimized structure of [Tc(NO)(Cp)(PPh_3_)(OPPh_3_)]^+^; Figure S70: Theoretical IR spectrum (intensity cut-off for peak-labels: 2%) of the gas-phase optimized structure of [Tc(NO)(Cp)(PPh_3_)(SPPh_3_)]^+^; Figure S71: Theoretical IR spectrum (intensity cut-off for peak-labels: 20%) of the gas-phase optimized structure of [Tc(NO)(Cp)(PPh_3_)(SePPh_3_)]^+^; Figure S72: Theoretical IR spectrum (intensity cut-off for peak-labels: 20%) of the gas-phase optimized structure of OPPh_3_; Figure S73: Theoretical IR spectrum (intensity cut-off for peak-labels: 20%) of the gas-phase optimized structure of SPPh_3_; Figure S74: Theoretical IR spectrum (intensity cut-off for peak-labels: 20%) of the gas-phase optimized structure of SePPh_3_; Figure S75: Overlays of the computed gas-phase structures of a) [Tc(NO)(Cp)(PPh_3_)(OPPh_3_)]^+^, b) [Tc(NO)(Cp)(PPh_3_)(SPPh_3_)]^+^ and c) [Tc(NO)(Cp)(PPh_3_)(SePPh_3_)]^+^ with the corresponding structures derived from the X-ray diffraction data; Figure S76: Overlay of the computed gas-phase structures of [Tc(NO)(Cp)(PPh_3_)(thioxane)]^+^ with the corresponding structures derived from the X-ray diffraction data.; Figure S77: Optimized structure of [Tc(NO)(Cp)(PPh_3_)(*S*-thioxane)]^+^ in THF solution; Figure S78: Optimized structure of [Tc(NO)(Cp)(PPh_3_)(*O*-thioxane)]^+^ in THF solution; Figure S79: Optimized structure of [Tc(NO)(Cp)(PPh_3_)]^+^ in THF solution; Figure S80: Optimized structure of [Tc(NO)(Cp)(PPh_3_)(NCCH_3_)]^+^ in THF solution; Figure S81: Optimized structure of [Tc(NO)(Cp)(PPh_3_)(pyridine)]^+^ in THF solution; Figure S82: Optimized structure of [Tc(NO)(Cp)(PPh_3_)Cl] in THF solution; Figure S83: Optimized structure of [Tc(NO)(Cp)(PPh_3_){µ-ClTc(NO)(Cp)(PPh_3_)}]^+^ in THF solution; Figure S84: Optimized structure of pyridine in THF solution; Figure S85: Optimized structure of acetonitrile in THF solution; Figure S86: Optimized structure of thioxane in THF solution; Figure S87: Overlays of the computed structures of a) [Tc(NO)(Cp)(thioxane)]^+^, b) [Tc(NO)(Cp)(NCCH_3_)]^+^, c) [Tc(NO)(Cp)(py)]^+^ and d) [{Tc(NO)(Cp)(PPh_3_)}_2_Cl]^+^ in THF with the corresponding structure derived from the X-ray diffraction data; Table S11: Thermochemistry of *S* versus *O* coordination in thioxane. *k* = 8.314462618 × 10^−3^ kJ/(mol∙K); energy conversion: 1 [a.u.] = 2625.50 [kJ/mol]; Table S12: Thermochemistry (ΔG) for the dissociation of ligands from [Tc(NO)(Cp)(PPh_3_)]^+^ with some ligands of this study in THF solution. Energy conversion: 1 [a.u.] = 2625.50 [kJ/mol]; Table S13: Experimental *versus* some preliminary DFT-based chemical shifts of technetium compounds; Figure S88: Linear correlation for the theoretical and experimental chemical shifts shown in Table S12. Note that while these compounds correlate fairly well, the other complexes of this study are much more difficult to model with regard to their theoretical ^99^Tc NMR properties due to complex solvent effects or potential dynamic behavior and a dedicated manuscript for the theoretical description of ^99^Tc NMR chemical shifts is planned for the future to address these issues in detail; Figure S89: Theoretical IR spectrum (intensity cut-off for peak-labels: 2%) of [Tc(NO)(Cp)(PPh_3_)(*S*-thioxane)]^+^ in THF solution; Figure S90: Theoretical IR spectrum (intensity cut-off for peak-labels: 2%) of [Tc(NO)(Cp)(PPh_3_)(*O*-thioxane)]^+^ in THF solution; Figure S91: Theoretical IR spectrum (intensity cut-off for peak-labels: 2%) of [Tc(NO)(Cp)(PPh_3_)]^+^ in THF solution; Figure S92: Theoretical IR spectrum (intensity cut-off for peak-labels: 2%) of [Tc(NO)(Cp)(PPh_3_)(NCCH_3_)]^+^ in THF solution; Figure S93: Theoretical IR spectrum (intensity cut-off for peak-labels: 2%) of [Tc(NO)(Cp)(PPh_3_)(pyridine)]^+^ in THF solution; Figure S94: Theoretical IR spectrum (intensity cut-off for peak-labels: 2%) of [Tc(NO)(Cp)(PPh_3_)Cl] in THF solution; Figure S95: Theoretical IR spectrum (intensity cut-off for peak-labels: 2%) of [Tc(NO)(Cp)(PPh_3_){µ-ClTc(NO)(Cp)(PPh_3_)}]^+^ in THF solution; Figure S96: Theoretical IR spectrum (intensity cut-off for peak-labels: 20%) of pyridine in THF solution; Figure S97: Theoretical IR spectrum (intensity cut-off for peak-labels: 20%) of acetonitrile in THF solution; Figure S98: Theoretical IR spectrum (intensity cut-off for peak-labels: 20%) of thioxane in THF solution.
