# Supplementary material for: [Tc(NO)(Cp)(PPh3)Cl] and [Tc(NO)(Cp)(PPh3)(NCCH3)](PF6), and Their Reactions with Pyridine and Chalcogen Donors
Source: Molecules. 2024 Mar 1;29(5):1114. doi: 10.3390/molecules29051114 (PMC10935158; doi:10.3390/molecules29051114)
Supplement: Supplementary file 1 [file molecules-29-01114-s001.zip › TcNO_Cp_Supplementary Material_R1.pdf]

Supplementary Materials to the paper entitled:

[Tc(NO)(Cp)(PPh_3)Cl] and [Tc(NO)(Cp)(PPh_3)($NCCH_3$)](PF_6), and Their Reactions with Pyridine and Chalcogen Donors

Moritz Johannes Ernst,[1] Abdullah Adulkader,[1] Adelheid Hagenbach,[1] Guilhem Claude,[1] Maximilian Roca Jungfer,[2*] and Ulrich Abram[1*]

[1] Freie Universität Berlin, Institute of Chemistry and Biochemistry, Fabeckstr. 34/36, 14195 Berlin, Germany.

[2] Ruprecht-Karls Universität Heidelberg, Im Neuenheimer Feld 271, D-69120 Heidelberg, Germany.

Table of content

Crystallographic data 8

Table S1: Crystallographic data and data collection parameters 8

Table S1: Crystallographic data and data collection parameters (continued) 9

Table S1: Crystallographic data and data collection parameters (continued) 10

Table S1: Crystallographic data and data collection parameters (continued) 11

Figure S1. Ellipsoid representation of $[Tc(NO)(Cp)(PPh_3)(OPPh_3)](PF_6)$ x CH_2Cl_2 including the positional disorder in the PF_6^- counter ion. The thermal ellipsoids are set at a 50% probability level. Hydrogen atoms are omitted for clarity. 12

Table S2. Selected bond lengths (Å) and angles (°) in the $[Tc(NO)(Cp)(PPh_3)(OPPh_3)]^+$ cation. 12

Figure S2. Ellipsoid representation of $[Tc(NO)(Cp)(PPh_3)(SPPh_3)](PF_6)$. The thermal ellipsoids are set at a 50% probability level. Hydrogen atoms are omitted for clarity. 13

Table S3. Selected bond lengths (Å) and angles (°) in the $[Tc(NO)(Cp)(PPh_3)(SPPh_3)]^+$ cation. 13

Figure S3. Ellipsoid representation of $[Tc(NO)(Cp)(PPh_3)(SePPh_3)](PF_6)$ including the positional disorder in the PF_6^- counter ion. The thermal ellipsoids are set at a 50% probability level. Hydrogen atoms are omitted for clarity. 14

Table S4. Selected bond lengths (Å) and angles (°) in the $[Tc(NO)(Cp)(PPh_3)(SePPh_3)]^+$ cation. 14

Figure S4. (a) Ellipsoid representation of $[Tc(NO)(Cp)(PPh_3)(NCCH_3)](BF_4)$ including the positional disorder in the BF_4^- counter ion. The thermal ellipsoids are set at a 50% probability level. (b) Ellipsoid representation of $[Tc(NO)(Cp)(PPh_3)(NCCH_3)](PF_6)$ including the positional disorder in the PF_6^- counter ion. The thermal ellipsoids are set at a 50% probability level. Hydrogen atoms are omitted for clarity. The refinement converged at an unsatisfactory R_1 value of 0.1625, for which reason the structural data should not been deposited with the CCDC data base. 15

Table S5. Selected bond lengths (Å) and angles (°) in the $[Tc(NO)(Cp)(PPh_3)(NCCH_3)]^+$ cations in the BF_4^- and PF_6^- salts. 15

Figure S5. Ellipsoid representation of $[Tc(NO)(Cp)(PPh_3)(py)](PF_6)$. including the positional disorder in the PF_6^- counter ion. The thermal ellipsoids are set at a 50% probability level. Hydrogen atoms are omitted for clarity. 16

Table S6. Selected bond lengths (Å) and angles (°) in the $[Tc(NO)(Cp)(PPh_3)(py)]^+$ cation. 16

Figure S6. Ellipsoid representation of $[Tc(NO)(Cp)(PPh_3)(thioxane)](PF_6)$. including the positional disorder of the $S(CH_2)_2$ unit of the thioxane ligand. The thermal ellipsoids are set at a 50% probability level. Hydrogen atoms are omitted for clarity. 17

Table S7. Selected bond lengths (Å) and angles (°) in the $[Tc(NO)(Cp)(PPh_3)(thioxane)]^+$ cation. 17

Figure S7. Ellipsoid representation of $[Tc(NO)(MeCp)(PPh_3)(py)](PF_6)$. The thermal ellipsoids are set at a 50% probability level. Hydrogen atoms are omitted for clarity. 18

Table S8. Selected bond lengths (Å) and angles (°) in the $[Tc(NO)(MeCp)(PPh_3)(py)]^+$ cation. 18

Spectroscopic data 19

Figure S8: IR (KBr) spectrum of $[Tc(NO)(Cp)(PPh_3)(OPPh_3)](PF_6)$. 19

Figure S9: ^1H NMR spectrum of $[Tc(NO)(Cp)(PPh_3)(OPPh_3)](PF_6)$ in $CDCl_3$ (* traces of potentially formed *o*-$OPPh_3CH_2Cl$ or similar decomposition products; ** $OPPh_3$ impurity) Identified impurities and solvents are annotated. 19

Figure S10: $^{31}P\{^1H\}$ NMR spectrum of $[Tc(NO)(Cp)(PPh_3)(OPPh_3)](PF_6)$ in $CDCl_3$ (* potentially formed *o*-$OPPh_3CH_2Cl$ or similar decomposition products). Identified impurities are annotated. An uncommonly large exponential apodization function for ^{31}P NMR was applied (100 Hz) after truncation of the FID at 30k points and zero-filling to the original 256k points to enable an interpretation of the very broad resonance for the coordinated PPh_3 ligand. 20

Figure S11: ^{99}Tc NMR spectrum of $[Tc(NO)(Cp)(PPh_3)(OPPh_3)](PF_6)$ in $CDCl_3$. 20

Figure S12: ^{19}F NMR spectrum of $[Tc(NO)(Cp)(PPh_3)(OPPh_3)](PF_6)$ in $CDCl_3$. 21

Figure S13: IR (KBr) spectrum of $[Tc(NO)(Cp)(PPh_3)(SPPh_3)](PF_6)$. 21

Figure S14: ^1H NMR spectrum of $[Tc(NO)(Cp)(PPh_3)(SPPh_3)](PF_6)$ in CD_2Cl_2. Identified solvents are annotated. 22

Figure S15: $^{31}P\{^1H\}$ NMR spectrum of $[Tc(NO)(Cp)(PPh_3)(SPPh_3)](PF_6)$ in CD_2Cl_2. 22

Figure S16: ^{99}Tc NMR spectrum of $[Tc(NO)(Cp)(PPh_3)(SPPh_3)](PF_6)$ in CD_2Cl_2. 23

Figure S17: ^{19}F NMR spectrum of $[Tc(NO)(Cp)(PPh_3)(SPPh_3)](PF_6)$ in CD_2Cl_2. 23

Figure S18: IR (KBr) spectrum of $[Tc(NO)(Cp)(PPh_3)(SePPh_3)](PF_6)$. 24

Figure S19: ^1H NMR spectrum of $[Tc(NO)(Cp)(PPh_3)(SePPh_3)](PF_6)$ in CD_2Cl_2. Identified solvent impurities are annotated. 24

Figure S20: $^{31}P\{^1H\}$ NMR spectrum of $[Tc(NO)(Cp)(PPh_3)(SePPh_3)](PF_6)$ in CD_2Cl_2. Identified impurities are annotated. 25

Figure S21: ^{99}Tc NMR spectrum of $[Tc(NO)(Cp)(PPh_3)(SePPh_3)](PF_6)$ in CD_2Cl_2. 25

Figure S22: ^{19}F NMR spectrum of $[Tc(NO)(Cp)(PPh_3)(SePPh_3)](PF_6)$ in CD_2Cl_2. 26

Figure S23: IR (KBr) spectrum of $[Tc(NO)(Cp)(PPh_3)(NCCH_3)](PF_6)$. 26

Figure S24: ^1H NMR spectrum of $[Tc(NO)(Cp)(PPh_3)(NCCH_3)](BF_4)$ in CD_2Cl_2. Identified impurities and solvents are annotated. 27

Figure S25: $^{31}P\{^1H\}$ NMR spectrum of $[Tc(NO)(Cp)(PPh_3)(NCCH_3)](BF_4)$ in CD_2Cl_2. 27

Figure S26: ^{99}Tc NMR spectrum of $[Tc(NO)(Cp)(PPh_3)(NCCH_3)](BF_4)$ in CD_2Cl_2. 28

Figure S27: ^{15}N NMR spectrum of [Tc(NO)(Cp)(PPh_3)(^{15}N-$NCCH_3$)](BF_4) in acetone-d_6. 28

Figure S28: ^{19}F NMR spectrum of [Tc(NO)(Cp)(PPh_3)($NCCH_3$)](BF_4) in CD_2Cl_2. 29

Figure S29: ^{15}N NMR spectrum of a 1:1 reaction mixture of [Tc(NO)(Cp)(PPh_3)(^{15}N-$NCCH_3$)](BF_4) and acetonitrile with natural isotopic abundance at room temperature in acetone-d_6. 29

Figure S30: ^{15}N NMR spectra of a 1:1 reaction mixture of [Tc(NO)(Cp)(PPh_3)(^{15}N-$NCCH_3$)](BF_4) and acetonitrile with natural isotopic abundance in acetone-d_6 at various temperatures. 30

Figure S31: IR (KBr) spectrum of [Tc(NO)(Cp)(PPh_3)(thioxane)](BF_4). 30

Figure S32: ^{1}H NMR spectrum of [Tc(NO)(Cp)(PPh_3)(thioxane)](BF_4) in CD_2Cl_2. Identified impurities and solvents are annotated. 31

Figure S33: ^{31}P{^{1}H} NMR spectrum of [Tc(NO)(Cp)(PPh_3)(thioxane)](BF_4) in CD_2Cl_2. 31

Figure S34: ^{99}Tc NMR spectrum of [Tc(NO)(Cp)(PPh_3)(thioxane)](BF_4) in CD_2Cl_2. 32

Figure S35: ^{19}F NMR spectrum of [Tc(NO)(Cp)(PPh_3)(thioxane)](BF_4) in CD_2Cl_2. 32

Figure S36: IR (KBr) spectrum of [Tc(NO)(Cp)(PPh_3)(py)](PF_6). 33

Figure S37: ^{1}H NMR spectrum of [Tc(NO)(Cp)(PPh_3)(py)](PF_6) in $CDCl_3$. Identified solvents are annotated. 33

Figure S38: ^{31}P{^{1}H} NMR spectrum of [Tc(NO)(Cp)(PPh_3)(py)](PF_6) in $CDCl_3$. 34

Figure S39: ^{99}Tc NMR spectrum of [Tc(NO)(Cp)(PPh_3)(py)](PF_6) in $CDCl_3$. 34

Figure S40: ^{19}F NMR spectrum of [Tc(NO)(Cp)(PPh_3)(py)](PF_6) in $CDCl_3$. 35

Figure S41: IR (KBr) spectrum of [Tc(NO)(Cp^{Me})(PPh_3)Cl]. 35

Figure S42: ^{1}H NMR spectrum of [Tc(NO)(Cp^{Me})(PPh_3)Cl] in $CDCl_3$. Identified impurities and solvents are annotated. 36

Figure S43: ^{31}P{^{1}H} NMR spectrum of [Tc(NO)(Cp^{Me})(PPh_3)Cl] in $CDCl_3$. The observed resonance is ambiguously assigned to the bound PPh_3 ligand as no other (not even a very broad one) was observed; the narrow resonance would be in accordance with the narrow resonance observed for the pyridine derivative but should not be overvalued as the real resonance could be broad enough to vanish leaving only some $OPPh_3$ impurities resonance. 36

Figure S44: ^{99}Tc NMR spectrum of [Tc(NO)(Cp^{Me})(PPh_3)Cl] in $CDCl_3$. 37

Figure S45: IR (KBr) spectrum of [Tc(NO)(Cp^{Me})(PPh_3)(py)](PF_6). 37

Figure S46: ^{1}H NMR spectrum of [Tc(NO)(Cp^{Me})(PPh_3)(py)](PF_6) in $CDCl_3$. Identified impurities and solvents are annotated. 38

Figure S47: ^{31}P{^{1}H} NMR spectrum of [Tc(NO)(Cp^{Me})(PPh_3)(py)](PF_6) in $CDCl_3$. Identified impurities are annotated. An uncommonly large exponential apodization function for ^{31}P NMR was applied (20 Hz) after truncation of the FID at 5k points and zero-filling to the original 256k points to improve the interpretability of the somewhat broader resonance of the coordinated PPh_3 ligand. 38

Figure S48: ^{99}Tc NMR spectrum of [Tc(NO)(Cp^{Me})(PPh$_3$)(py)](PF$_6$) in CDCl$_3$. 39

Figure S49: IR (KBr) spectrum of [Tc(NO)(Cp^{COOMe})(PPh$_3$)Cl]. 39

Figure S50: ^{1}H NMR spectrum of [Tc(NO)(CpCOOMe)(PPh$_3$)Cl] in CDCl$_3$. Identified impurities and solvents are annotated. 40

Figure S51: ^{31}P{^{1}H} NMR spectrum of [Tc(NO)(CpCOOMe)(PPh$_3$)Cl] in CDCl$_3$. Identified are annotated. An uncommonly large exponential apodization function for ^{31}P NMR was applied (100 Hz) after truncation of the FID at 5k points and zero-filling to the original 256k points to improve the interpretability of the very broad resonance of the coordinated PPh$_3$ ligand 40

Figure S52: ^{99}Tc NMR spectrum of [Tc(NO)(CpCOOMe)(PPh$_3$)Cl] in CDCl$_3$. 41

Figure S53: ^{19}F NMR spectrum of a reaction mixture containing PF_6^- anions together with H_2O, MeOH and metal ions, showing the gradual degradation of hexafluorophosphate under formation of oxyfluorides and HF. 41

Table S9. ^{99}Tc NMR chemical shifts and line widths of [Tc(NO)(Cp^R)(PPh$_3$)(L)]$^{0,+}$ complexes 42

Computational Data 1: Gas-Phase 43

Figure S54: Gas-phase optimized structure of TcO_4^-. 43

Figure S55: Gas-phase optimized structure of [Tc(NO)(Cp)(PPh$_3$)(*S*-thioxane)]$^+$............ 43

Figure S56: Gas-phase optimized structure of [Tc(NO)(Cp)(PPh$_3$)(*O*-thioxane)]$^+$........... 43

Figure S57: Gas-phase optimized structure of [Tc(NO)(Cp)(PPh$_3$)(OPPh$_3$)]$^+$. 44

Figure S58: Gas-phase optimized structure of [Tc(NO)(Cp)(PPh$_3$)(SPPh$_3$)]$^+$. 44

Figure S59: Gas-phase optimized structure of [Tc(NO)(Cp)(PPh$_3$)(SePPh$_3$)]$^+$. 44

Figure S60: Gas-phase optimized structure of OPPh$_3$. 45

Figure S61: Gas-phase optimized structure of SPPh$_3$. 45

Figure S62: Gas-phase optimized structure of SePPh$_3$. 45

Figure S63: Electron localization function plot for the gas-phase optimized structure of free A) OPPh$_3$, B) SPPh$_3$ and C) SePPh$_3$. Color-scale: blue = 1, green = 0.5 and red = 0. ... 46

Table S10: Comparison of the calculated adapted Hirshfeld (ADCH) charges, fuzzy bond orders and experimental NMR chemical shifts in the phosphine chalcogenides and their technetium(I) complexes. 46

Figure S64: Correlation between ADCH charges and ^{99}Tc chemical shift of the complexes. For the ADCH at technetium and the phosphorus atom in the phosphine chalcogenide ligand, trend lines are provided as they correlate linearly.......................... 47

Figure S65: Correlation between ADCH charges and ^{99}Tc chemical shift of the complexes. For the ADCH at technetium and the phosphorus atom in the phosphine chalcogenide ligand, trend lines are provided as they correlate linearly.......................... 47

Figure S66: Theoretical IR spectrum (intensity cut-off for peak-labels: 2 km/mol) of the gas-phase optimized structure of TcO_4^-. 48

Figure S67: Theoretical IR spectrum (intensity cut-off for peak-labels: 2 km/mol) of the gas-phase optimized structure of $[Tc(NO)(Cp)(PPh_3)(S\text{-thioxane})]^+$. 48

Figure S68: Theoretical IR spectrum (intensity cut-off for peak-labels: 2 km/mol) of the gas-phase optimized structure of $[Tc(NO)(Cp)(PPh_3)(O\text{-thioxane})]^+$. 49

Figure S69: Theoretical IR spectrum (intensity cut-off for peak-labels: 2 km/mol) of the gas-phase optimized structure of $[Tc(NO)(Cp)(PPh_3)(OPPh_3)]^+$. 49

Figure S70: Theoretical IR spectrum (intensity cut-off for peak-labels: 2 km/mol) of the gas-phase optimized structure of $[Tc(NO)(Cp)(PPh_3)(SPPh_3)]^+$. 50

Figure S71: Theoretical IR spectrum (intensity cut-off for peak-labels: 20 km/mol) of the gas-phase optimized structure of $[Tc(NO)(Cp)(PPh_3)(SePPh_3)]^+$. 50

Figure S72: Theoretical IR spectrum (intensity cut-off for peak-labels: 20 km/mol) of the gas-phase optimized structure of $OPPh_3$. 51

Figure S73: Theoretical IR spectrum (intensity cut-off for peak-labels: 20 km/mol) of the gas-phase optimized structure of $SPPh_3$. 51

Figure S74: Theoretical IR spectrum (intensity cut-off for peak-labels: 20 km/mol) of the gas-phase optimized structure of $SePPh_3$. 52

Figure S75: Overlays of the computed gas-phase structures of a) $[Tc(NO)(Cp)(PPh_3)(OPPh_3)]^+$, b) $[Tc(NO)(Cp)(PPh_3)(SPPh_3)]^+$ and c) $[Tc(NO)(Cp)(PPh_3)(SePPh_3)]^+$ with the corresponding structures derived from the X-ray diffraction data. 52

Figure S76: Overlay of the computed gas-phase structures of $[Tc(NO)(Cp)(PPh_3)(\text{thioxane})]^+$ with the corresponding structures derived from the X-ray diffraction data. 52

Computational Data 2: Implicit Solvation Model 53

Figure S77: Optimized structure of $[Tc(NO)(Cp)(PPh_3)(S\text{-thioxane})]^+$ in THF solution. 53

Figure S78: Optimized structure of $[Tc(NO)(Cp)(PPh_3)(O\text{-thioxane})]^+$ in THF solution. 53

Figure S79: Optimized structure of $[Tc(NO)(Cp)(PPh_3)]^+$ in THF solution. 54

Figure S80: Optimized structure of $[Tc(NO)(Cp)(PPh_3)(NCCH_3)]^+$ in THF solution. 54

Figure S81: Optimized structure of $[Tc(NO)(Cp)(PPh_3)(\text{pyridine})]^+$ in THF solution. 55

Figure S82: Optimized structure of $[Tc(NO)(Cp)(PPh_3)Cl]$ in THF solution. 55

Figure S83: Optimized structure of $[Tc(NO)(Cp)(PPh_3)\{\mu\text{-}ClTc(NO)(Cp)(PPh_3)\}]^+$ in THF solution. 56

Figure S84: Optimized structure of pyridine in THF solution. 56

Figure S85: Optimized structure of acetonitrile in THF solution. 57

Figure S86: Optimized structure of thioxane in THF solution. 57

Figure S87: Overlays of the computed structures of a) $[Tc(NO)(Cp)(\text{thioxane})]^+$, b) $[Tc(NO)(Cp)(NCCH_3)]^+$, c) $[Tc(NO)(Cp)(py)]^+$ and d) $[\{Tc(NO)(Cp)(PPh_3)\}_2Cl]^+$ in THF with the corresponding structure derived from the X-ray diffraction data. 57

Table S11: Thermochemistry of *S* versus *O* coordination in thioxane. *k* = 8.314462618×10^{-3} kJ/(mol·K); energy conversion: 1 [a.u.] = 2625.50 [kJ/mol]. 58

Table S12: Thermochemistry (ΔG) for the dissociation of ligands from $[Tc(NO)(Cp)(PPh_3)]^+$ with some ligands of this study in THF solution. Energy conversion: 1 [a.u.] = 2625.50 [kJ/mol]. 58

Table S13: Experimental *versus* some preliminary DFT-based chemical shifts of technetium compounds. 58

Figure S88: Linear correlation for the theoretical and experimental chemical shifts shown in Table S12. Note that while these compounds correlate fairly well, the other complexes of this study are much more difficult to model with regard to their theoretical ^{99}Tc NMR properties due to complex solvent effects or potential dynamic behavior and a dedicated manuscript for the theoretical description of ^{99}Tc NMR chemical shifts is planned for the future to address these issues in detail. 59

Figure S89: Theoretical IR spectrum (intensity cut-off for peak-labels: 2 km/mol) of $[Tc(NO)(Cp)(PPh_3)(S\text{-thioxane})]^+$ in THF solution. 59

Figure S90: Theoretical IR spectrum (intensity cut-off for peak-labels: 2 km/mol) of $[Tc(NO)(Cp)(PPh_3)(O\text{-thioxane})]^+$ in THF solution. 60

Figure S91: Theoretical IR spectrum (intensity cut-off for peak-labels: 2 km/mol) of $[Tc(NO)(Cp)(PPh_3)]^+$ in THF solution. 60

Figure S92: Theoretical IR spectrum (intensity cut-off for peak-labels: 2 km/mol) of $[Tc(NO)(Cp)(PPh_3)(NCCH_3)]^+$ in THF solution. 61

Figure S93: Theoretical IR spectrum (intensity cut-off for peak-labels: 2 km/mol) of $[Tc(NO)(Cp)(PPh_3)(pyridine)]^+$ in THF solution. 61

Figure S94: Theoretical IR spectrum (intensity cut-off for peak-labels: 2 km/mol) of $[Tc(NO)(Cp)(PPh_3)Cl]$ in THF solution. 62

Figure S95: Theoretical IR spectrum (intensity cut-off for peak-labels: 2 km/mol) of $[Tc(NO)(Cp)(PPh_3)\{\mu\text{-}ClTc(NO)(Cp)(PPh_3)\}]^+$ in THF solution. 62

Figure S96: Theoretical IR spectrum (intensity cut-off for peak-labels: 20 km/mol) of pyridine in THF solution. 63

Figure S97: Theoretical IR spectrum (intensity cut-off for peak-labels: 20 km/mol) of acetonitrile in THF solution. 63

Figure S98: Theoretical IR spectrum (intensity cut-off for peak-labels: 20 km/mol) of thioxane in THF solution. 64

Crystallographic data

Table S1: Crystallographic data and data collection parameters

	[Tc(NO)(Cp)(PPh_3)(OPh_3)](PF_6) (**2**(PF_6)) · CH_2Cl_2	[Tc(NO)(Cp)(PPh_3)(SPh_3)](PF_6) (**3**(PF_6))
Empirical formula	$C_{42}F_6NO_2P_3TcCl_2H_{37}$	$C_{41}F_6NOP_3TcSH_{35}$
Formula weight	963.53	894.67
Temperature/K	100(2)	100(2)
Crystal system	Monoclinic	Monoclinic
Space group	$P2_1/c$	$P2_1/c$
a/Å	22.075(2)	121.220(1)
b/Å	10.6519(9)	11.1669(5)
c/Å	18.788(2)	17.451(1)
α/°	90	90
β/°	109.277(3)	112.156(2)
γ/°	90	90
Volume/$Å^3$	4170.1(7)	3829.8(3)
Z	4	4
ρ_{calc} / gcm^{-3}	1.535	1.552
μ / mm^{-1}	0.653	0.620
F(000)	1952.0	1816.0
Crystal size / mm^3	0.27 × 0.19 × 0.09	0.18 × 0.16 × 0.08
Radiation	MoKα (λ = 0.71073)	MoKα (λ = 0.71073)
2Θ range for data collection/°	4.46 to 54.412	4.434 to 54.614
Index ranges	-28 ≤ h ≤ 28, -13 ≤ k ≤ 13, -24 ≤ l ≤ 24	-27 ≤ h ≤ 27, -14 ≤ k ≤ 14, -22 ≤ l ≤ 22
Reflections collected	85431	119727
Independent reflections	9259 [R_{int} = 0.0462, R_{sigma} = 0.0252]	8570 [R_{int} = 0.1050, R_{sigma} = 0.0380]
Data/restraints/parameters	9259/0/515	8570/0/627
Goodness-of-fit on F^2	1.191	1.032
Final R indexes [I>=2σ (I)]	R_1 = 0.0690, wR_2 = 0.1453	R_1 = 0.0340, wR_2 = 0.0649
Final R indexes [all data]	R_1 = 0.0775, wR_2 = 0.1490	R_1 = 0.0551, wR_2 = 0.0715
Largest diff. peak/hole / e $Å^{-3}$	2.38/-1.90	0.54/-0.32
Diffractometer	Bruker APEX	Bruker APEX
CCDC access code	2329526	2329527

Table S1: Crystallographic data and data collection parameters (continued)

	$[Tc(NO)(Cp)(PPh_3)(SePh_3)](PF_6)$ (**4**(PF_6))	$[Tc(NO)(Cp)(PPh_3)(NCCH_3)](BF_4)$ (**5**(BF_4))
Empirical formula	$C_{41}F_6NOP_3SeTcH_{35}$	$C_{25}H_{23}BF_6N_2OPTc$
Formula weight	941.57	583.23
Temperature/K	293(2)	197(2)
Crystal system	Monoclinic	Monoclinic
Space group	$P2_1/c$	Cc
a/Å	21.567(2)	13.541(3)
b/Å	11.2351(9)	14.059(4)
c/Å	17.759(1)	13.782(4)
α/°	90	90
β/°	112.228(5)	98.07(2)
γ/°	90	90
Volume/Å^3	3983.4(5)	2597.8(12)
Z	4	4
ρ_{calc} / gcm^{-3}	1.570	1.491
μ / mm^{-1}	1.457	0.665
F(000)	1888.0	1176.0
Crystal size / mm^3	0.18 × 0.14 × 0.08	0.25 × 0.22 × 0.20
Radiation	MoKα (λ = 0.71073)	MoKα (λ = 0.71073)
2Θ range for data collection/°	6.604 to 57.99	6.52 to 58.492
Index ranges	-29 ≤ h ≤ 29, -15 ≤ k ≤ 13, -24 ≤ l ≤ 24	-16 ≤ h ≤ 18, -19 ≤ k ≤ 19, -18 ≤ l ≤ 18
Reflections collected	45560	10412
Independent reflections	10582 [R_{int} = 0.2471, R_{sigma} = 0.2491]	6165 [R_{int} = 0.0353, R_{sigma} = 0.0534]
Data/restraints/parameters	10582/21/479	6165/2/353
Goodness-of-fit on F^2	0.898	0.878
Final R indexes [I>=2σ (I)]	R_1 = 0.0853, wR_2 = 0.1136	R_1 = 0.0311, wR_2 = 0.0580
Final R indexes [all data]	R_1 = 0.2197, wR_2 = 0.1483	R_1 = 0.0495, wR_2 = 0.0620
Largest diff. peak/hole / e Å^{-3}	0.68/-0.71	0.25/-0.48
Flack	-	-0.03(3)
Diffractometer	STOE IPDS	STOE IPDS
CCDC access code	2329528	2329532

Table S1: Crystallographic data and data collection parameters (continued)

	$[Tc(NO)(Cp)(PPh_3)(py)](PF_6)$ (**6**(PF_6))	$[Tc(NO)(Cp)(PPh_3)(thioxane)](PF_6)$ (**7**(PF_6))
Empirical formula	$C_{28}H_{25}N_2OF_6P_2Tc$	$C_{27}H_{28}NO_2P_2STcF_6$
Formula weight	679.44	704.50
Temperature/K	100(2)	233(2)
Crystal system	Orthorhombic	Triclinic
Space group	$P2_12_12_1$	P-1
a/Å	9.9142(6)	10.5858(8)
b/Å	17.748(1)	10.7474(8)
c/Å	31.944(2)	13.300(1)
α/°	90	96.063(3)
β/°	90	102.373(3)
γ/°	90	107.210(3)
Volume/Å^3	5620.7(6)	1387.9(2)
Z	8	2
ρ_{calc} / gcm^{-3}	1.606	1.686
μ / mm^{-1}	0.691	0.777
F(000)	2736.0	712.0
Crystal size / mm^3	0.42 × 0.18 × 0.12	0.21 × 0.16 × 0.09
Radiation	MoKα (λ = 0.71073)	MoKα (λ = 0.71073)
2Θ range for data collection/°	4.46 to 54.30	4.55 to 54.54
Index ranges	-12 ≤ h ≤ 12, -22 ≤ k ≤ , -40 ≤ l ≤ 40	-13 ≤ h ≤ 13, -13 ≤ k ≤ 13, -17 ≤ l ≤ 17
Reflections collected	100174	38126
Independent reflections	12447 [R_{int} = 0.0240, R_{sigma} = 0.0153]	6183 [R_{int} = 0.0347, R_{sigma} = 0.0240]
Data/restraints/parameters	12447/0/721	6183/0/365
Goodness-of-fit on F^2	1.097	0.964
Final R indexes [I>=2σ (I)]	R_1 = 0.0238, wR_2 = 0.0585	R_1 = 0.0304, wR_2 = 0.0670
Final R indexes [all data]	R_1 = 0.0246, wR_2 = 0.0589	R_1 = 0.0381, wR_2 = 0.07007
Largest diff. peak/hole / e Å^{-3}	1.17/-0.90	1.23/-0.67
Flack	-0.015(3)	-
Diffractometer	Bruker APEX	Bruker APEX
CCDC access code	2329529	2329530

Table S1: Crystallographic data and data collection parameters (continued)

	[Tc(NO)(CpMe)(PPh3)(py)](PF6) (**10**(PF6))	[Tc(NO)(Cp)(PPh3)(NCCH3)](PF6) (**5**(PF6))<br>**For inspection only**
Empirical formula	$C_{29}H_{27}F_6N_2OP_2Tc$	$C_{25}H_{23}F_6N_2OP_6Tc$
Formula weight	693.46	641.39
Temperature/K	100(2)	100(2)
Crystal system	Monoclinic	Orthorhombic
Space group	Cc	Pbca
a/Å	17.944(2)	16.913(1)
b/Å	10.966(2)	20172(2)
c/Å	16.006(3)	15.329(1)
α/°	90	90
β/°	114.190(5)	90
γ/°	90	90
Volume/Å^3	2873.1(8)	5229.7(7)
Z	4	8
ρ_{calc}g / cm^3	1.603	1.629
μ / mm^{-1}	0.678	0.738
F(000)	1400.0	2576.0
Crystal size / mm^3	0.28 × 0.23 × 0.12	0.52 × 0.48 × 0.1
Radiation	MoKα (λ = 0.71073)	MoKα (λ = 0.71073)
2Θ range for data collection/°	4.47 to 54.32	4.816 to 49.998
Index ranges	-22 ≤ h ≤ 22, -14 ≤ k ≤ 14, -20 ≤ l ≤ 20	-20 ≤ h ≤ 18, -23 ≤ k ≤ 23, -18 ≤ l ≤ 18
Reflections collected	50841	03538
Independent reflections	6090 [R_{int} = 0.0259, R_{sigma} = 0.0160]	4589 [R_{int} = 0.0454, R_{sigma} = 0.0149]
Data/restraints/parameters	6090/2/370	4589/126/249
Goodness-of-fit on F^2	1.099	1.181
Final R indexes [I>=2σ (I)]	R_1 = 0.0154, wR_2 = 0.0389	R_1 = 0.1625, wR_2 = 0.3231
Final R indexes [all data]	R_1 = 0.0155, wR_2 = 0.0390	R_1 = 0.1658, wR_2 = 0.3247
Largest diff. peak/hole / e Å^{-3}	0.27/-0.46	2.11/-4.49
Flack	-0.014(4)	
Diffractometer	Bruker APEX	Bruker APEX
CCDC access code	2329531	2329533

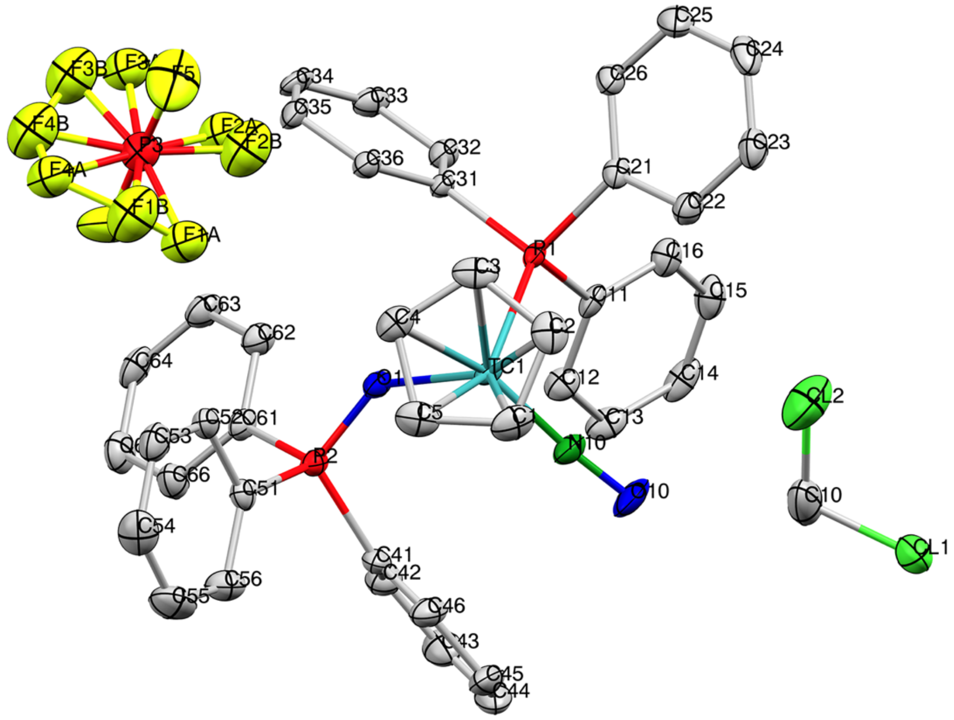

Figure S1. Ellipsoid representation of [Tc(NO)(Cp)(PPh_3)($OPPh_3$)](PF_6) x CH_2Cl_2 including the positional disorder in the PF_6^- counter ion. The thermal ellipsoids are set at a 50% probability level. Hydrogen atoms are omitted for clarity.

Table S2. Selected bond lengths (Å) and angles (°) in the [Tc(NO)(Cp)(PPh_3)($OPPh_3$)]$^+$ cation.

Tc1–O1	2.149(3)	Tc1–P1	2.299(1)	Tc1–N10	1.755(4)
N10–O10	1.184(6)	O1–P2		Tc1–C1	2.235(5)
Tc1–C2	2.239(5)	Tc1–C3	2.307(8)	Tc1–C4	2.318(5)
Tc1–C5	2.275(6)				
Tc1–N10–O10	170.4(4)	Tc1–O1–P2	133.3(2)	P1–Tc1–N10	91.4(2)
P1–Tc1–O1	81.7(1)	N10–Tc1–O1	102.4(2)		

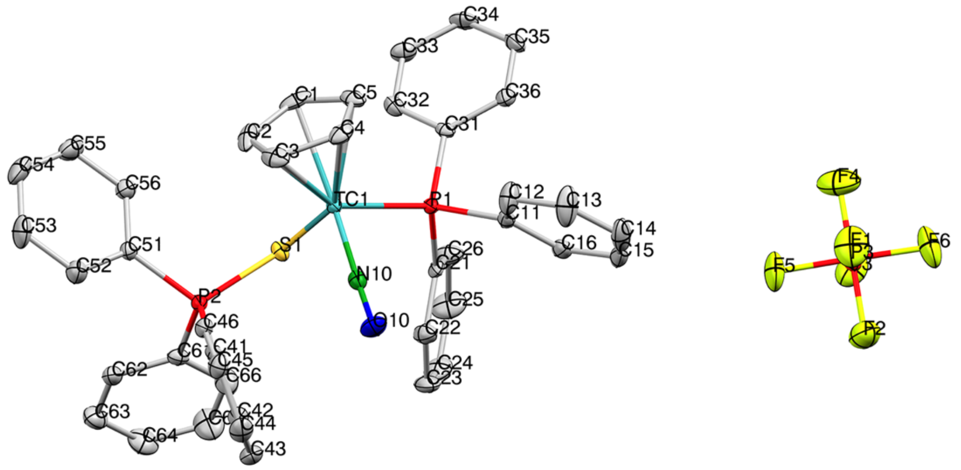

Figure S2. Ellipsoid representation of [Tc(NO)(Cp)(PPh$_3$)(SPPh$_3$)](PF$_6$). The thermal ellipsoids are set at a 50% probability level. Hydrogen atoms are omitted for clarity.

Table S3. Selected bond lengths (Å) and angles (°) in the [Tc(NO)(Cp)(PPh$_3$)(SPPh$_3$)]$^+$ cation.

Tc1–S1	2.4242(6)	Tc1–P1	2.3726(6)	Tc1–N10	1.768(2)
N10–O10	1.177(3)	S1–P2	2.0237(8)	Tc1–C1	2.282(3)
Tc1–C2	2.287(3)	Tc1–C3	2.300(2)	Tc1–C4	2.278(2)
Tc1–C5	2.279(2)				
Tc1–N10–O10	174.0(2)	Tc1–S1–P2	109.22(3)	P1–Tc1–N10	91.45(6)
P1–Tc1–S1	85.42(2)	N10–Tc1–S1	98.65(6)		

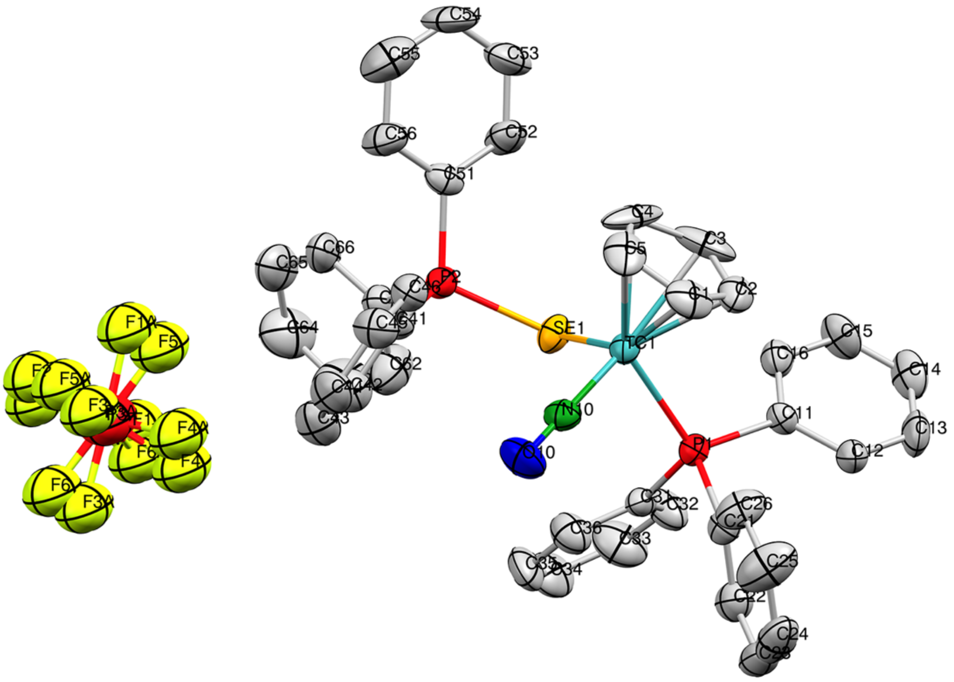

Figure S3. Ellipsoid representation of $[Tc(NO)(Cp)(PPh_3)(SePPh_3)](PF_6)$ including the positional disorder in the PF_6^- counter ion. The thermal ellipsoids are set at a 50% probability level. Hydrogen atoms are omitted for clarity.

Table S4. Selected bond lengths (Å) and angles (°) in the $[Tc(NO)(Cp)(PPh_3)(SePPh_3)]^+$ cation.

Tc1–Se1	2.530(1)	Tc1–P1	2.381(2)	Tc1–N10	1.757(7)
N10–O10	1.186(8)	Se1–P2	2.183(2)	Tc1–C1	2.266(8)
Tc1–C2	2.245(9)	Tc1–C3	2.289(9)	Tc1–C4	2.307(9)
Tc1–C5	2.275(8)				
Tc1–N10–O10	174.8(6)	Tc1–Se1–P2	107.36(7)	P1–Tc1–N10	91.5(2)
P1–Tc1–Se1	85.58(6)	N10–Tc1–Se1	98.9(2)		

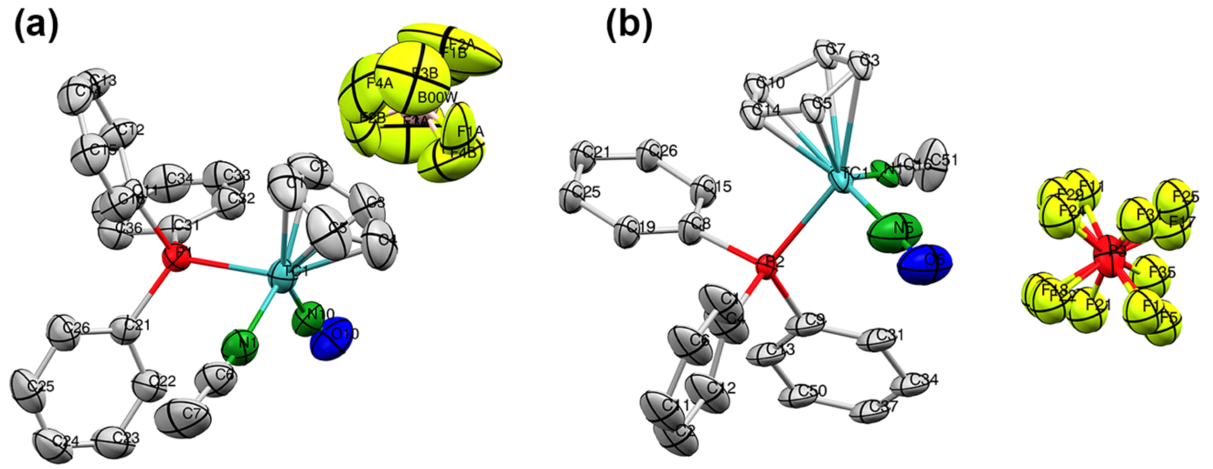

Figure S4. (a) Ellipsoid representation of [Tc(NO)(Cp)(PPh$_3$)(NCCH$_3$)](BF$_4$) including the positional disorder in the BF$_4^-$ counter ion. The thermal ellipsoids are set at a 50% probability level. (b) Ellipsoid representation of [Tc(NO)(Cp)(PPh$_3$)(NCCH$_3$)](PF$_6$) including the positional disorder in the PF$_6^-$ counter ion. The thermal ellipsoids are set at a 50% probability level. Hydrogen atoms are omitted for clarity. The refinement converged at an unsatisfactory R$_1$ value of 0.1625, for which reason the structural data should not been deposited with the CCDC data base.

Table S5. Selected bond lengths (Å) and angles (°) in the [Tc(NO)(Cp)(PPh$_3$)(NCCH$_3$)]$^+$ cations in the BF$_4^-$ and PF$_6^-$ salts.

BF$_4^-$ salt					
Tc1–N1	2.094(4)	Tc1–P1	2.385(1)	Tc1–N10	1750(4)
N10–O10	1.196(5)	Tc1–C1	2.263(6)	Tc1–C2	2.218(6)
Tc1–C3	2.228(6)	Tc1–C4	2.295(6)	Tc1–C5	2.313(6)
Tc1–N10–O10	168.4(4)	Tc1–N1–C6	170.4(5)	P1–Tc1–N10	92.6(1)
P1–Tc1–N41	154.5(2)				
PF$_6^-$ salt					
Tc1–N41	2.09(1)	Tc1–P1	2.384(4)	Tc1–N10	1.68(2)
N10–O10	1.10(3)	Tc1–C1	2.31(2)	Tc1–C2	2.26(2)
Tc1–C3	2.32(2)	Tc1–C4	2.39(2)	Tc1–C5	2.39(2)
Tc1–N10–O10	170(3)	Tc1–N41–C42	175(2)	P1–Tc1–N10	97.2(8)
P1–Tc1–N41	87.0(4)				

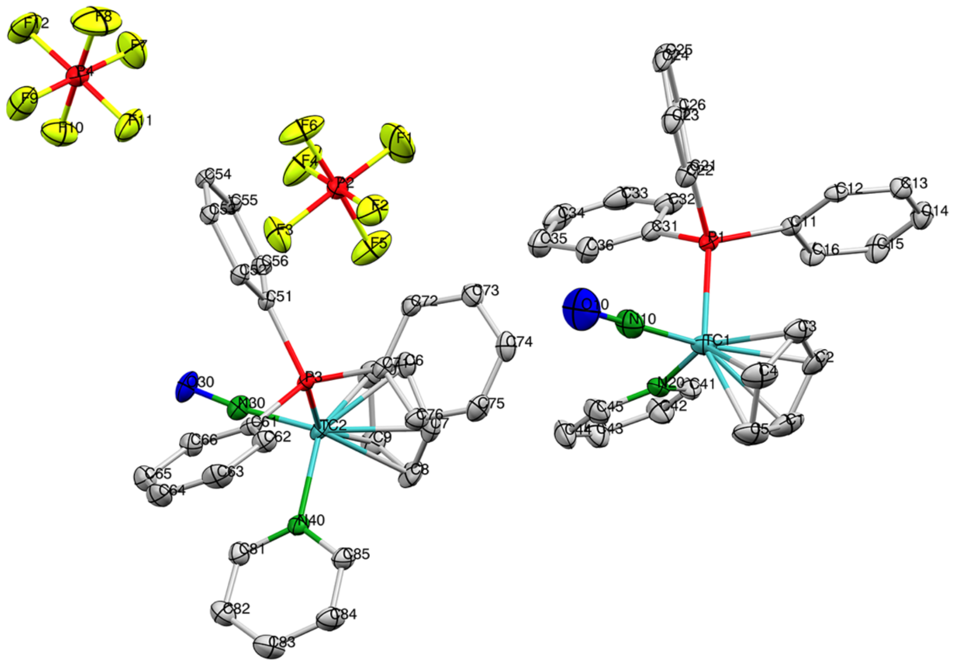

Figure S5. Ellipsoid representation of [Tc(NO)(Cp)(PPh_3)(py)](PF_6). including the positional disorder in the PF_6^- counter ion. The thermal ellipsoids are set at a 50% probability level. Hydrogen atoms are omitted for clarity.

Table S6. Selected bond lengths (Å) and angles (°) in the [Tc(NO)(Cp)(PPh_3)(py)]$^+$ cation.

Tc1–N20		Tc1–P1	2.3855(8)	Tc1–N10	1.752(3)
N10–O10	1.175(5)	Tc1–C1	2.336(4)	Tc1–C2	2.304(4)
Tc1–C3	2.236(4)	Tc1–C4	2.244(4)	Tc1–C5	2.298(4)
Tc2–N40	2.150(3)	Tc2–P3	2.3797(8)	Tc2–N30	1.763(3)
N30–O30	1.186(4)	Tc2–C6	2.240(3)	Tc2–C7	2.266(3)
Tc2–C8	2.312(3)	Tc2–C9	2.309(3)	Tc2–C10	2.259(3)
Tc1–N10–O10	176.9(4)	P1–Tc1–N10	91.4(1)	P1–Tc1–N20	89.78(8)
N10–Tc1–N20	94.5(1)	Tc2–N30–O30	173.5(3)	P3–Tc2–N30	89.95(9)
P3–Tc2–N40	90.08(7)	N30–Tc2–N40	97.9(1)		

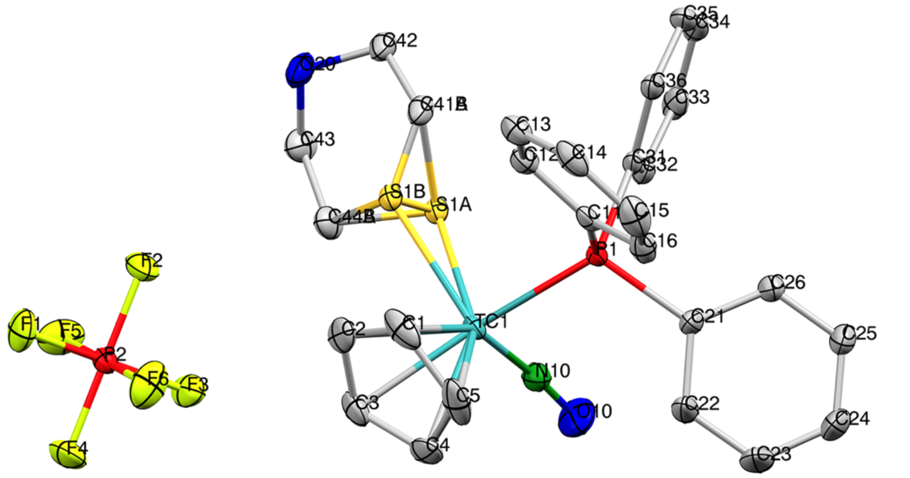

Figure S6. Ellipsoid representation of [Tc(NO)(Cp)(PPh_3)(thioxane)](PF_6). including the positional disorder of the S(CH_2)$_2$ unit of the thioxane ligand. The thermal ellipsoids are set at a 50% probability level. Hydrogen atoms are omitted for clarity.

Table S7. Selected bond lengths (Å) and angles (°) in the [Tc(NO)(Cp)(PPh_3)(thioxane)]$^+$ cation.

Tc1–S1A	2.4070(6)	Tc1–P1	2.3805(6)	Tc1–N10	1.771(2)
N10–O10	1.174(3)	Tc1–C1	2.307(3)	Tc1–C2	2.326(3)
Tc1–C3	2.285(2)	Tc1–C4	2.244(3)	Tc1–C5	2.251(3)
Tc1–N10–O10	172.7(2)	P1–Tc1–N10	90.03(7)	P1–Tc1–S1A	91.86(2)
N10–Tc1–S1A	93.20(7)				

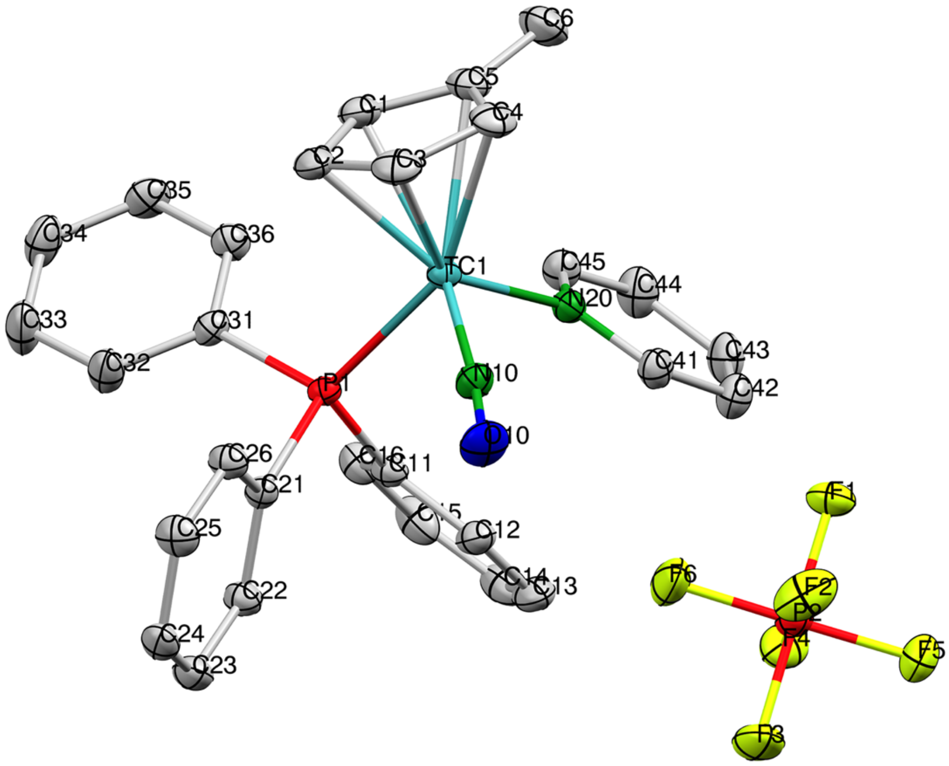

Figure S7. Ellipsoid representation of [Tc(NO)(MeCp)(PPh$_3$)(py)](PF$_6$). The thermal ellipsoids are set at a 50% probability level. Hydrogen atoms are omitted for clarity.

Table S8. Selected bond lengths (Å) and angles (°) in the [Tc(NO)(MeCp)(PPh$_3$)(py)]$^+$ cation.

Tc1–N20	2.161(2)	Tc1–P1	2.3925(7)	Tc1–N10	1.762(2)
N10–O10	1.175(3)	Tc1–C1	2.291(2)	Tc1–C2	2.224(2)
Tc1–C3	2.225(2)	Tc1–C4	2.300(2)	Tc1–C5	2.362(2)
Tc1–N10–O10	169.3(2)	P1–Tc1–N10	90.22(6)	P1–Tc1–N20	89.05(5)
N10–Tc1–N20	98.10(8)				

Spectroscopic data

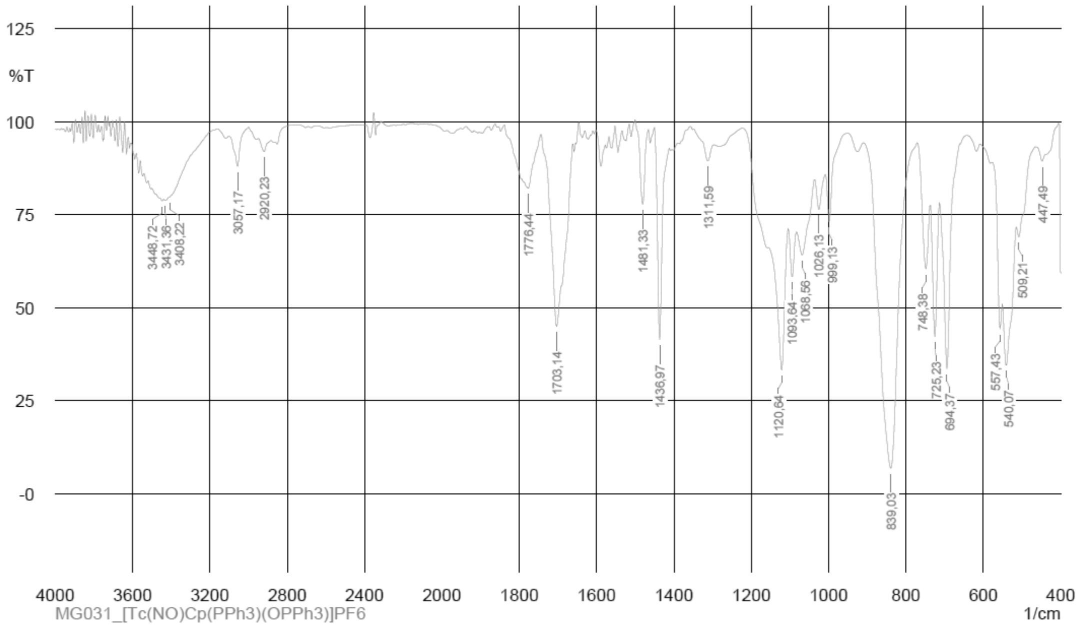

Figure S8: IR (KBr) spectrum of $[Tc(NO)(Cp)(PPh_3)(OPPh_3)](PF_6)$.

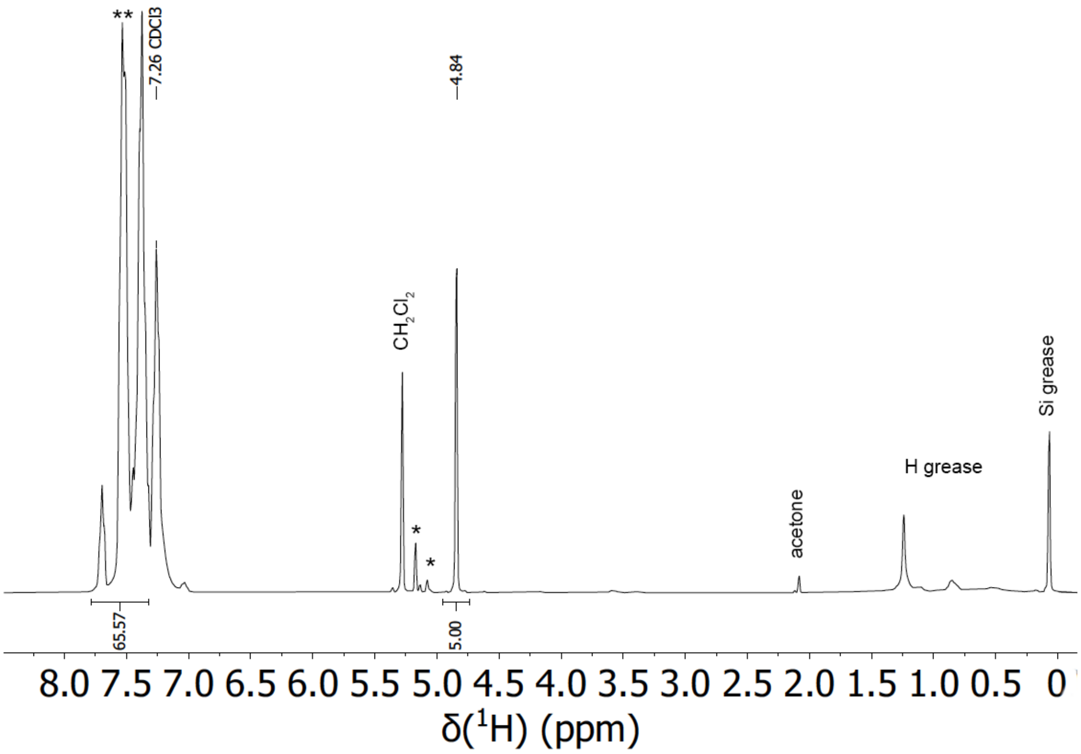

Figure S9: ^1H NMR spectrum of $[Tc(NO)(Cp)(PPh_3)(OPPh_3)](PF_6)$ in $CDCl_3$ (* traces of potentially formed *o*-$OPPh_3CH_2Cl$ or similar decomposition products; ** $OPPh_3$ impurity) Identified impurities and solvents are annotated.

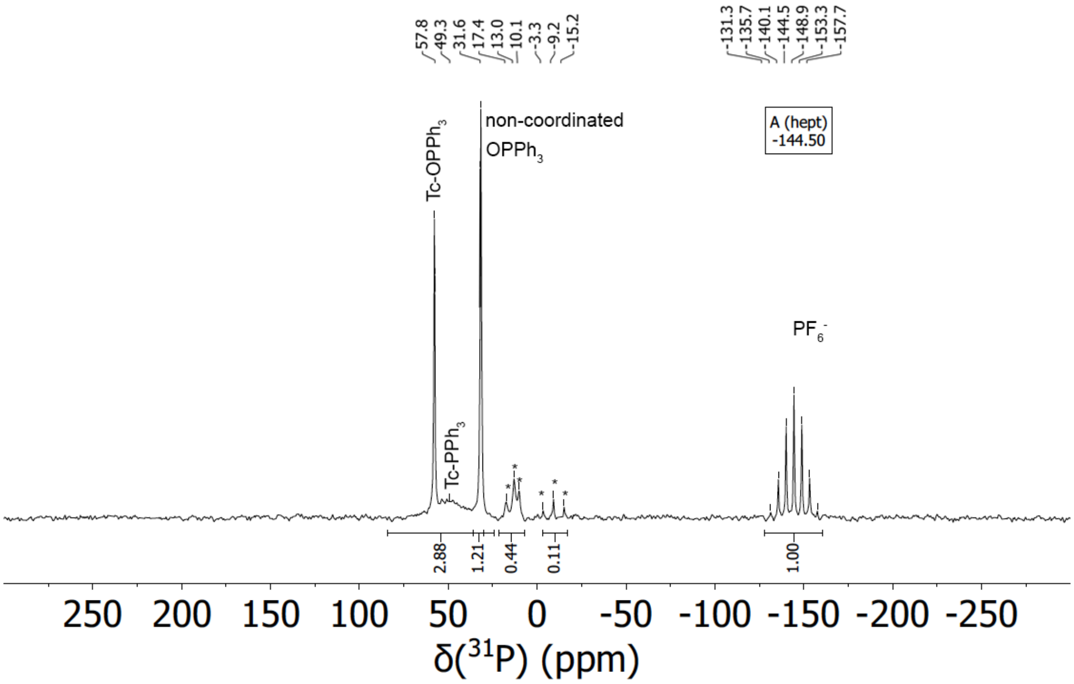

Figure S10: $^{31}P\{^{1}H\}$ NMR spectrum of $[Tc(NO)(Cp)(PPh_3)(OPPh_3)](PF_6)$ in $CDCl_3$ (* potentially formed *o*-$OPPh_3CH_2Cl$ or similar decomposition products). Identified impurities are annotated. An uncommonly large exponential apodization function for ^{31}P NMR was applied (100 Hz) after truncation of the FID at 30k points and zero-filling to the original 256k points to enable an interpretation of the very broad resonance for the coordinated PPh_3 ligand.

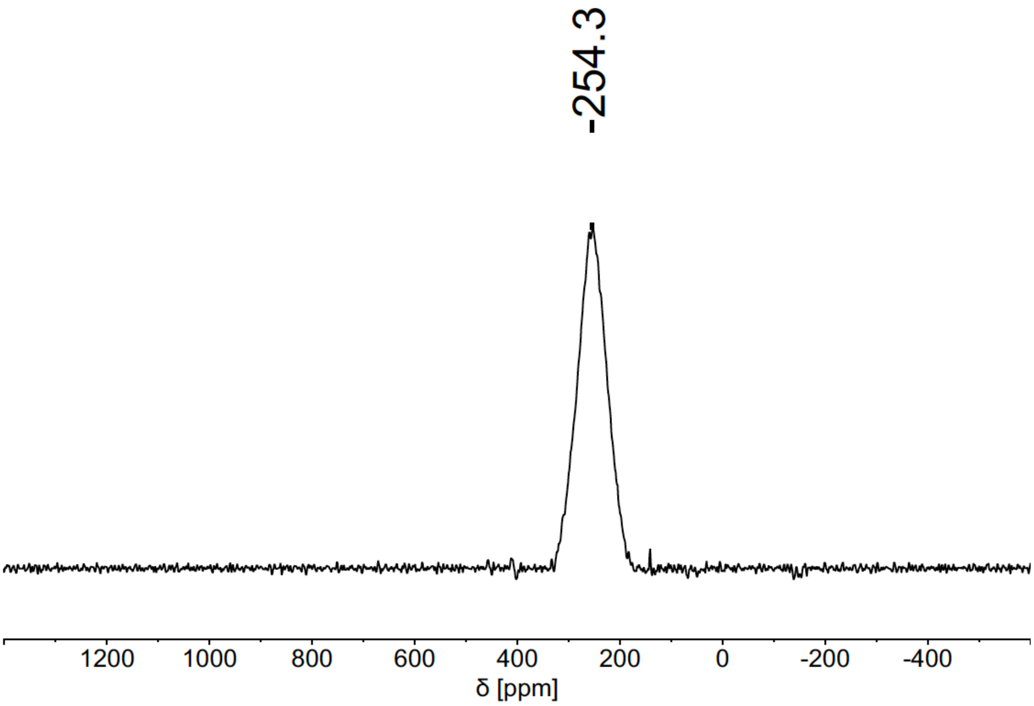

Figure S11: ^{99}Tc NMR spectrum of $[Tc(NO)(Cp)(PPh_3)(OPPh_3)](PF_6)$ in $CDCl_3$.

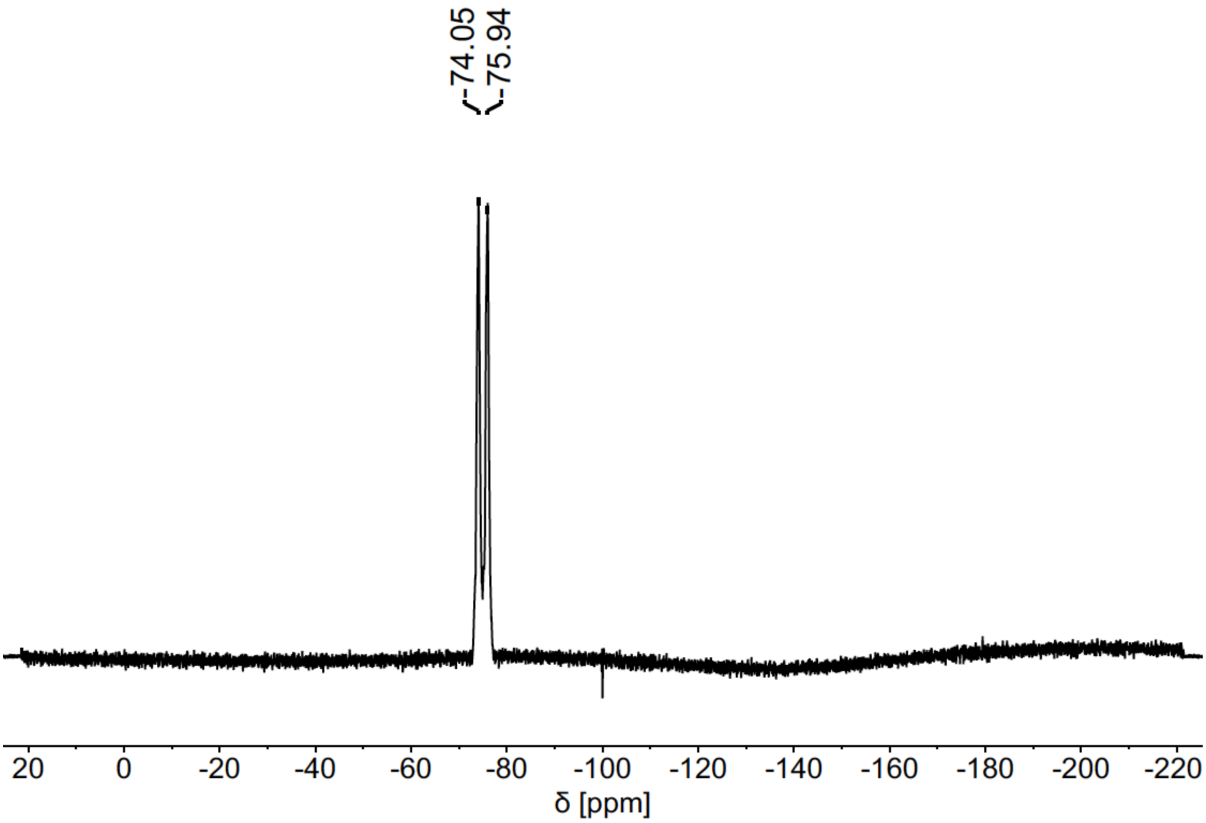

Figure S12: ^{19}F NMR spectrum of $[Tc(NO)(Cp)(PPh_3)(OPPh_3)](PF_6)$ in $CDCl_3$.

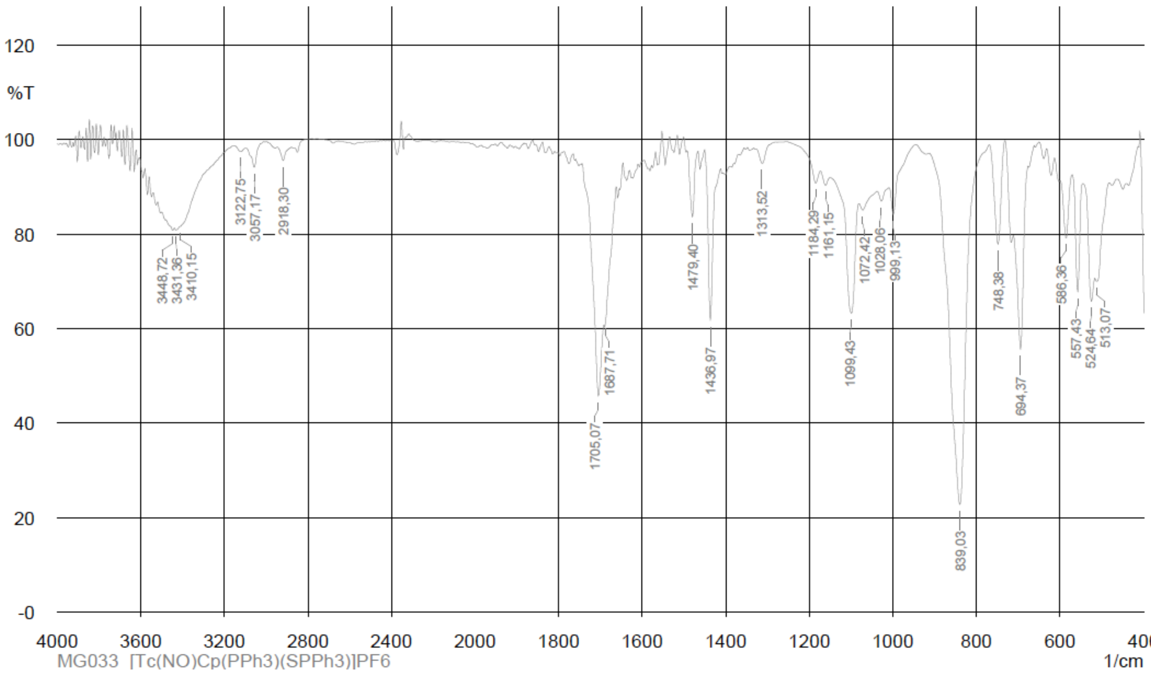

Figure S13: IR (KBr) spectrum of $[Tc(NO)(Cp)(PPh_3)(SPPh_3)](PF_6)$.

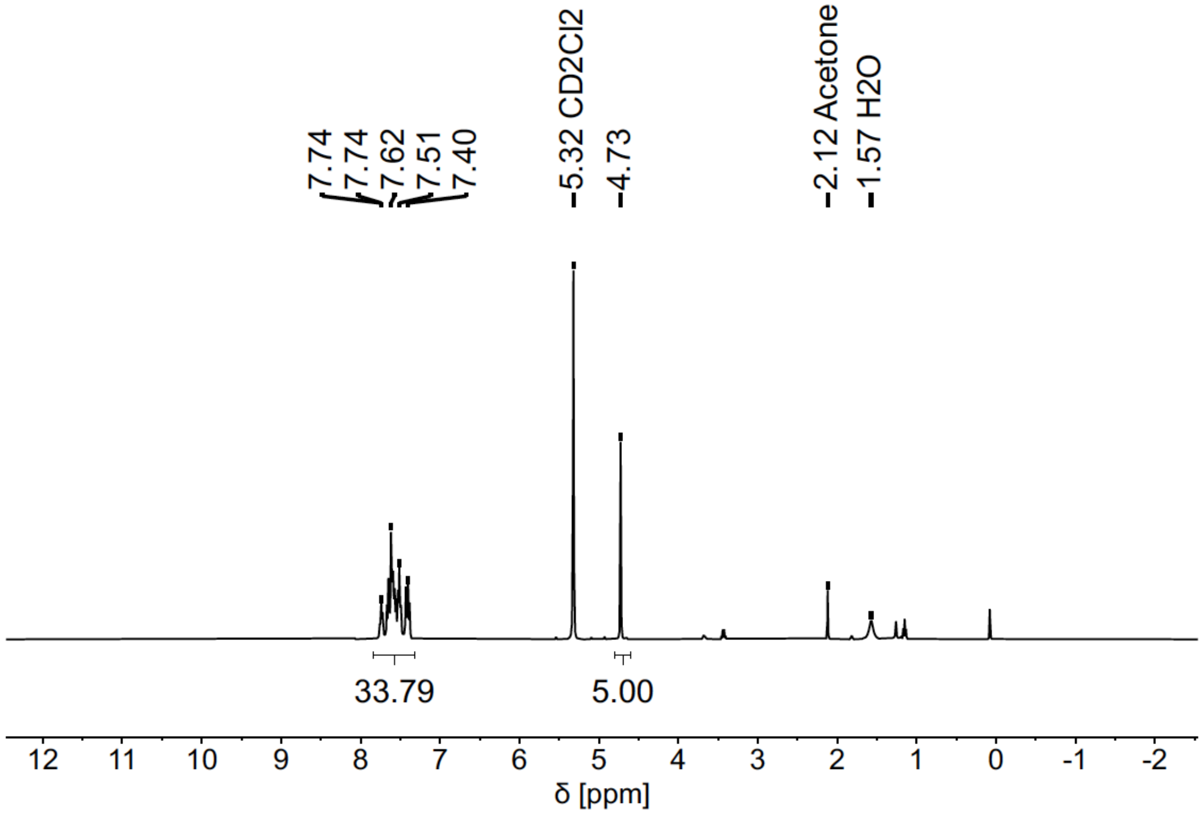

Figure S14: ^{1}H NMR spectrum of [Tc(NO)(Cp)(PPh_3)($SPPh_3$)](PF_6) in CD_2Cl_2. Identified solvents are annotated.

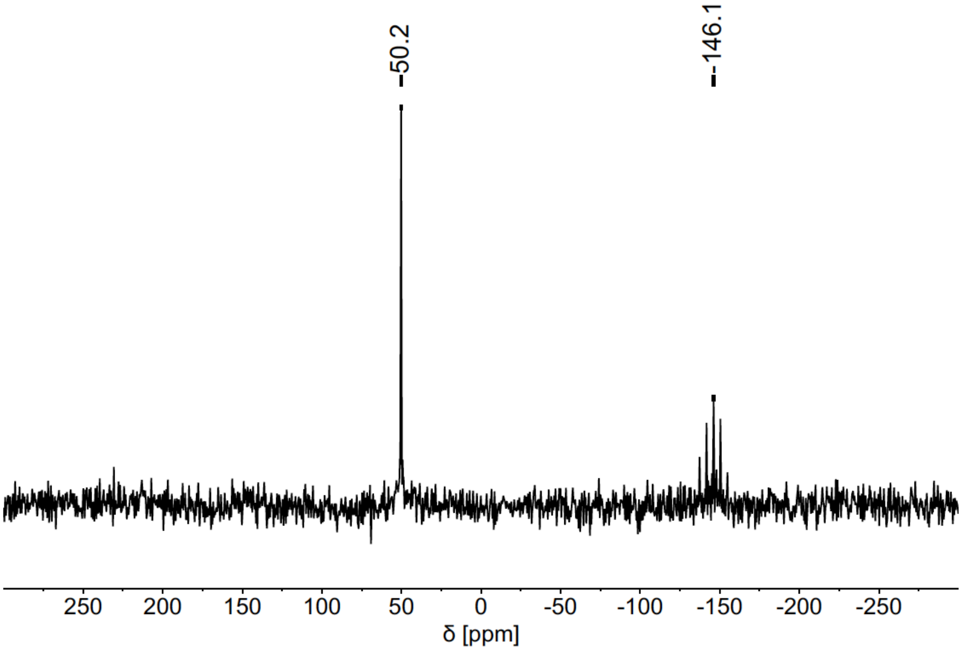

Figure S15: $^{31}P\{^{1}H\}$ NMR spectrum of [Tc(NO)(Cp)(PPh_3)($SPPh_3$)](PF_6) in CD_2Cl_2.

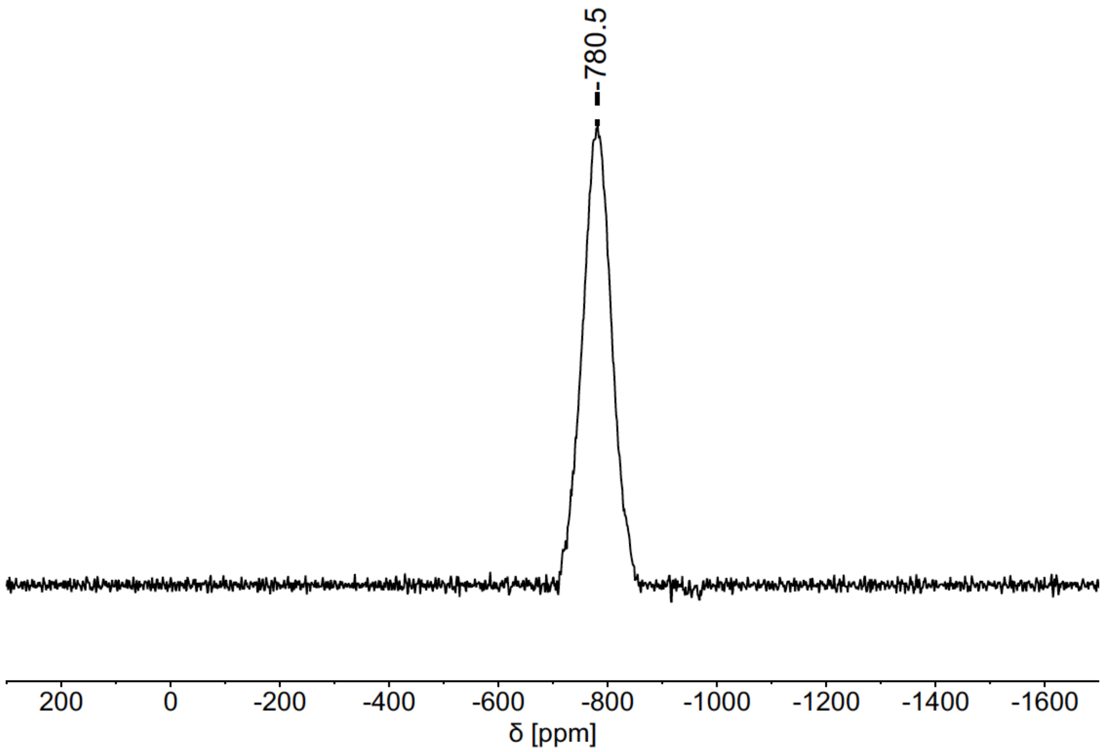

Figure S16: ^{99}Tc NMR spectrum of [Tc(NO)(Cp)(PPh_3)($SPPh_3$)](PF_6) in CD_2Cl_2.

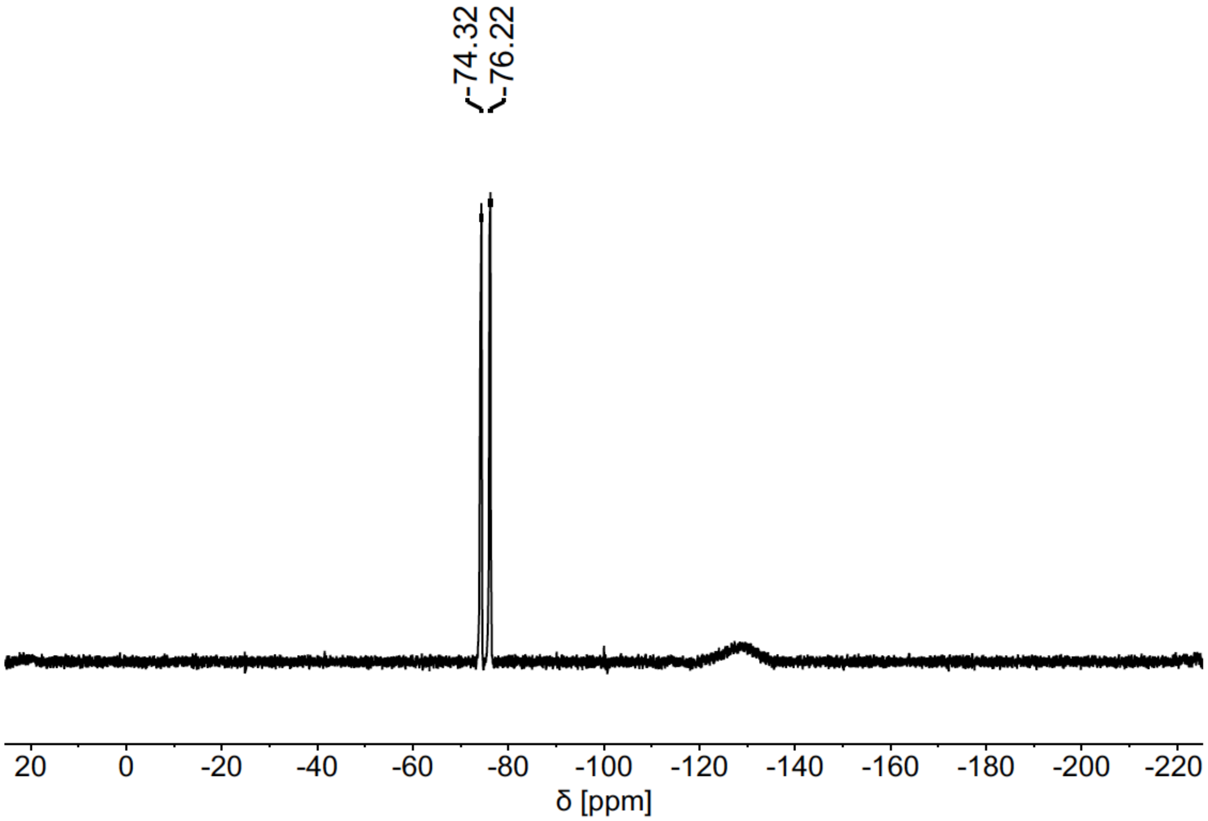

Figure S17: ^{19}F NMR spectrum of [Tc(NO)(Cp)(PPh_3)($SPPh_3$)](PF_6) in CD_2Cl_2.

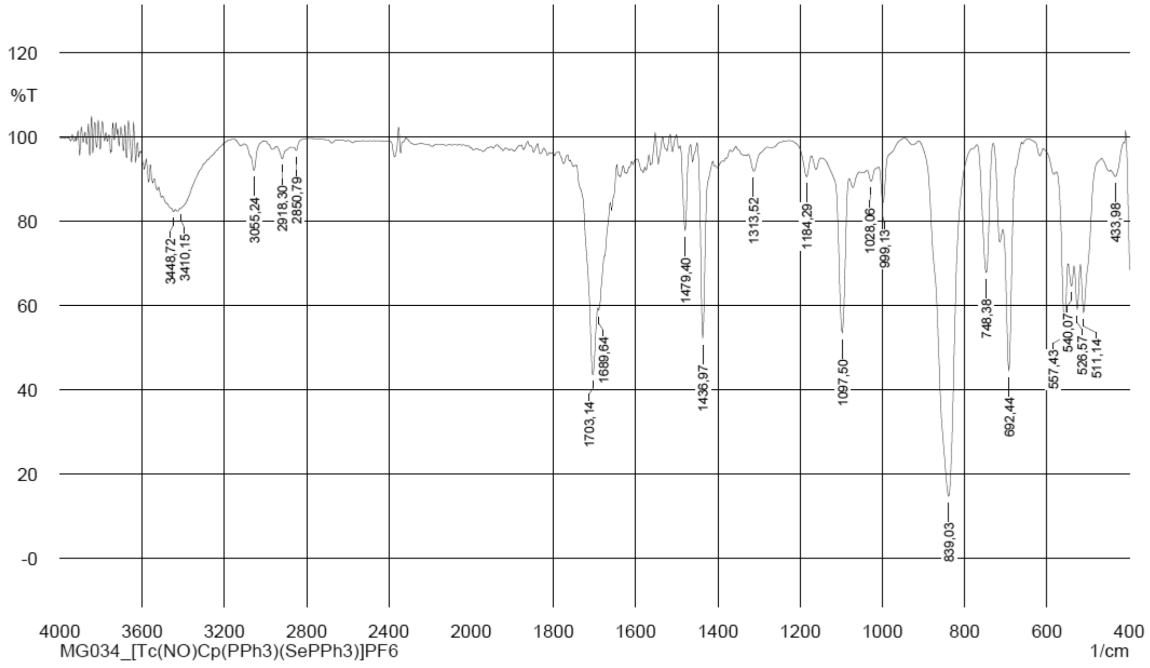

Figure S18: IR (KBr) spectrum of [Tc(NO)(Cp)(PPh_3)($SePPh_3$)](PF_6).

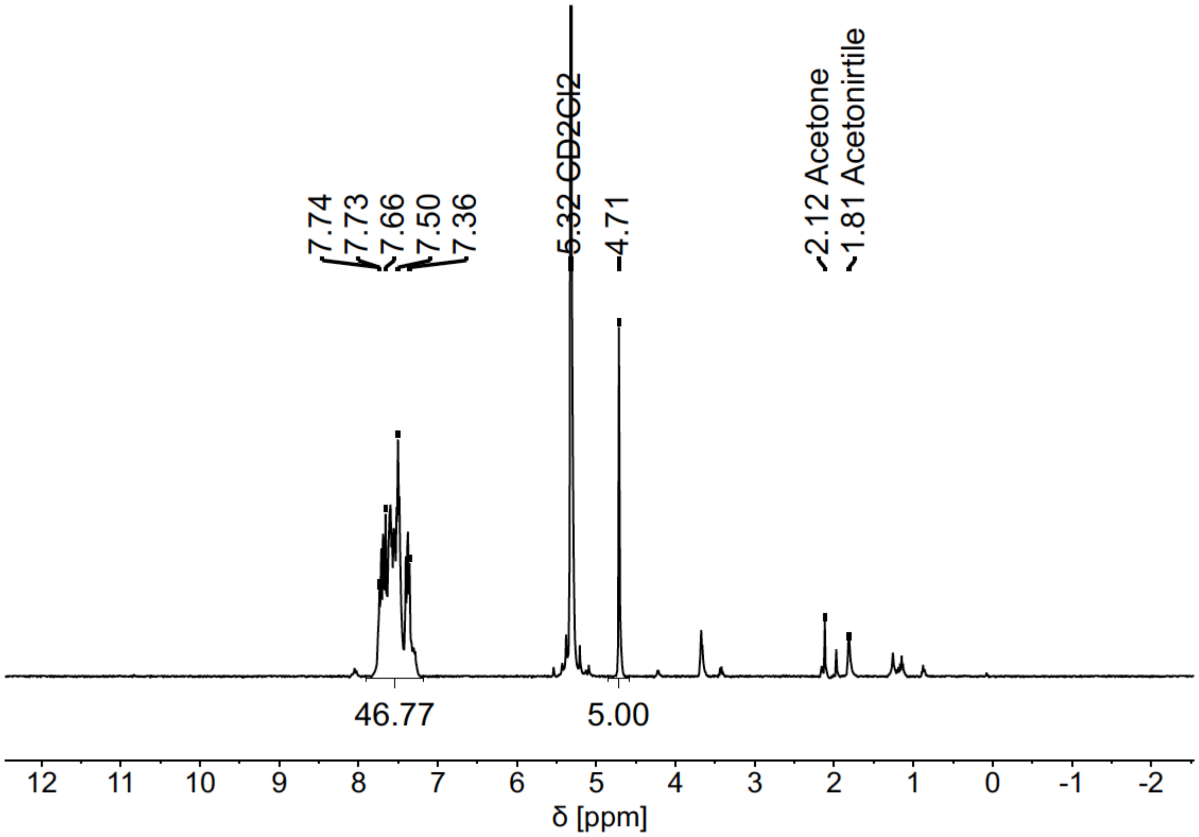

Figure S19: ^{1}H NMR spectrum of [Tc(NO)(Cp)(PPh_3)($SePPh_3$)](PF_6) in CD_2Cl_2. Identified solvent impurities are annotated.

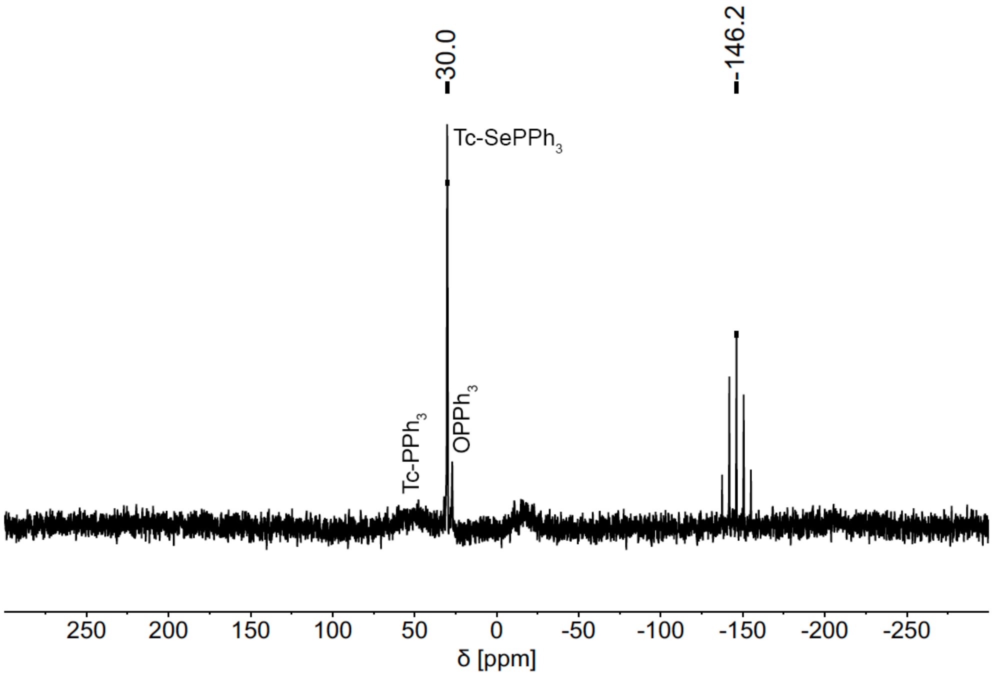

Figure S20: $^{31}P\{^{1}H\}$ NMR spectrum of [Tc(NO)(Cp)(PPh_3)($SePPh_3$)](PF_6) in CD_2Cl_2. Identified impurities are annotated.

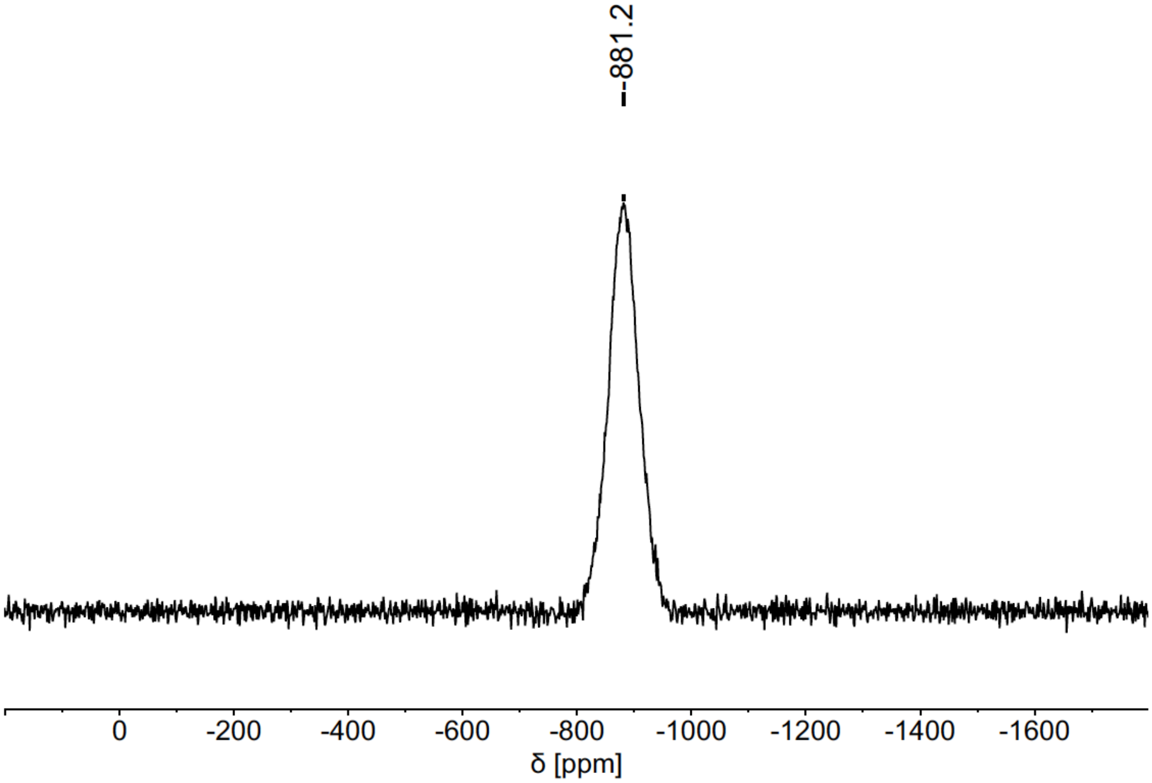

Figure S21: ^{99}Tc NMR spectrum of [Tc(NO)(Cp)(PPh_3)($SePPh_3$)](PF_6) in CD_2Cl_2.

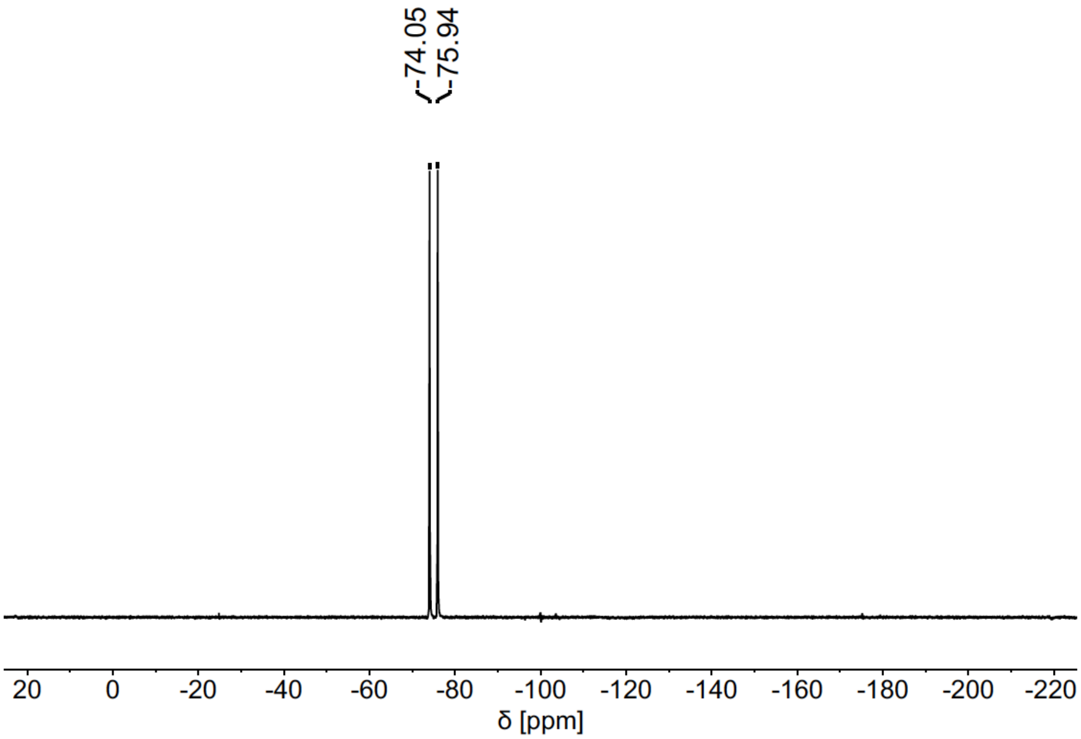

Figure S22: ^{19}F NMR spectrum of $[Tc(NO)(Cp)(PPh_3)(SePPh_3)](PF_6)$ in CD_2Cl_2.

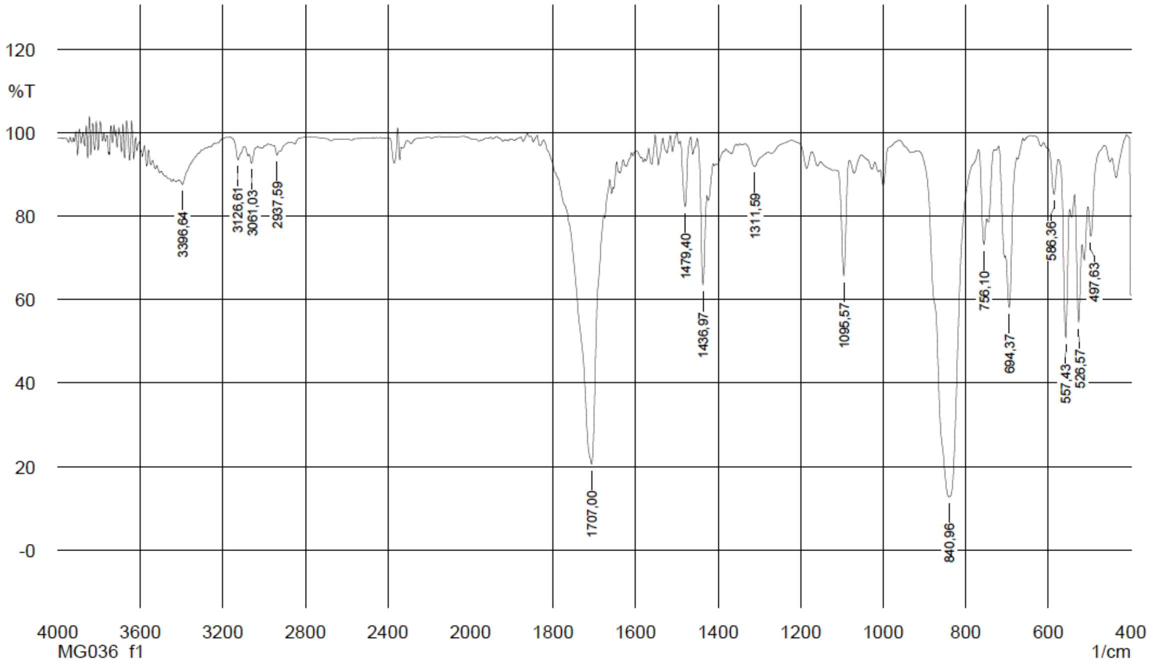

Figure S23: IR (KBr) spectrum of $[Tc(NO)(Cp)(PPh_3)(NCCH_3)](PF_6)$.

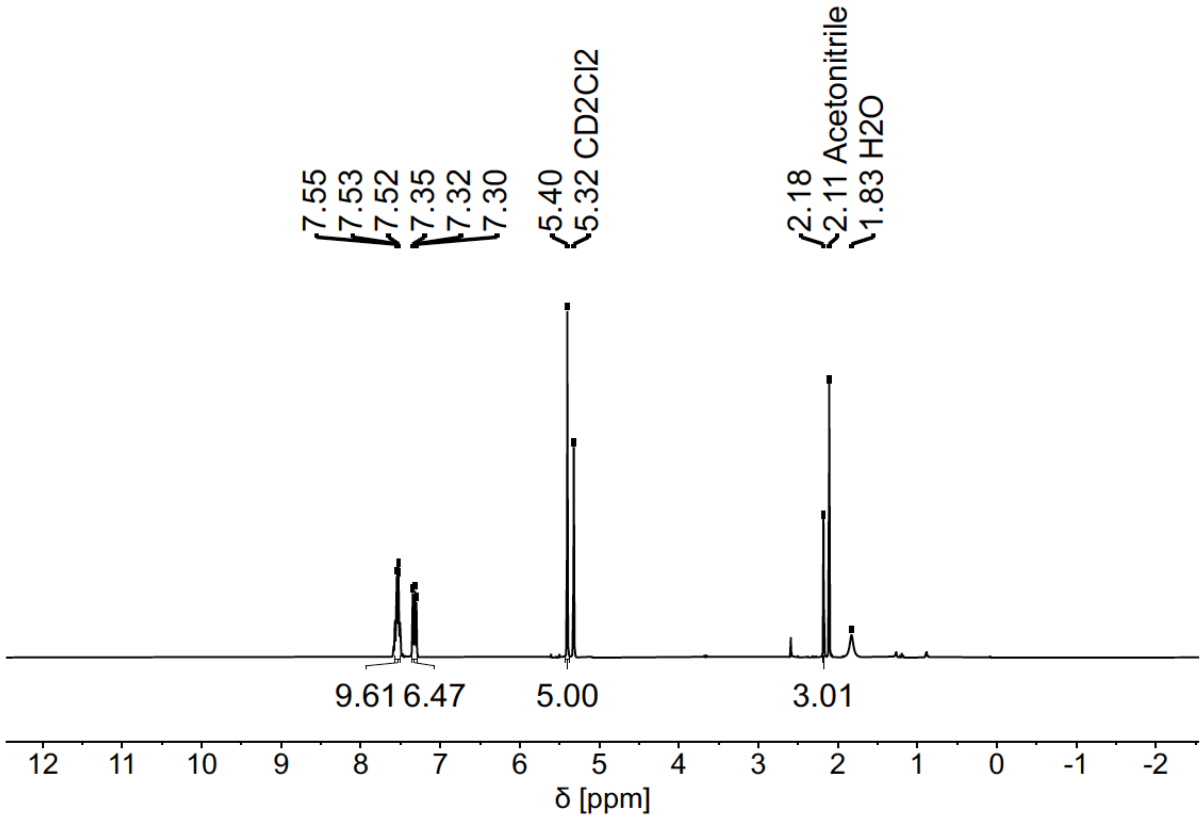

Figure S24: ^{1}H NMR spectrum of $[Tc(NO)(Cp)(PPh_3)(NCCH_3)](BF_4)$ in CD_2Cl_2. Identified impurities and solvents are annotated.

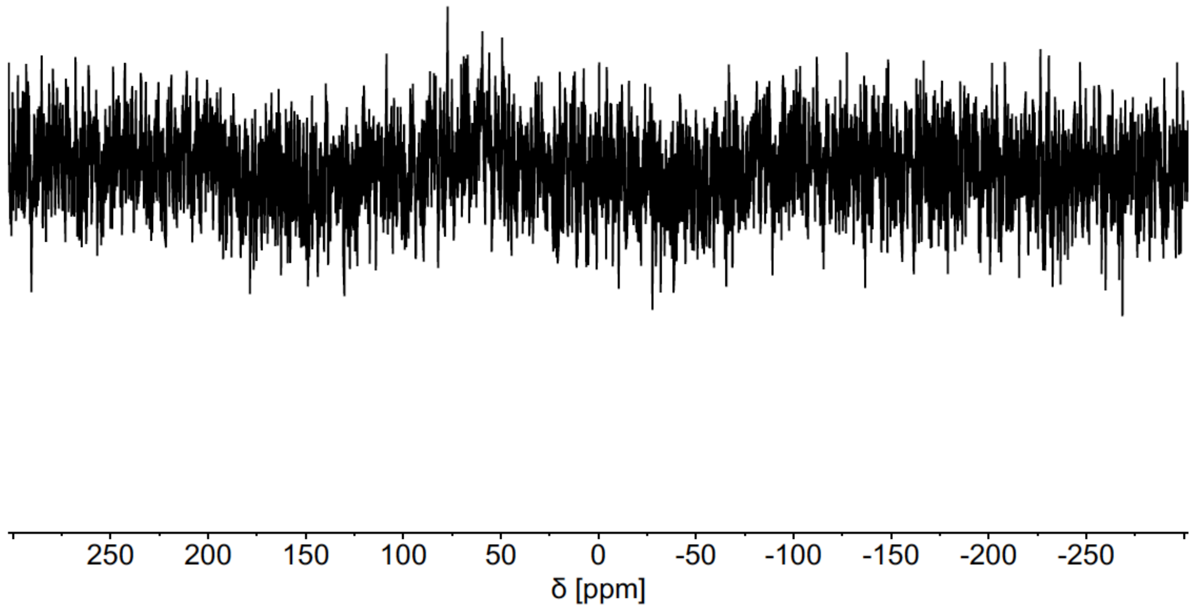

Figure S25: $^{31}P\{^{1}H\}$ NMR spectrum of $[Tc(NO)(Cp)(PPh_3)(NCCH_3)](BF_4)$ in CD_2Cl_2.

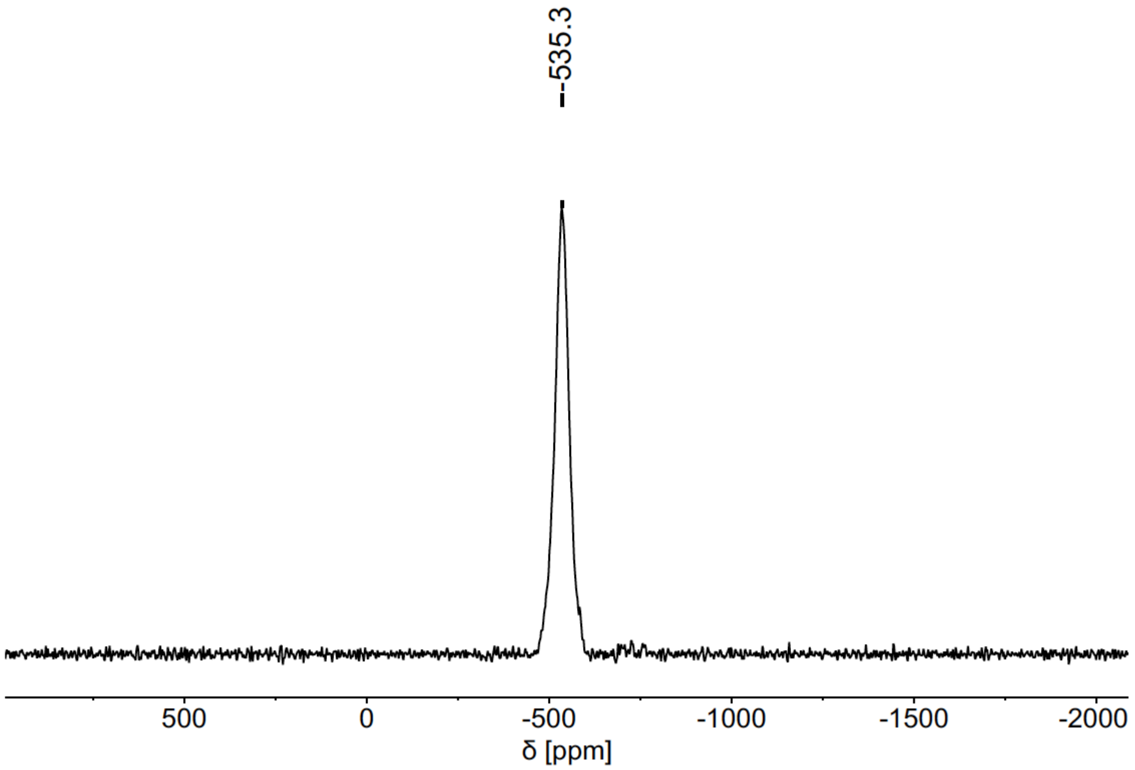

Figure S26: ^{99}Tc NMR spectrum of $[Tc(NO)(Cp)(PPh_3)(NCCH_3)](BF_4)$ in CD_2Cl_2.

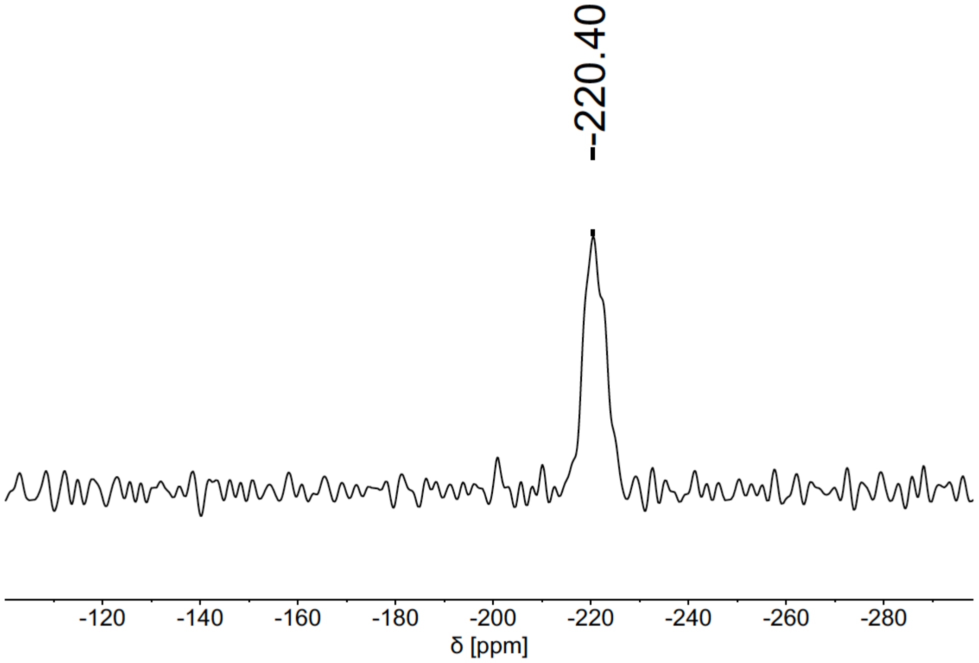

Figure S27: ^{15}N NMR spectrum of $[Tc(NO)(Cp)(PPh_3)(^{15}N\text{-}NCCH_3)](BF_4)$ in acetone-d_6.

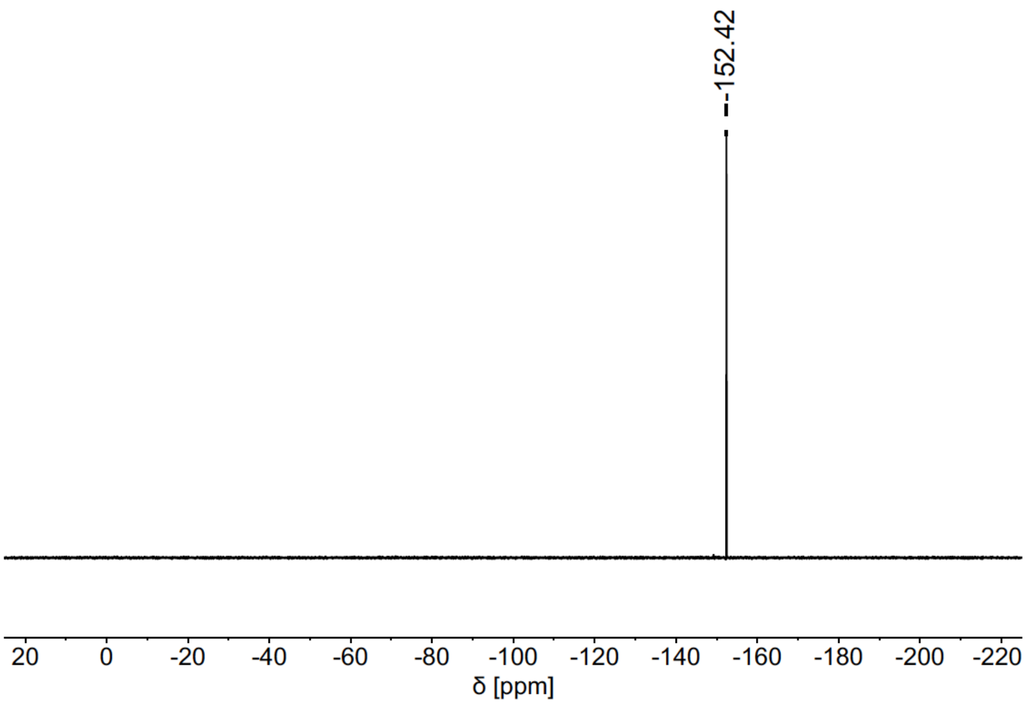

Figure S28: ^{19}F NMR spectrum of $[Tc(NO)(Cp)(PPh_3)(NCCH_3)](BF_4)$ in CD_2Cl_2.

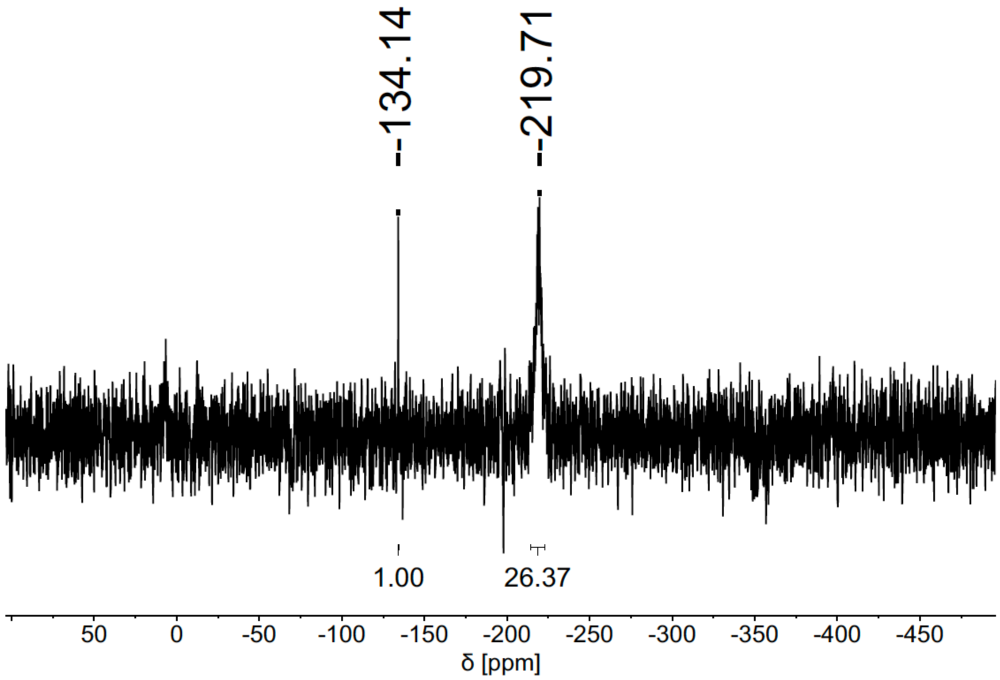

Figure S29: ^{15}N NMR spectrum of a 1:1 reaction mixture of $[Tc(NO)(Cp)(PPh_3)(^{15}N\text{-}NCCH_3)](BF_4)$ and acetonitrile with natural isotopic abundance at room temperature in acetone-d_6.

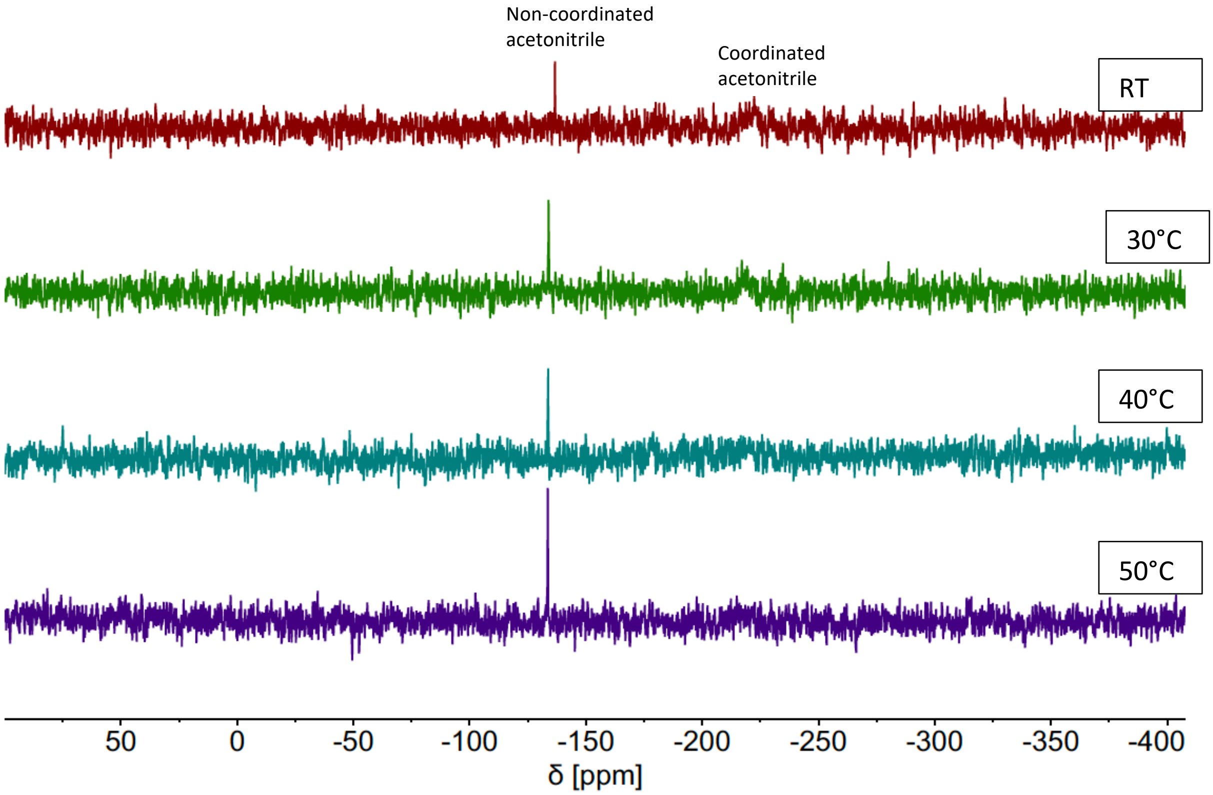

Figure S30: ^{15}N NMR spectra of a 1:1 reaction mixture of [Tc(NO)(Cp)(PPh_3)(^{15}N-$NCCH_3$)](BF_4) and acetonitrile with natural isotopic abundance in acetone-d_6 at various temperatures.

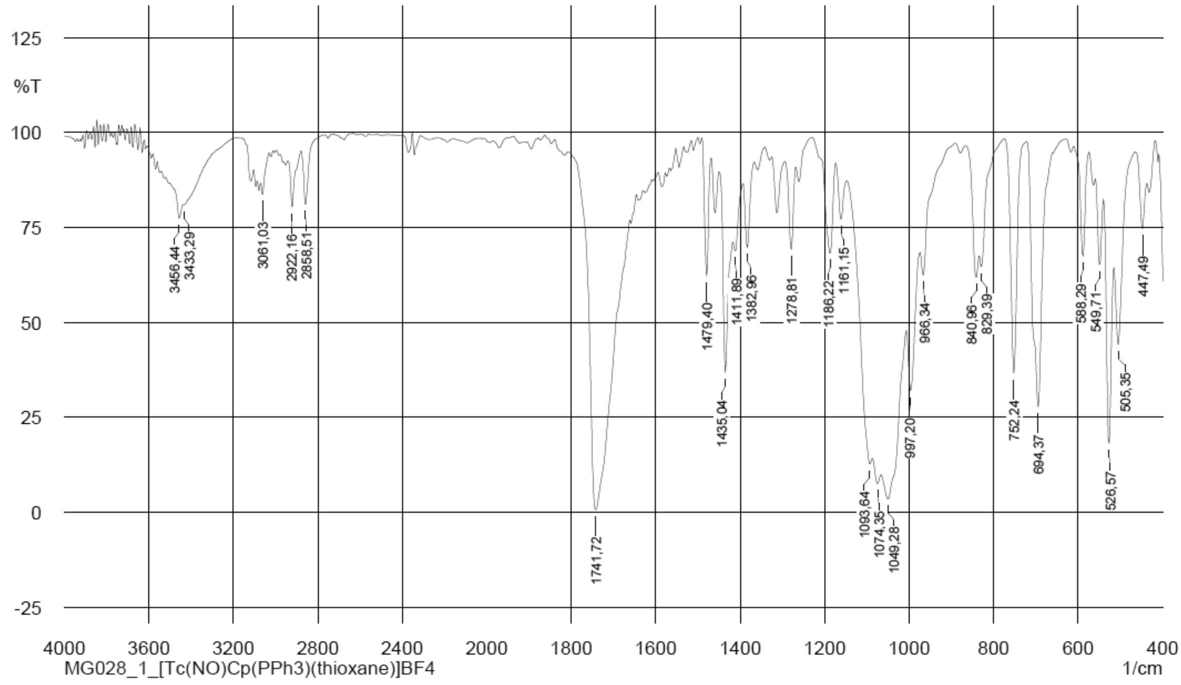

Figure S31: IR (KBr) spectrum of [Tc(NO)(Cp)(PPh_3)(thioxane)](BF_4).

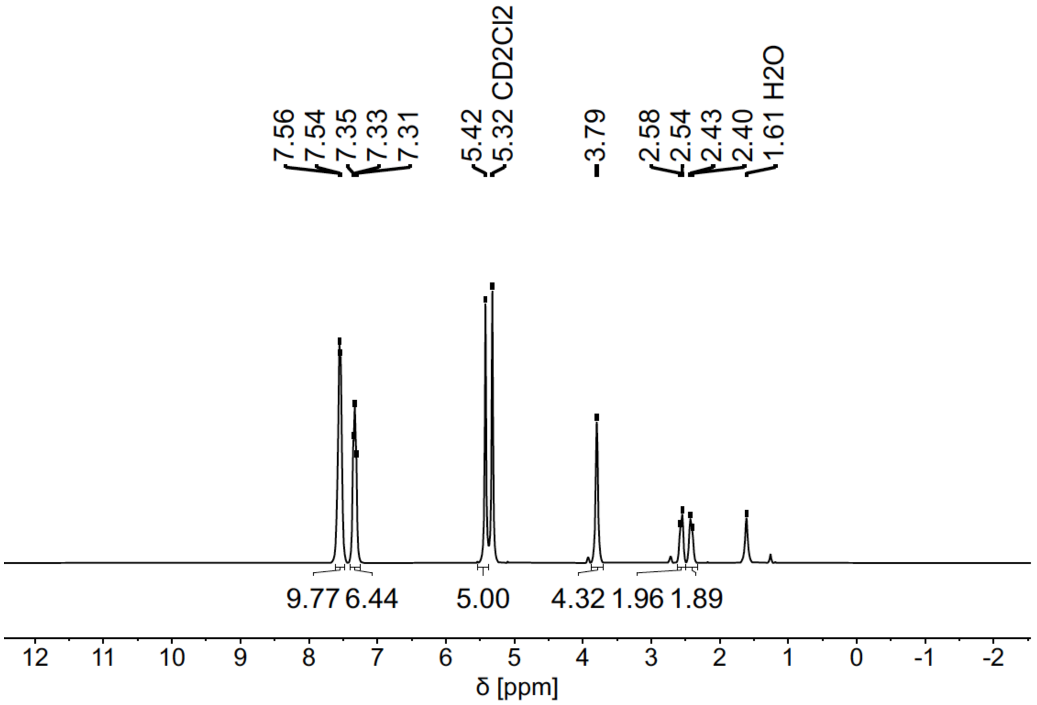

Figure S32: ^{1}H NMR spectrum of $[Tc(NO)(Cp)(PPh_3)(thioxane)](BF_4)$ in CD_2Cl_2. Identified impurities and solvents are annotated.

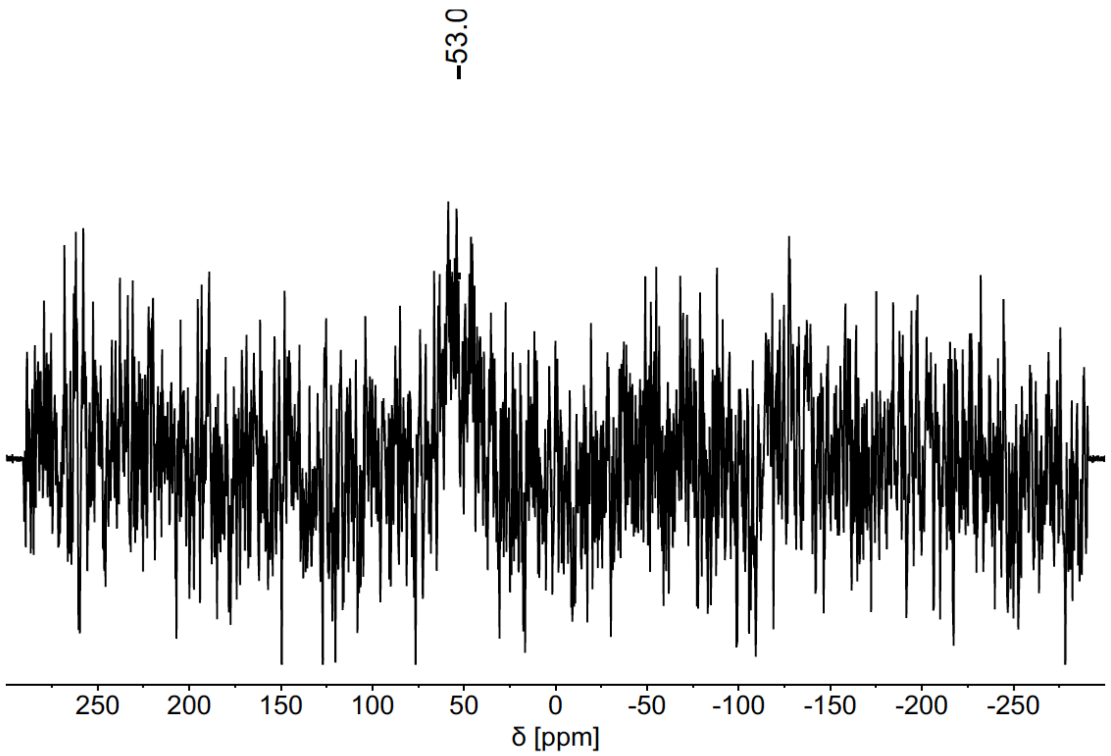

Figure S33: $^{31}P\{^{1}H\}$ NMR spectrum of $[Tc(NO)(Cp)(PPh_3)(thioxane)](BF_4)$ in CD_2Cl_2.

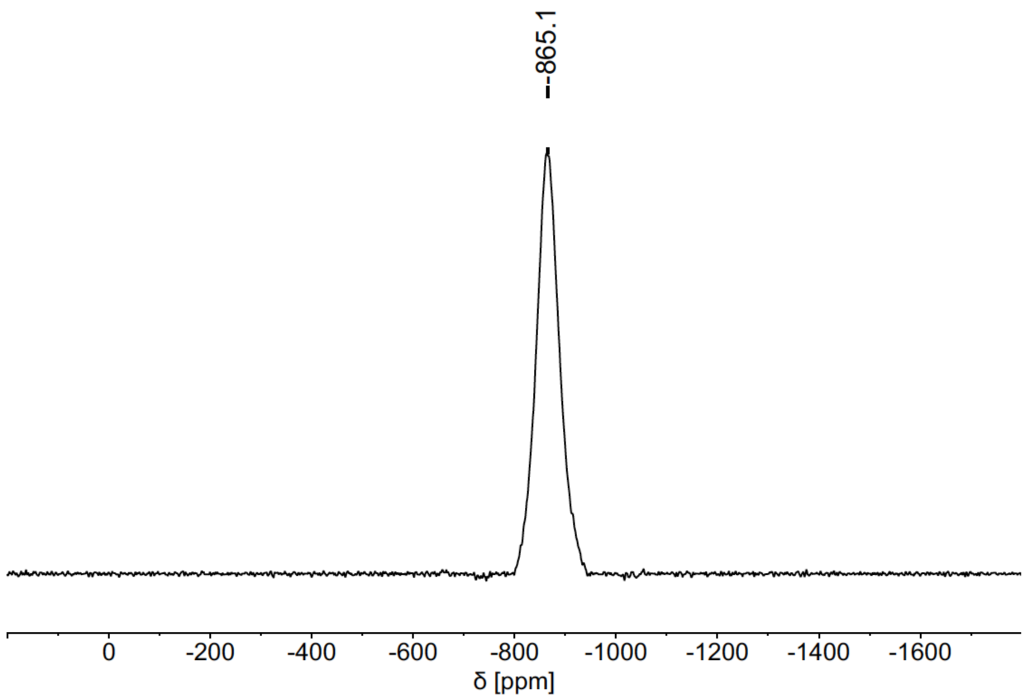

Figure S34: ^{99}Tc NMR spectrum of [Tc(NO)(Cp)(PPh$_3$)(thioxane)](BF$_4$) in CD$_2$Cl$_2$.

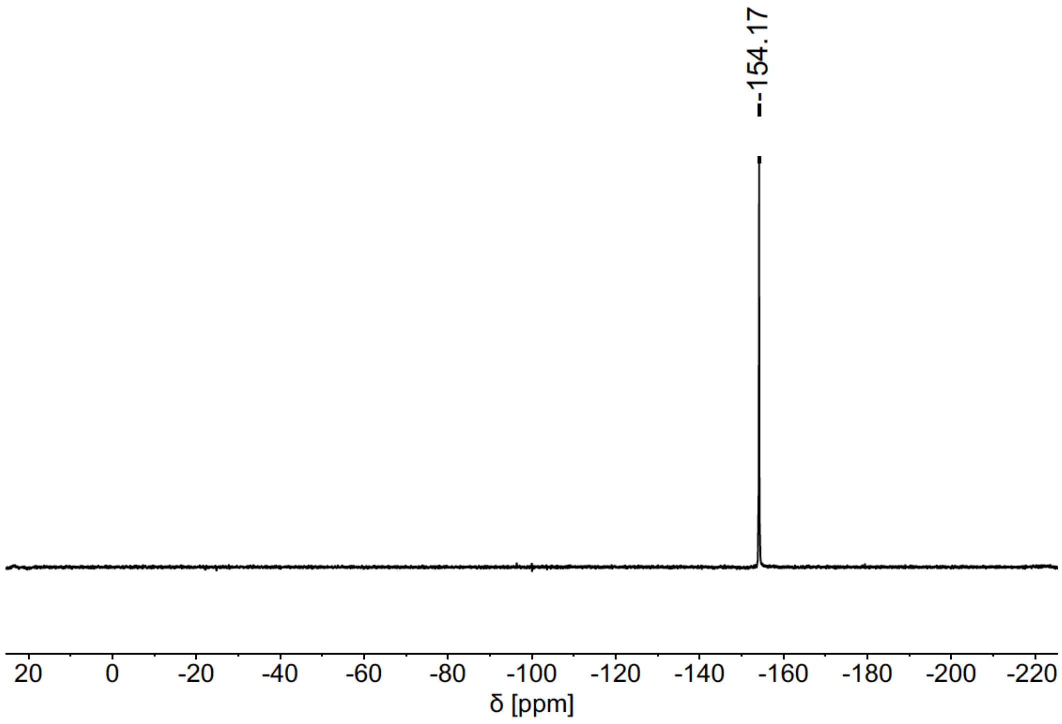

Figure S35: ^{19}F NMR spectrum of [Tc(NO)(Cp)(PPh$_3$)(thioxane)](BF$_4$) in CD$_2$Cl$_2$.

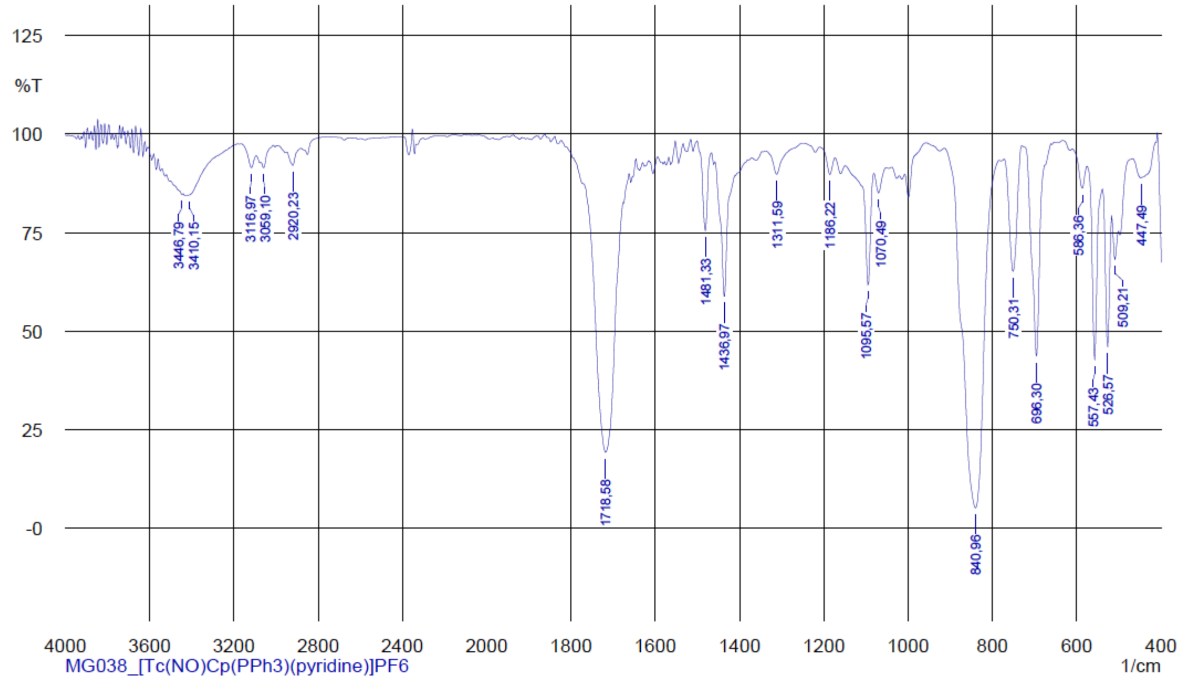

Figure S36: IR (KBr) spectrum of [Tc(NO)(Cp)(PPh_3)(py)](PF_6).

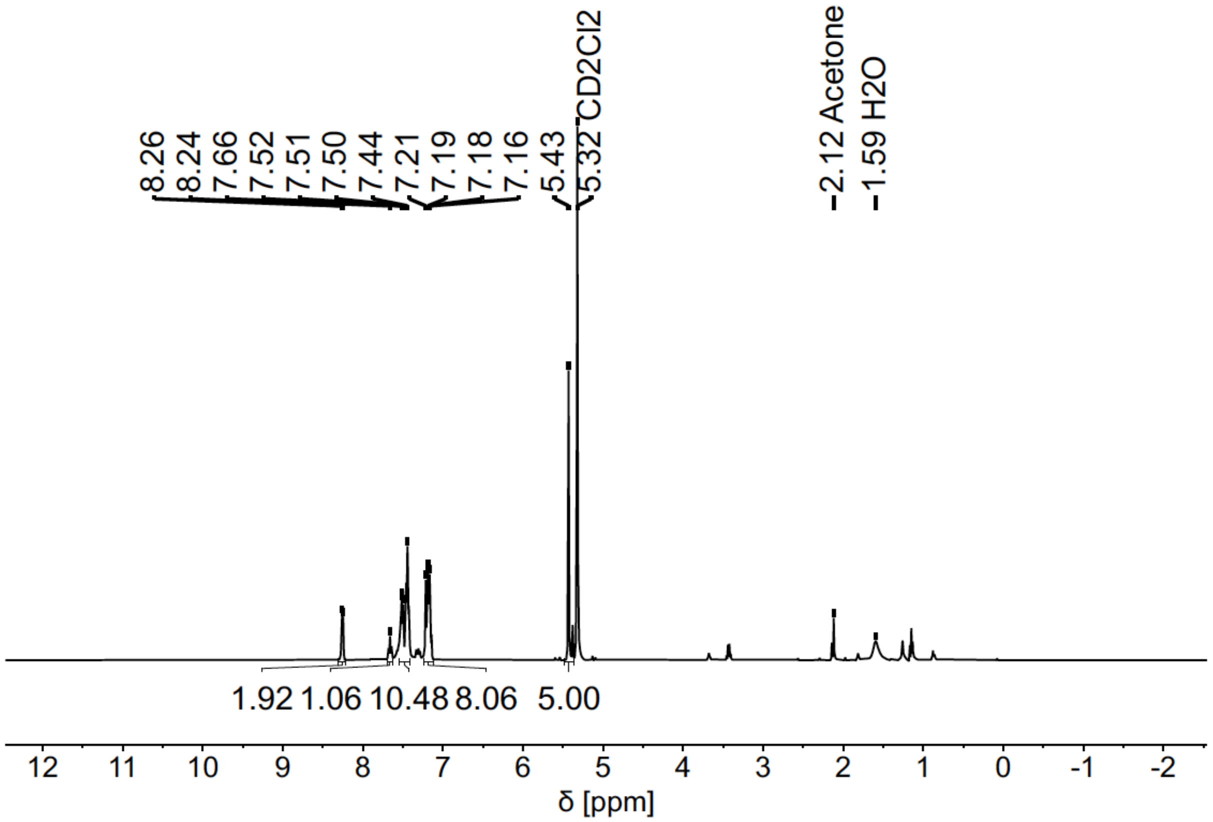

Figure S37: ^{1}H NMR spectrum of [Tc(NO)(Cp)(PPh_3)(py)](PF_6) in $CDCl_3$. Identified solvents are annotated.

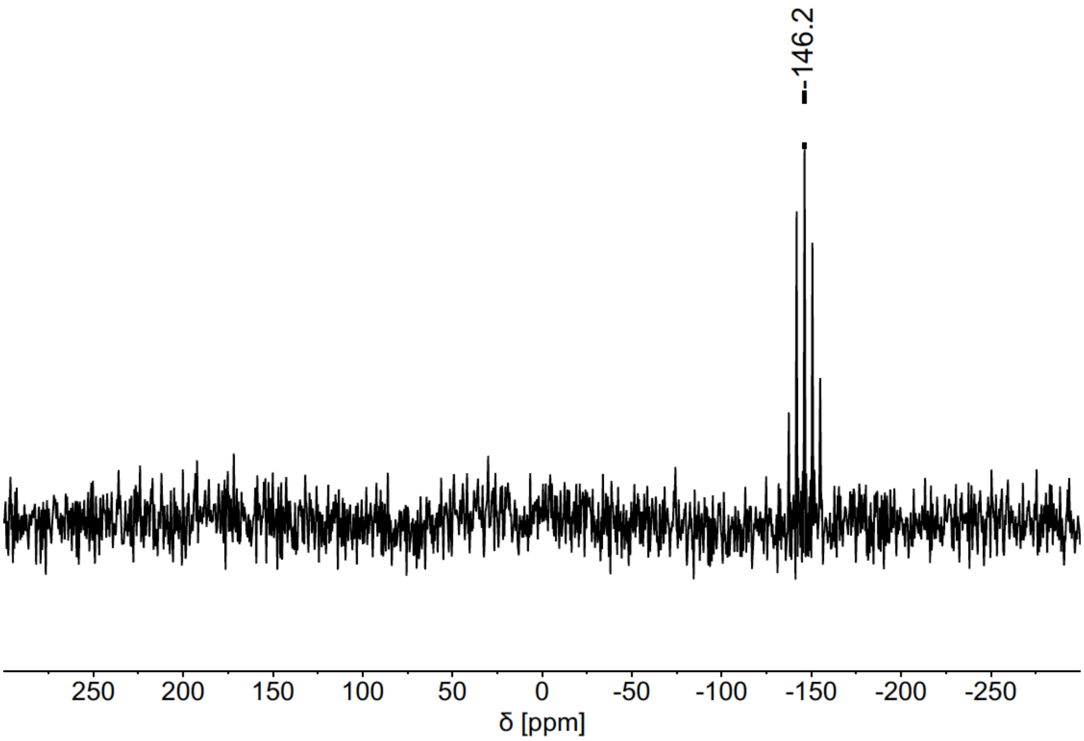

Figure S38: $^{31}P\{^{1}H\}$ NMR spectrum of $[Tc(NO)(Cp)(PPh_3)(py)](PF_6)$ in $CDCl_3$.

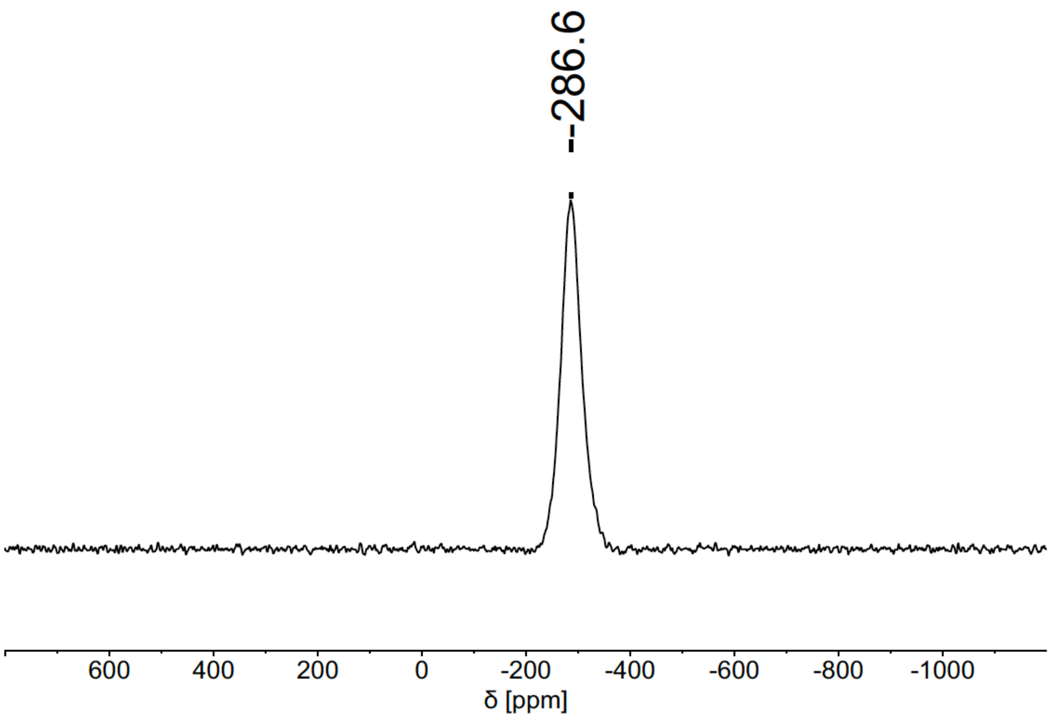

Figure S39: ^{99}Tc NMR spectrum of $[Tc(NO)(Cp)(PPh_3)(py)](PF_6)$ in $CDCl_3$.

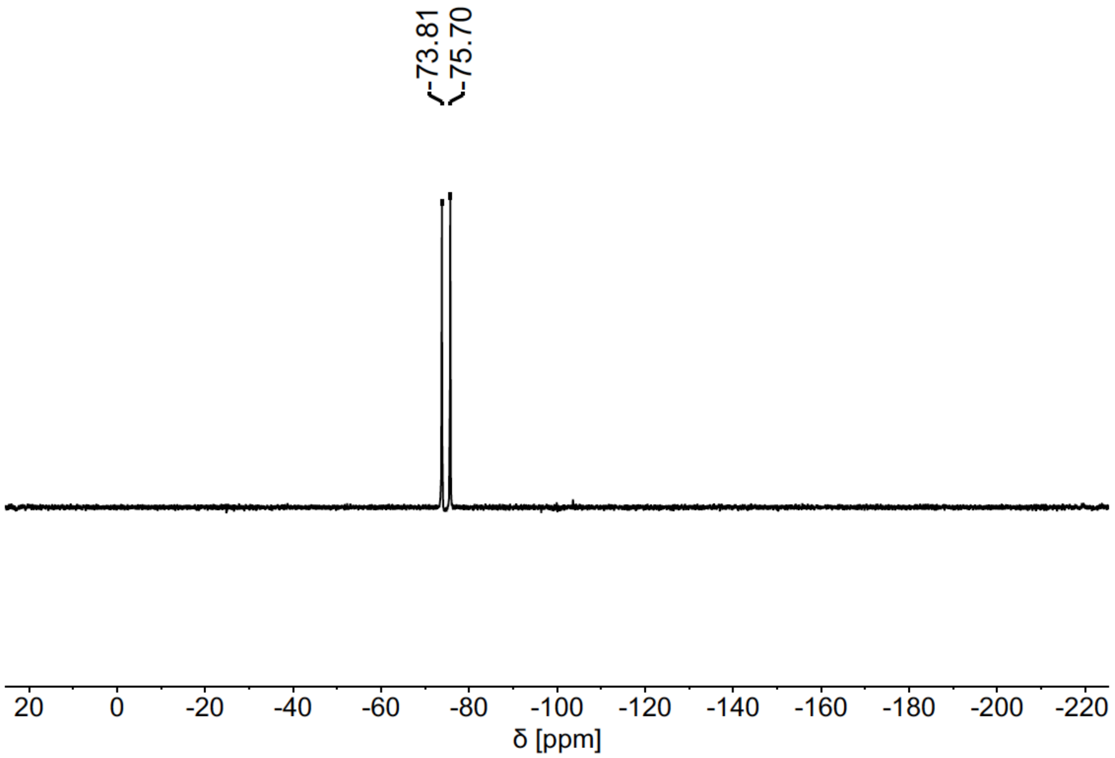

Figure S40: ^{19}F NMR spectrum of [Tc(NO)(Cp)(PPh_3)(py)](PF_6) in $CDCl_3$.

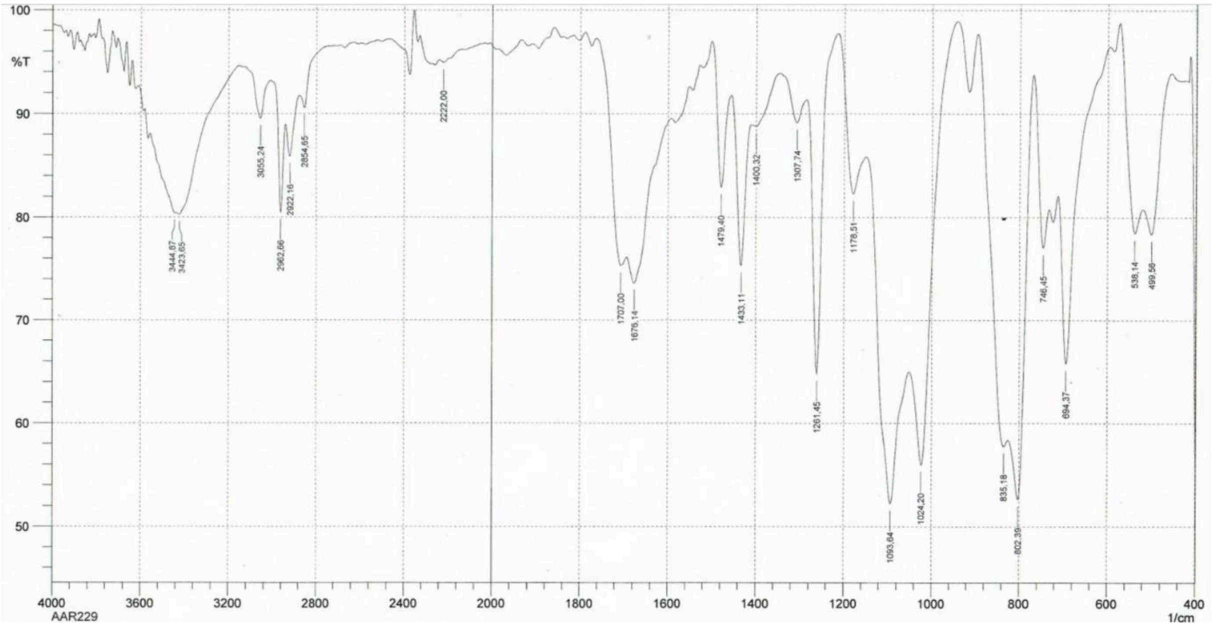

Figure S41: IR (KBr) spectrum of [Tc(NO)(Cp^{Me})(PPh_3)Cl].

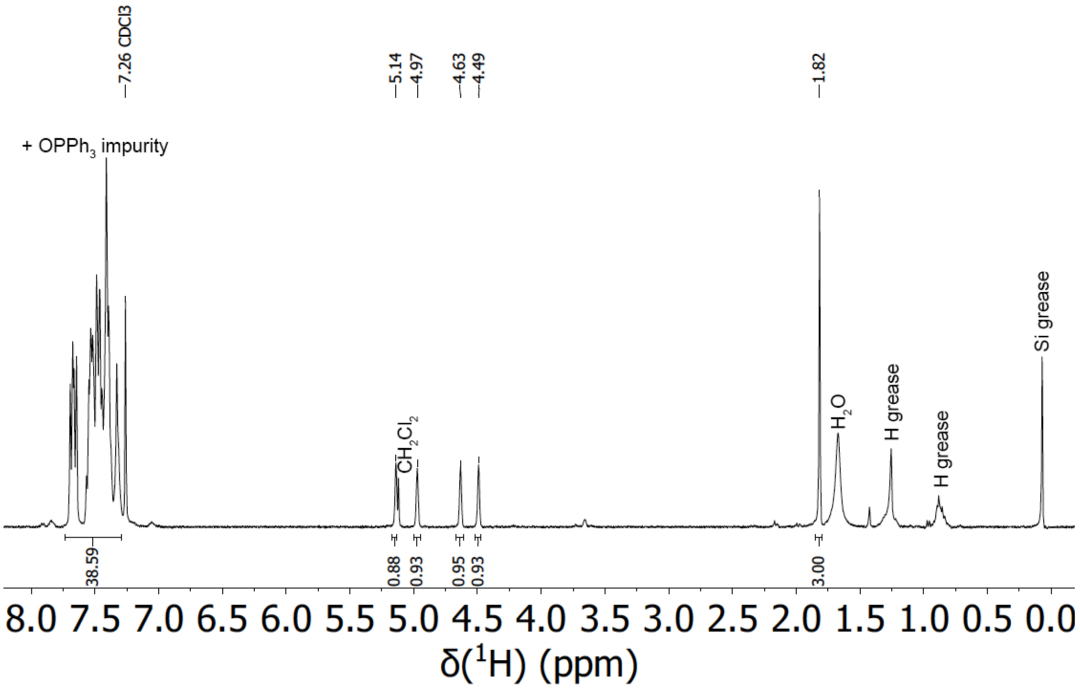

Figure S42: ^{1}H NMR spectrum of [Tc(NO)(Cp^{Me})(PPh_3)Cl] in $CDCl_3$. Identified impurities and solvents are annotated.

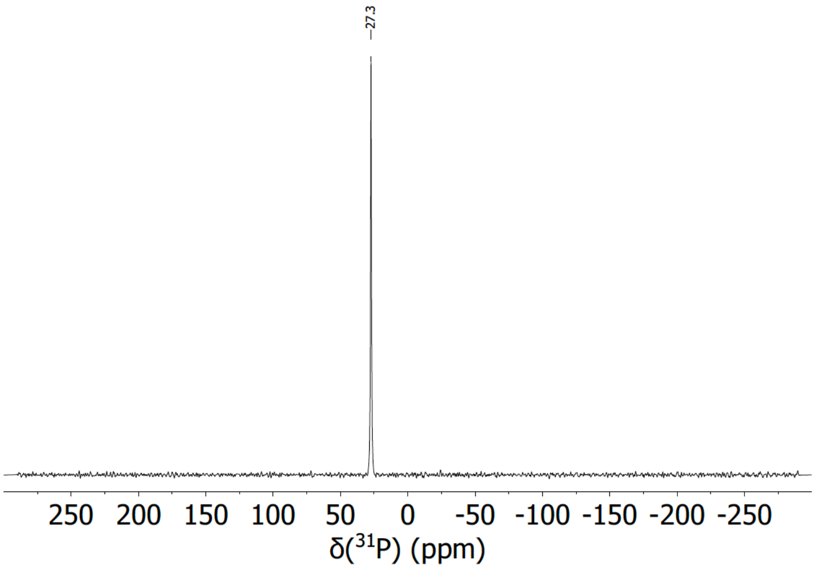

Figure S43: $^{31}P\{^{1}H\}$ NMR spectrum of [Tc(NO)(Cp^{Me})(PPh_3)Cl] in $CDCl_3$. The observed resonance is ambiguously assigned to the bound PPh3 ligand as no other (not even a very broad one) was observed; the narrow resonance would be in accordance with the narrow resonance observed for the pyridine derivative but should not be overvalued as the real resonance could be broad enough to vanish leaving only some OPPh3 impurities resonance.

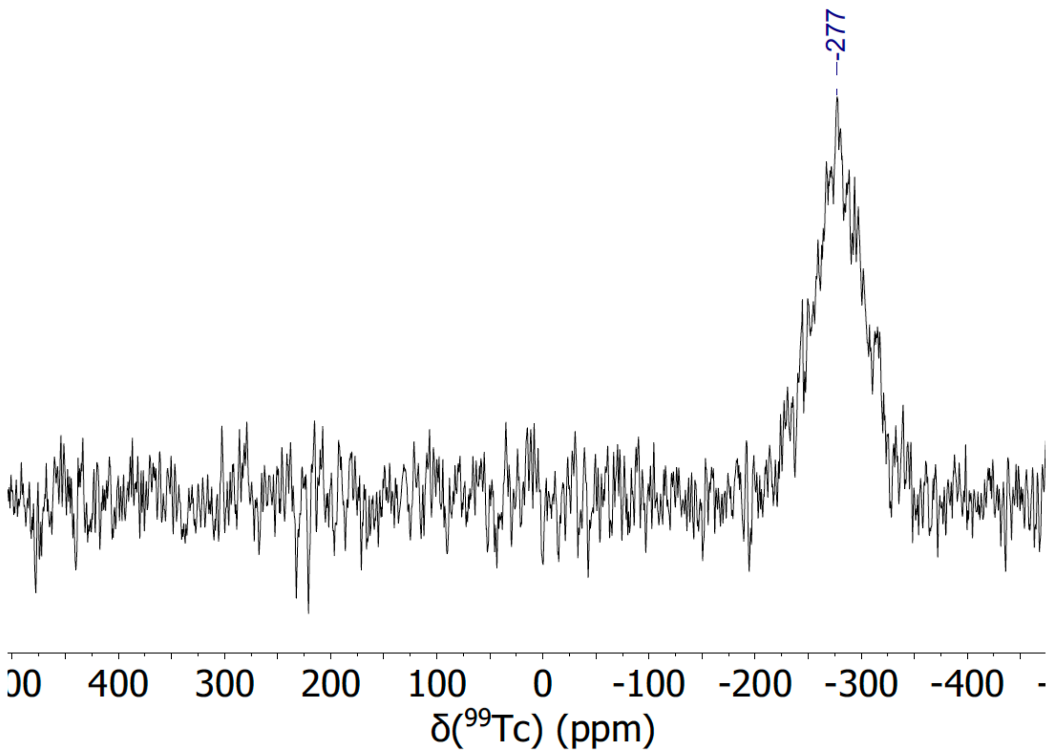

Figure S44: ^{99}Tc NMR spectrum of [Tc(NO)(Cp^{Me})(PPh$_3$)Cl] in $CDCl_3$.

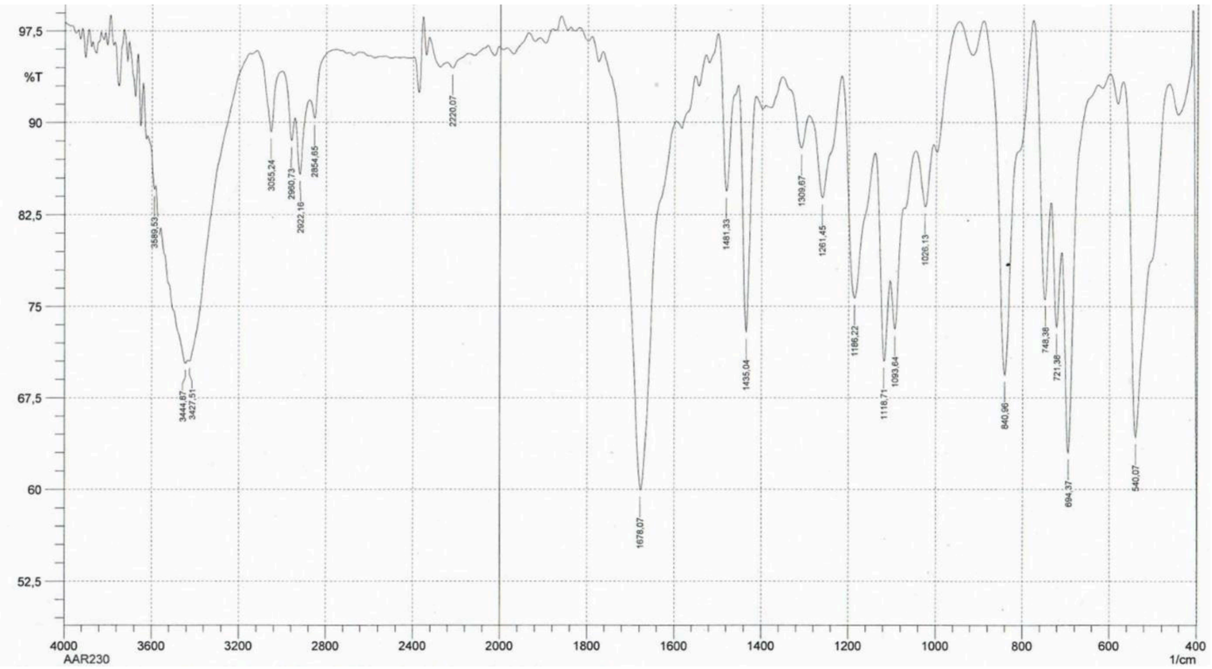

Figure S45: IR (KBr) spectrum of [Tc(NO)(Cp^{Me})(PPh$_3$)(py)](PF$_6$).

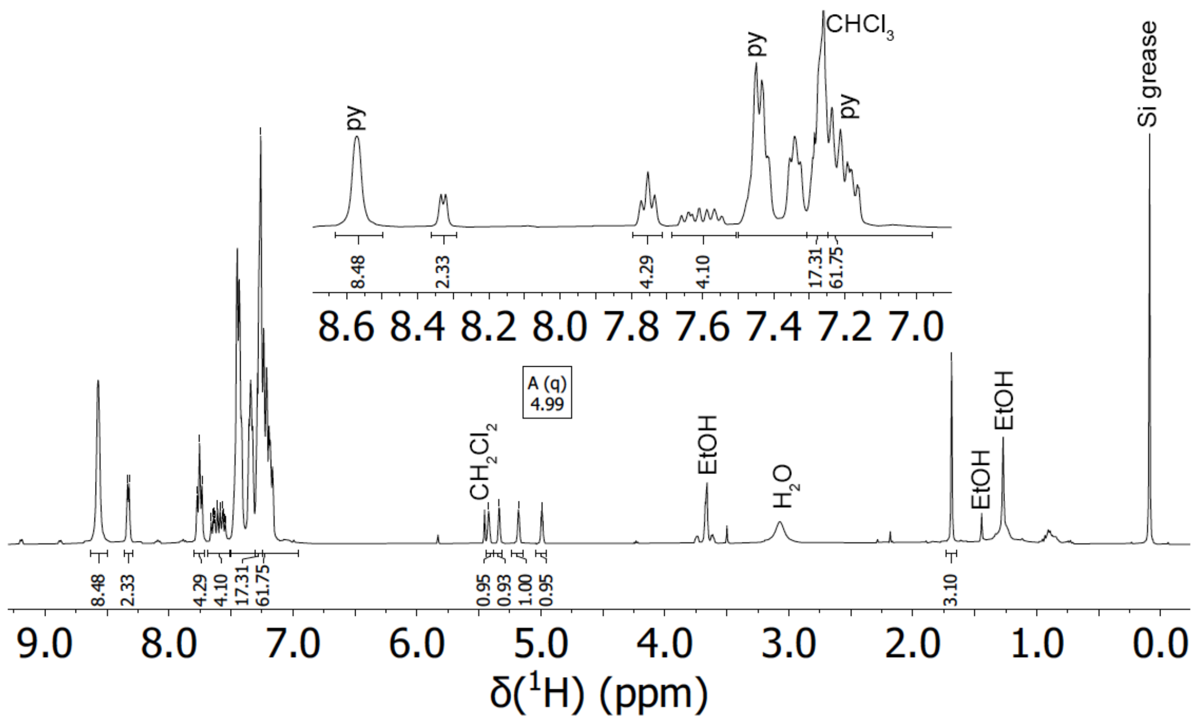

Figure S46: ^{1}H NMR spectrum of $[Tc(NO)(Cp^{Me})(PPh_3)(py)](PF_6)$ in $CDCl_3$. Identified impurities and solvents are annotated.

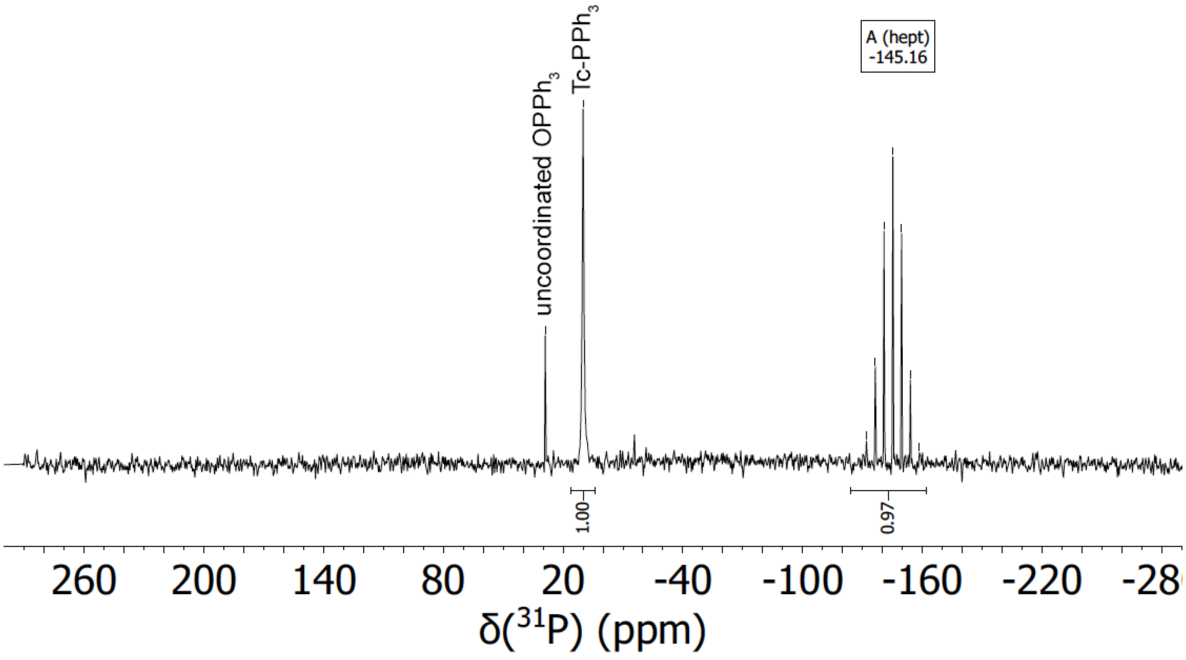

Figure S47: $^{31}P\{^{1}H\}$ NMR spectrum of $[Tc(NO)(Cp^{Me})(PPh_3)(py)](PF_6)$ in $CDCl_3$. Identified impurities are annotated. An uncommonly large exponential apodization function for ^{31}P NMR was applied (20 Hz) after truncation of the FID at 5k points and zero-filling to the original 256k points to improve the interpretability of the somewhat broader resonance of the coordinated PPh_3 ligand.

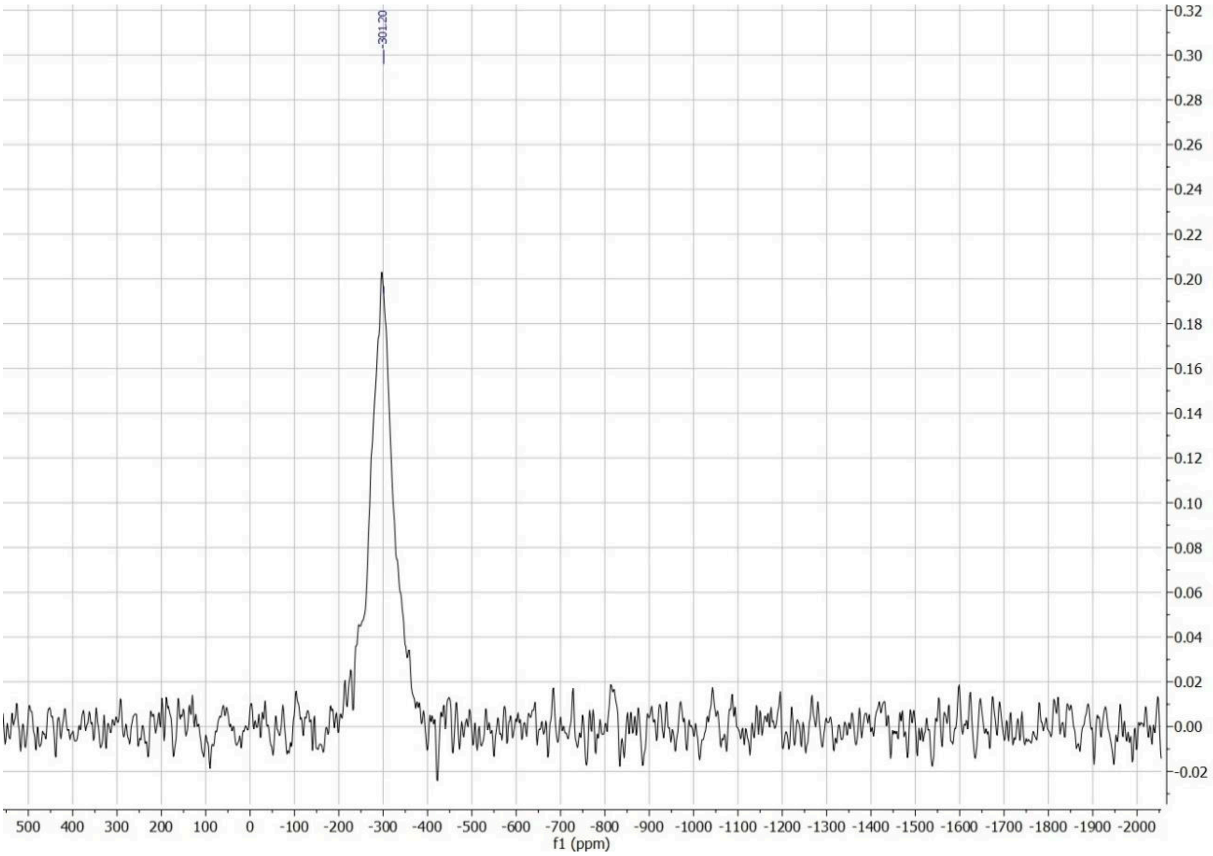

Figure S48: ^{99}Tc NMR spectrum of $[Tc(NO)(Cp^{Me})(PPh_3)(py)](PF_6)$ in $CDCl_3$.

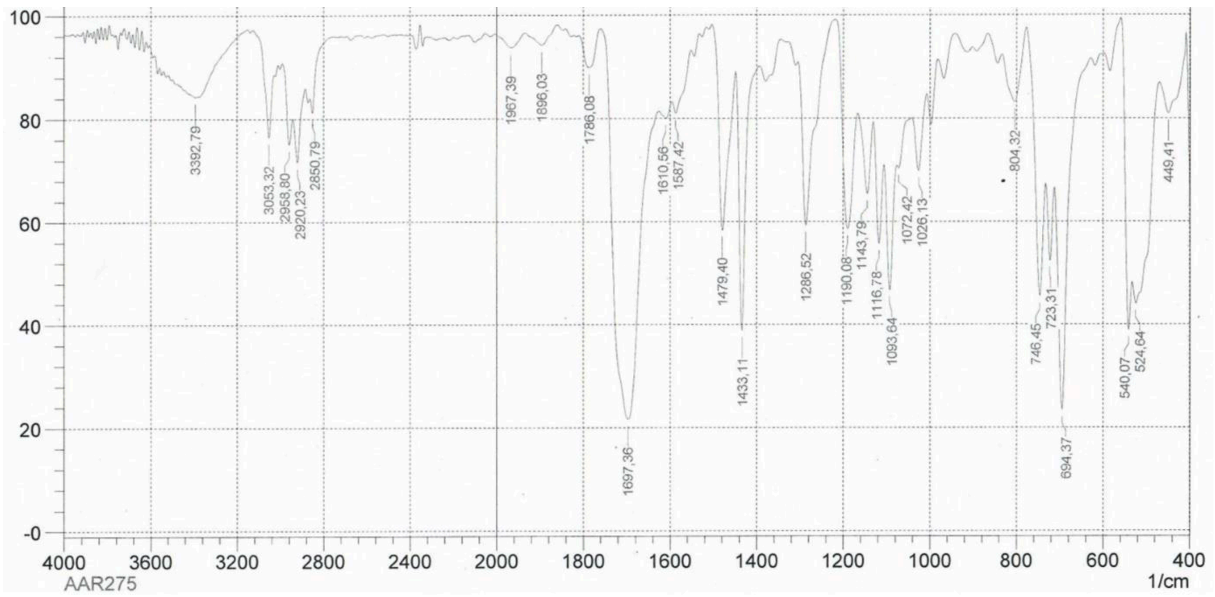

Figure S49: IR (KBr) spectrum of $[Tc(NO)(Cp^{COOMe})(PPh_3)Cl]$.

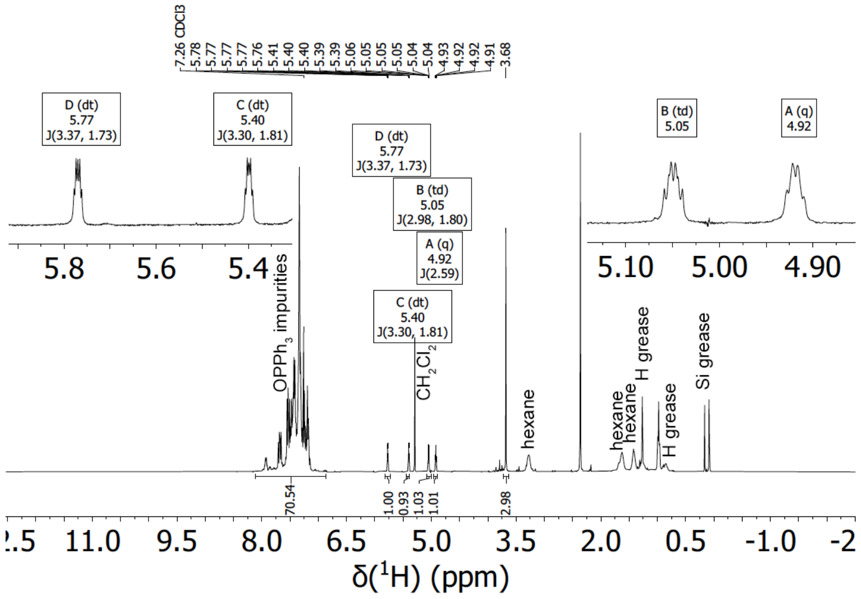

Figure S50: ^{1}H NMR spectrum of [Tc(NO)(CpCOOMe)(PPh_3)Cl] in $CDCl_3$. Identified impurities and solvents are annotated.

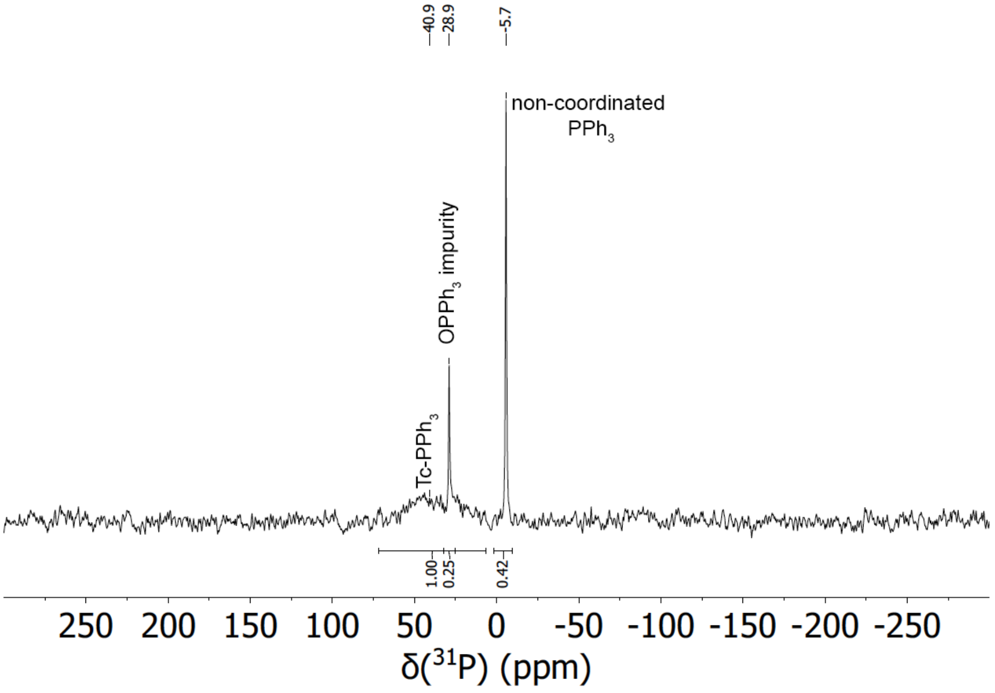

Figure S51: ^{31}P{^{1}H} NMR spectrum of [Tc(NO)(CpCOOMe)(PPh_3)Cl] in $CDCl_3$. Identified are annotated. An uncommonly large exponential apodization function for ^{31}P NMR was applied (100 Hz) after truncation of the FID at 5k points and zero-filling to the original 256k points to improve the interpretability of the very broad resonance of the coordinated PPh_3 ligand.

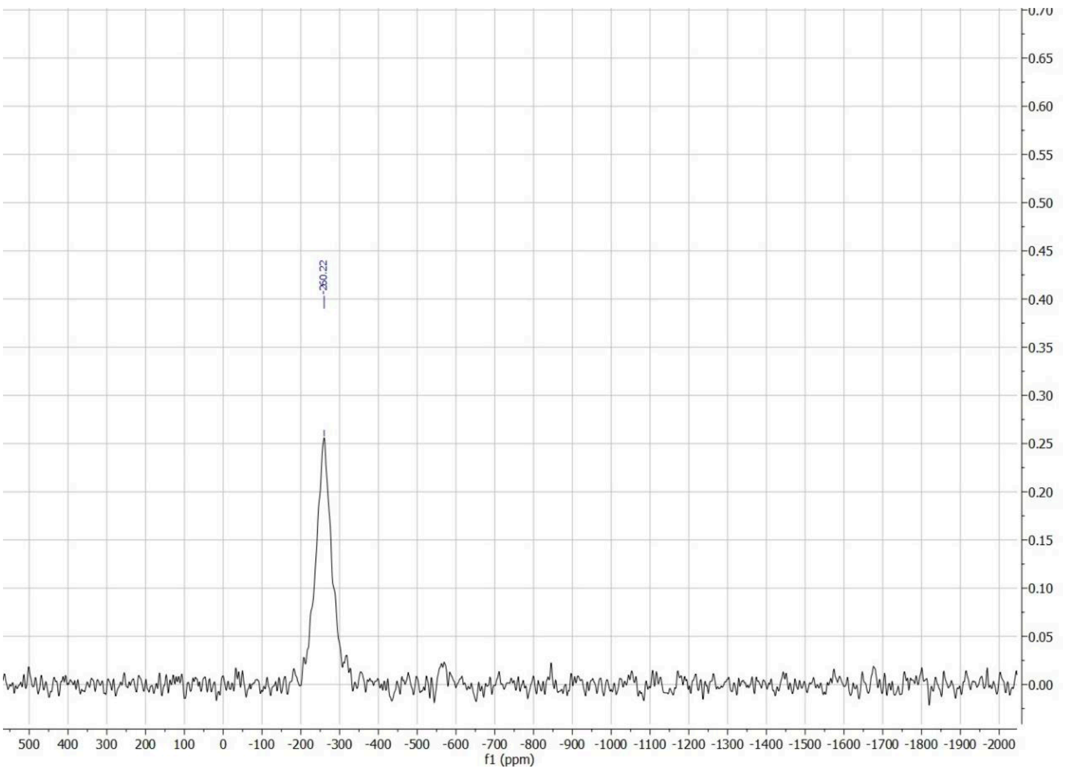

Figure S52: ^{99}Tc NMR spectrum of $[Tc(NO)(CpCOOMe)(PPh_3)Cl]$ in $CDCl_3$.

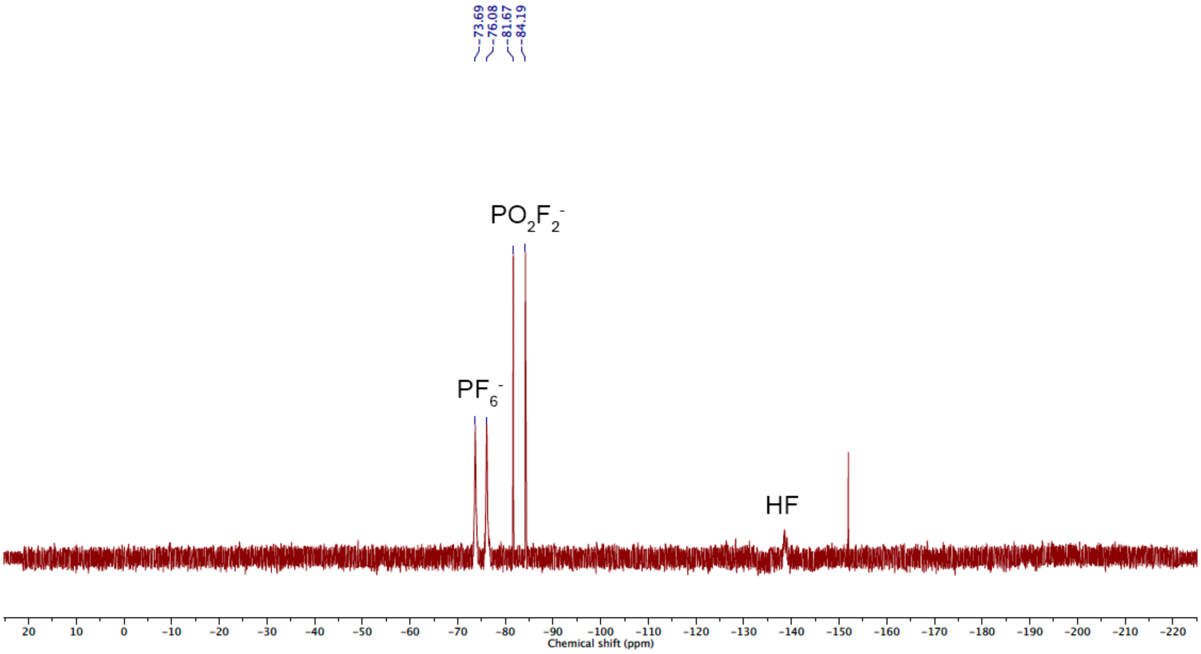

Figure S53: ^{19}F NMR spectrum of a reaction mixture containing PF_6^- anions together with H_2O, MeOH and metal ions, showing the gradual degradation of hexafluorophosphate under formation of oxyfluorides and HF.

Table S9. ^{99}Tc NMR chemical shifts and line widths of $[Tc(NO)(Cp^{R})(PPh_3)(L)]^{0,+}$ complexes.

Complex	Solvent	δ /ppm	$Δ_{1/2}$ / Hz	Ref.
$[Tc(NO)(Cp)(PPh_3)(OPPh_3)]PF_6$	CD_2Cl_2	254	6231	This paper
$[Tc(NO)(Cp)(PPh_3)$ $(OSO_2CF_3)]$	$CDCl_3$	242	7070	[15]
$[Tc(NO)(Cp)(PPh_3)$ $(OOCCF_3)]$	$CDCl_3$	19	4690	[15]
$[\{Tc(NO)(Cp)(PPh_3)\}_2Cl](PF_6)$	CD_2Cl_2	-220	4150	[16]
$[Tc(NO)(PPh_3)(Cp)Cl]$	$CDCl_3$	-231	7170	[14]
$[Tc(NO)Cl(CpCOOMe)(PPh_3)]$	$CDCl_3$	-260	3350	This paper
$[Tc(NO)(Cp)(PPh_3)(py)]PF_6$	$CDCl_3$	-287	4850	This paper
$[Tc(NO)Cl(CpMe)(PPh_3)]$	$CDCl_3$	-775	4750	This paper
$[Tc(NO)(CpMe)(PPh_3)(py)]PF_6$	$CDCl_3$	-301	4680	This paper
$[Tc(NO)Br(Cp)(PPh_3)]$	$CDCl_3$	-35	6500	[14]
$[Tc(NO)(NCS)(Cp)(PPh_3)]$	toluene	-453	6500	[15]
$[Tc(NO)(Cp)(PPh_3)(CH_3CN)]BF_4$	acetone-d_6	-537	5928	This paper
$[Tc(NO)(Cp)(PPh_3)(CH_3CN)]PF_6$	CD_2Cl_2	-535	5930	This paper
$[Tc(NO)I(Cp)(PPh_3)]$	CD_2Cl_2	-668	4200	[15]
$[Tc(NO)(I_3)(Cp)(PPh_3)]$	CD_2Cl_2	-679	6860	[15]
$[Tc(NO)(Cp)(PPh_3)(SPPh_3)]PF_6$	CD_2Cl_2	-781	5783	This paper
$[Tc(NO)(SCN)(Cp)(PPh_3)]$	$CDCl_3$	-820	6580	[15]
$[Tc(NO)(SCN)(Cp)(PPh_3)]$	toluene	-816	6580	[15]
$[Tc(NO)(Cp)(PPh_3)(S\text{-thioxane})]PF_6$	$CDCl_3$	-871	5400	This paper
$[Tc(NO)(Cp)(PPh_3)(S\text{-thioxane})]BF_4$	acetone	-865	4990	This paper
$[Tc(NO)(Cp)(PPh_3)(SePPh_3)]PF_6$	CD_2Cl_2	-881	4851	This paper
$[Tc(NO)(Ph)(Cp)(PPh_3)]$	$CDCl_3$	-1201	8820	[14]
$[Tc(NO)(Cp)(PPh_3)_2]PF_6$	$CDCl_3$	-1219	6600	[16]
$[Tc(NO)(Cp)(PPh_3)\{C(OMe)C_2H_4PPh_3\}](PF_6)_2$	$CDCl_3$	-1371	3900	[16]
$[Tc(NO)(Cp)(PPh_3)(PMe_3)]PF_6$	$CDCl_3$	-1420	4120	[16]
$[Tc(NO)(Cp)(PPh_3)(CO)]BF_4$	$CDCl_3$	-1753	3900	[14]

Computational Data 1: Gas-Phase

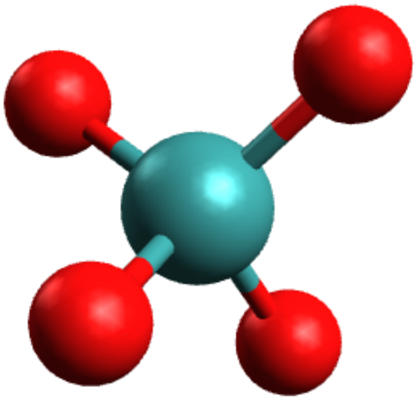

Figure S54: Gas-phase optimized structure of TcO_4^-.

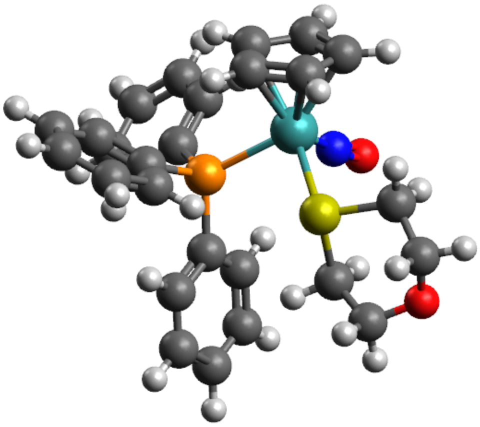

Figure S55: Gas-phase optimized structure of $[Tc(NO)(Cp)(PPh_3)(\textit{S}\text{-thioxane})]^+$.

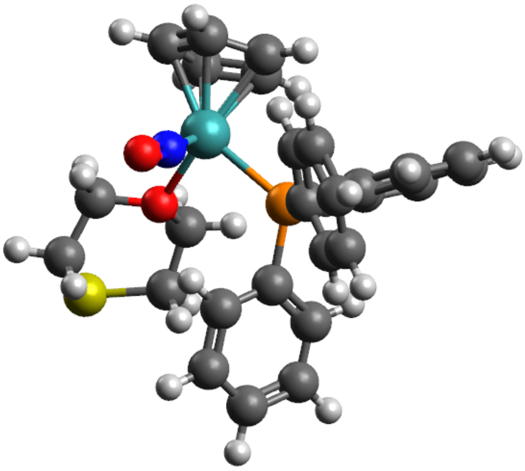

Figure S56: Gas-phase optimized structure of $[Tc(NO)(Cp)(PPh_3)(\textit{O}\text{-thioxane})]^+$.

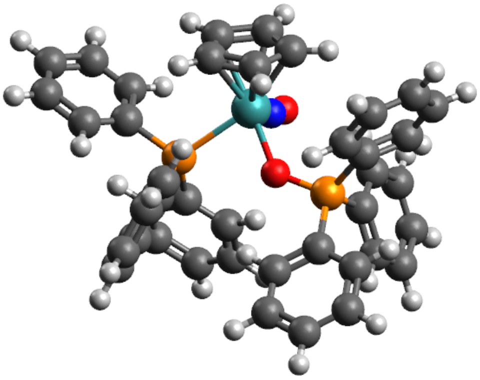

Figure S57: Gas-phase optimized structure of [Tc(NO)(Cp)(PPh_3)($OPPh_3$)]$^+$.

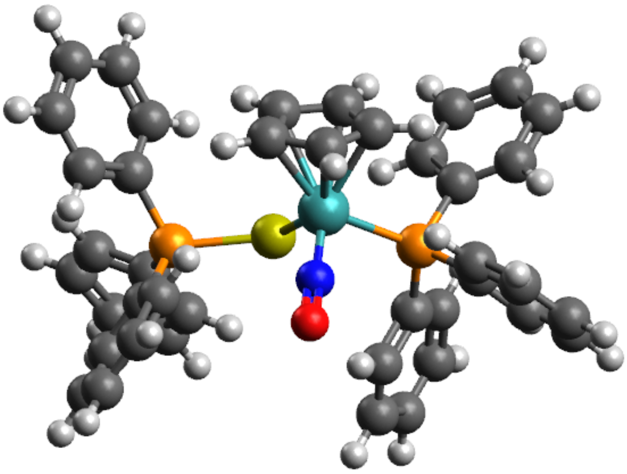

Figure S58: Gas-phase optimized structure of [Tc(NO)(Cp)(PPh_3)($SPPh_3$)]$^+$.

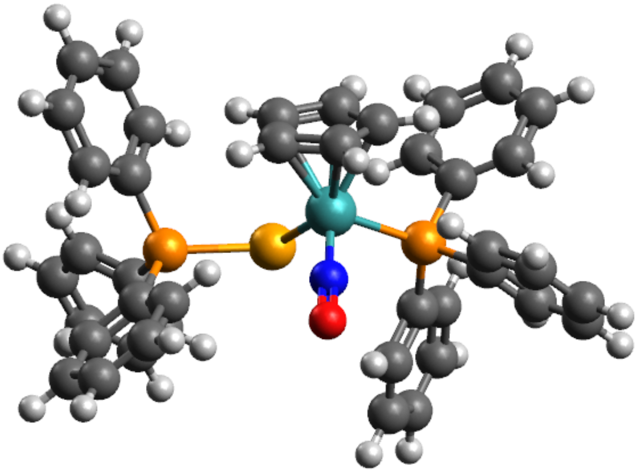

Figure S59: Gas-phase optimized structure of [Tc(NO)(Cp)(PPh_3)($SePPh_3$)]$^+$.

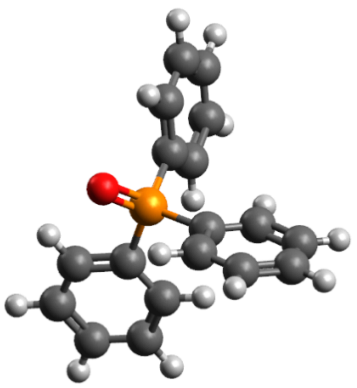

Figure S60: Gas-phase optimized structure of $OPPh_3$.

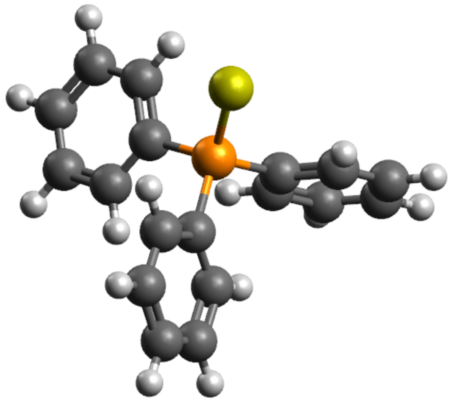

Figure S61: Gas-phase optimized structure of $SPPh_3$.

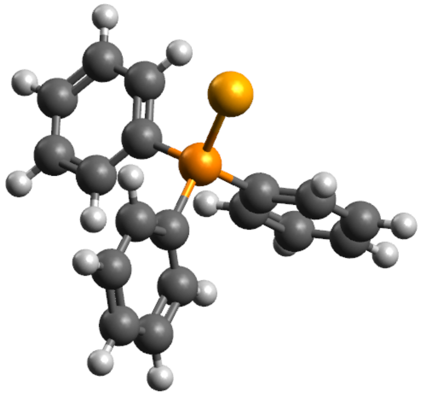

Figure S62: Gas-phase optimized structure of $SePPh_3$.

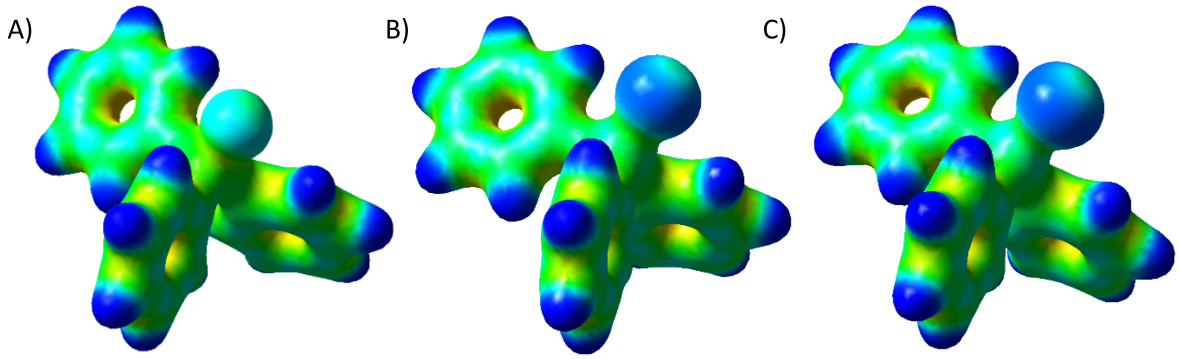

Figure S63: Electron localization function plot for the gas-phase optimized structure of free A) $OPPh_3$, B) $SPPh_3$ and C) $SePPh_3$. Color-scale: blue = 1, green = 0.5 and red = 0.

Table S10: Comparison of the calculated adapted Hirshfeld (ADCH) charges, fuzzy bond orders and experimental NMR chemical shifts in the phosphine chalcogenides and their technetium(I) complexes.

		Complex	free	Δ(free-complex)
ADCH	Tc	-0.15		
	Se	-0.14	-0.37	0.23
	P@Tc	0.35		
	P@Se	0.52	0.44	0.08
Fuzzy bond-order	P-Se	1.12	1.53	0.40
	Tc-Se	1.09		
δ [ppm]	$^{31}P\{^{1}H\}_{Se-P}$	-30		
	^{99}Tc	-881		
ADCH	Tc	-0.12		
	S	-0.22	-0.42	0.20
	P@Tc	0.32		
	P@S	0.54	0.49	0.05
Fuzzy bond-order	P-S	1.07	1.53	0.53
	Tc-S	1.15		
δ [ppm]	$^{31}P\{^{1}H\}_{S-P}$	-50		
	^{99}Tc	-781		
ADCH	Tc	-0.06		
	O	-0.38	-0.55	0.18
	P@Tc	0.28		
	P@O	0.64	0.57	0.06
Fuzzy bond-order	P-O	1.48	0.89	0.42
	Tc-O	1.90		
δ [ppm]	$^{31}P\{^{1}H\}_{O=P}$	-56		
	^{99}Tc	254		

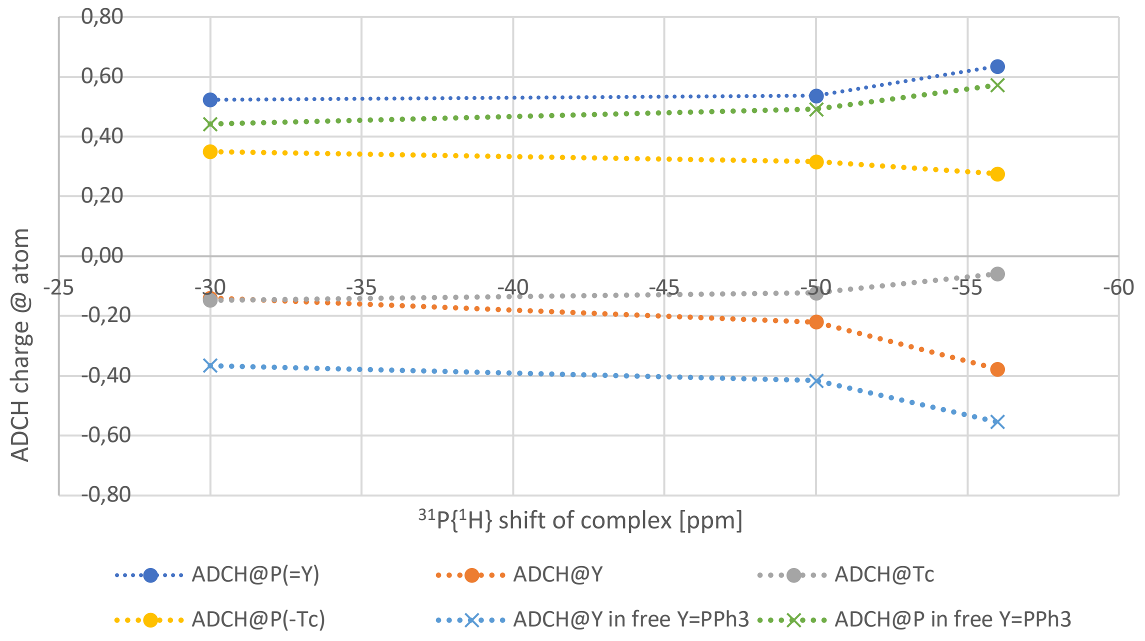

Figure S64: Correlation between ADCH charges and ^{99}Tc chemical shift of the complexes. For the ADCH at technetium and the phosphorus atom in the phosphine chalcogenide ligand, trend lines are provided as they correlate linearly.

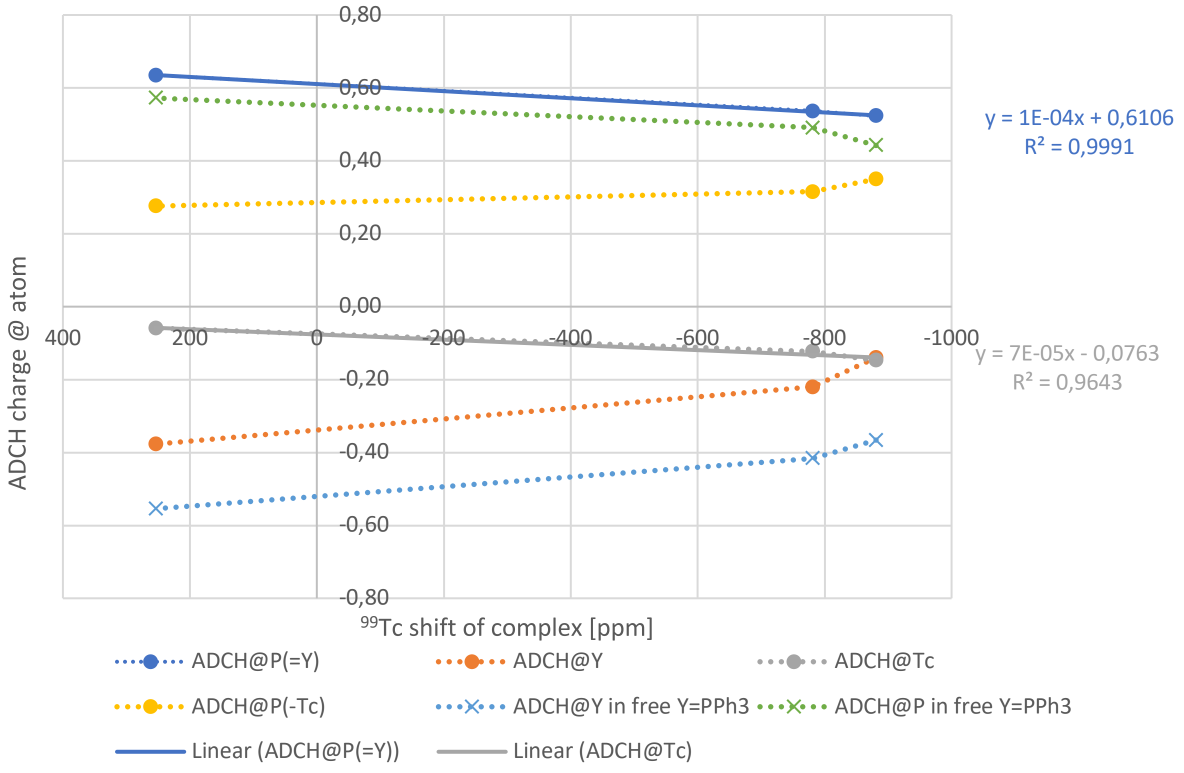

Figure S65: Correlation between ADCH charges and ^{99}Tc chemical shift of the complexes. For the ADCH at technetium and the phosphorus atom in the phosphine chalcogenide ligand, trend lines are provided as they correlate linearly.

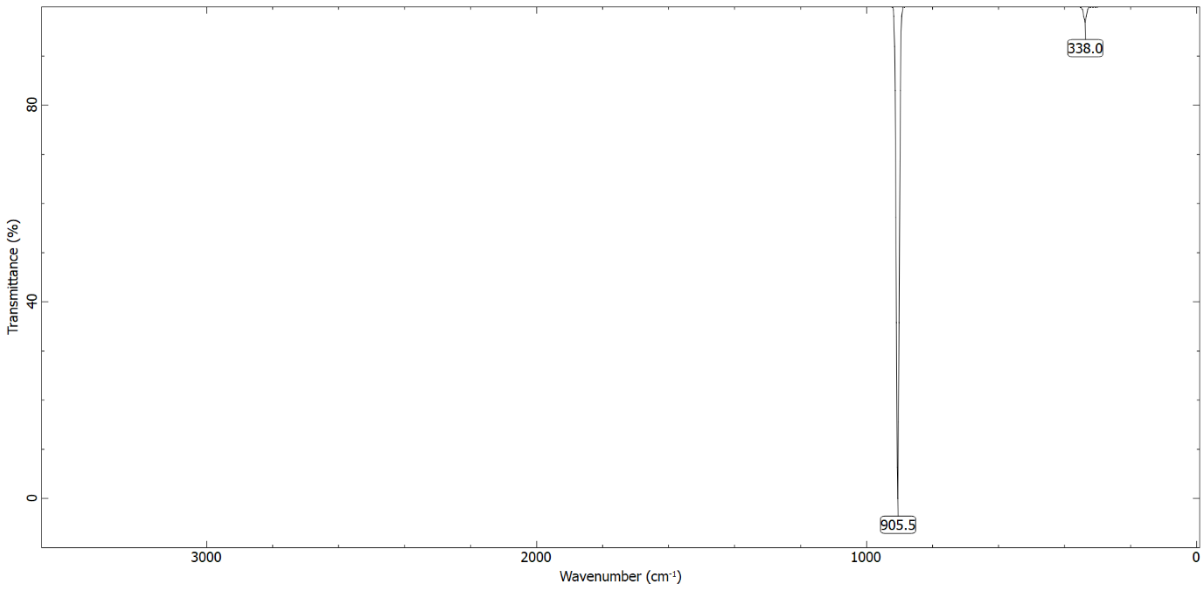

Figure S66: Theoretical IR spectrum (intensity cut-off for peak-labels: 2%) of the gas-phase optimized structure of TcO_4^-.

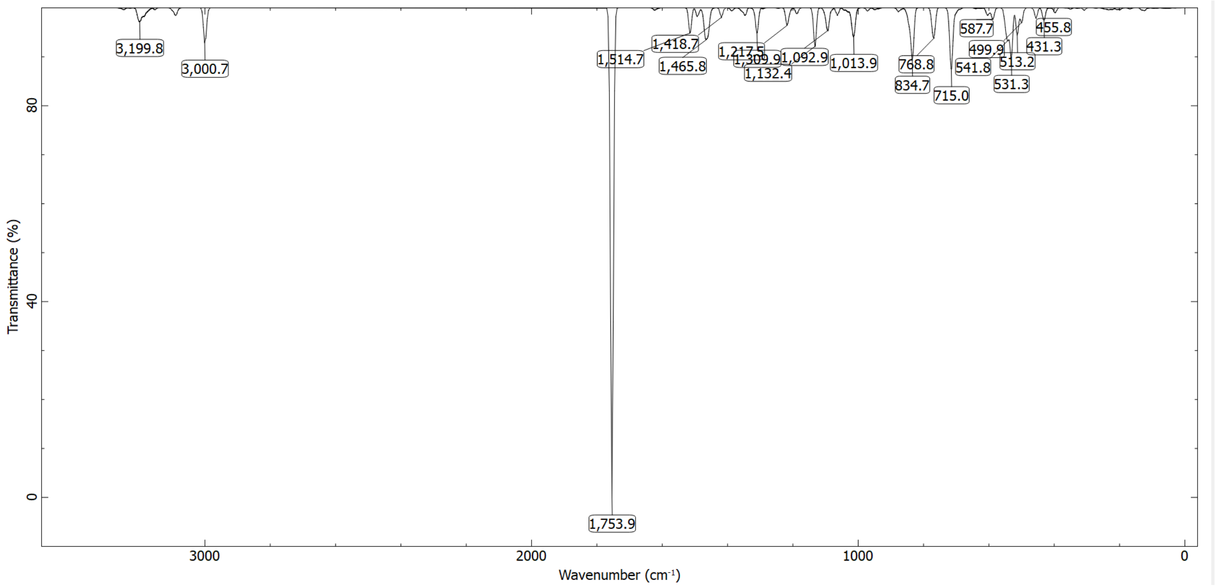

Figure S67: Theoretical IR spectrum (intensity cut-off for peak-labels: 2%) of the gas-phase optimized structure of $[Tc(NO)(Cp)(PPh_3)(S\text{-thioxane})]^+$.

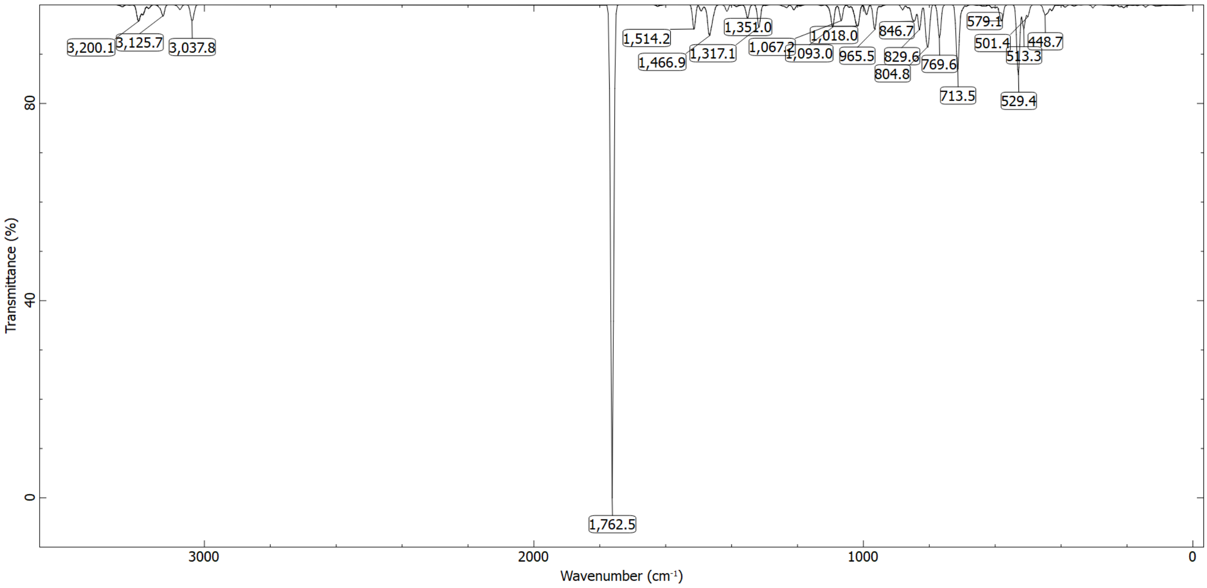

Figure S68: Theoretical IR spectrum (intensity cut-off for peak-labels: 2%) of the gas-phase optimized structure of $[Tc(NO)(Cp)(PPh_3)(\textit{O}\text{-thioxane})]^+$.

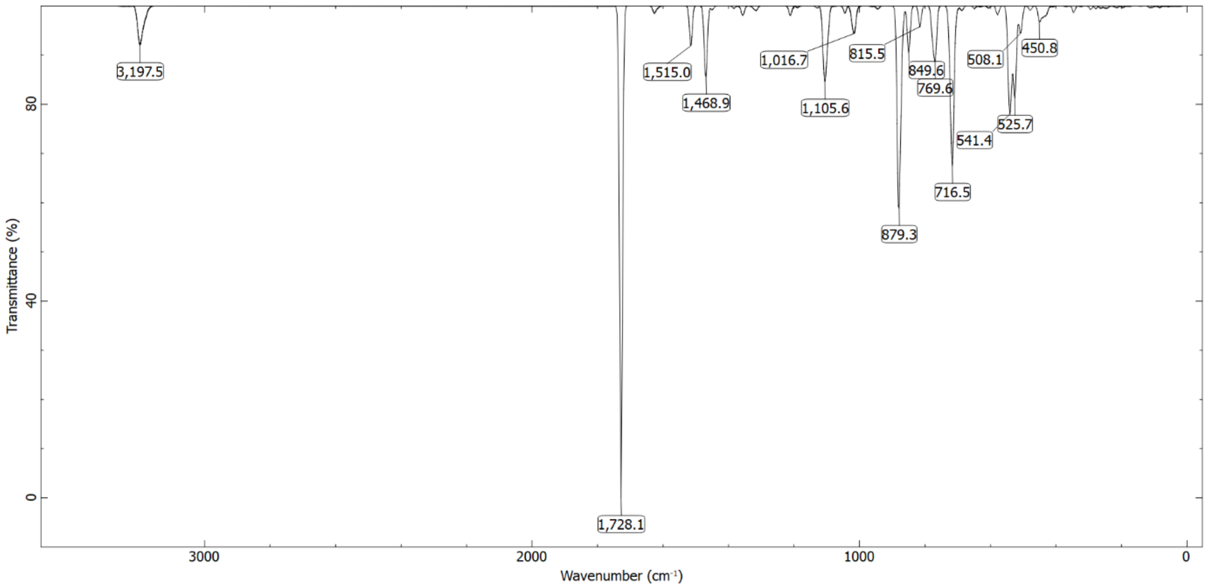

Figure S69: Theoretical IR spectrum (intensity cut-off for peak-labels: 2%) of the gas-phase optimized structure of $[Tc(NO)(Cp)(PPh_3)(OPPh_3)]^+$.

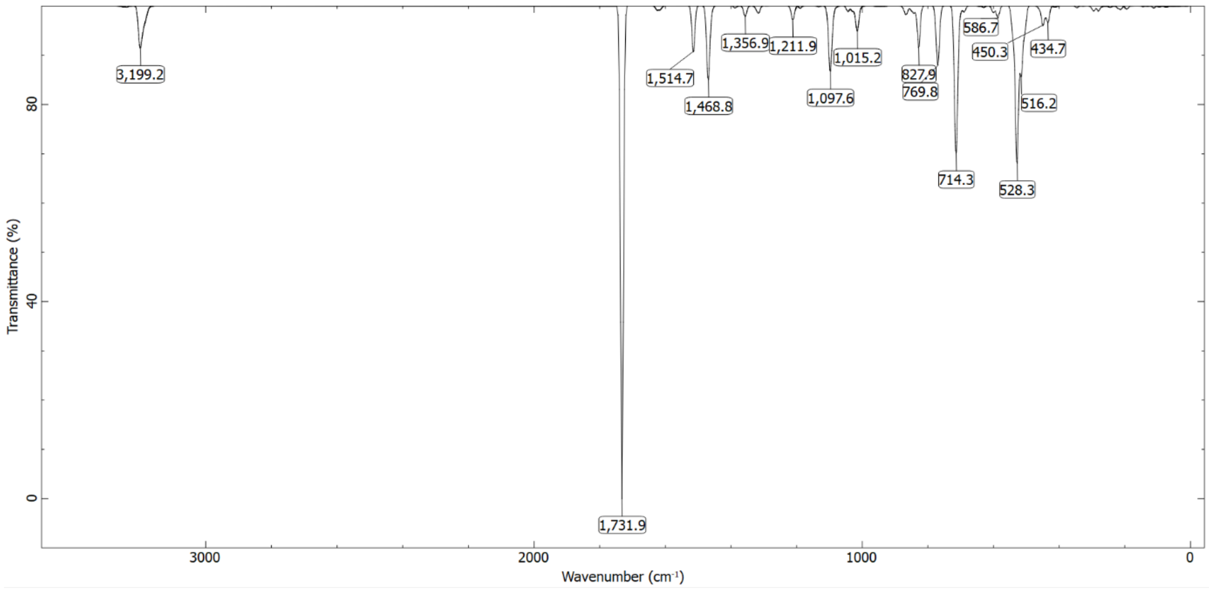

Figure S70: Theoretical IR spectrum (intensity cut-off for peak-labels: 2%) of the gas-phase optimized structure of $[Tc(NO)(Cp)(PPh_3)(SPPh_3)]^+$.

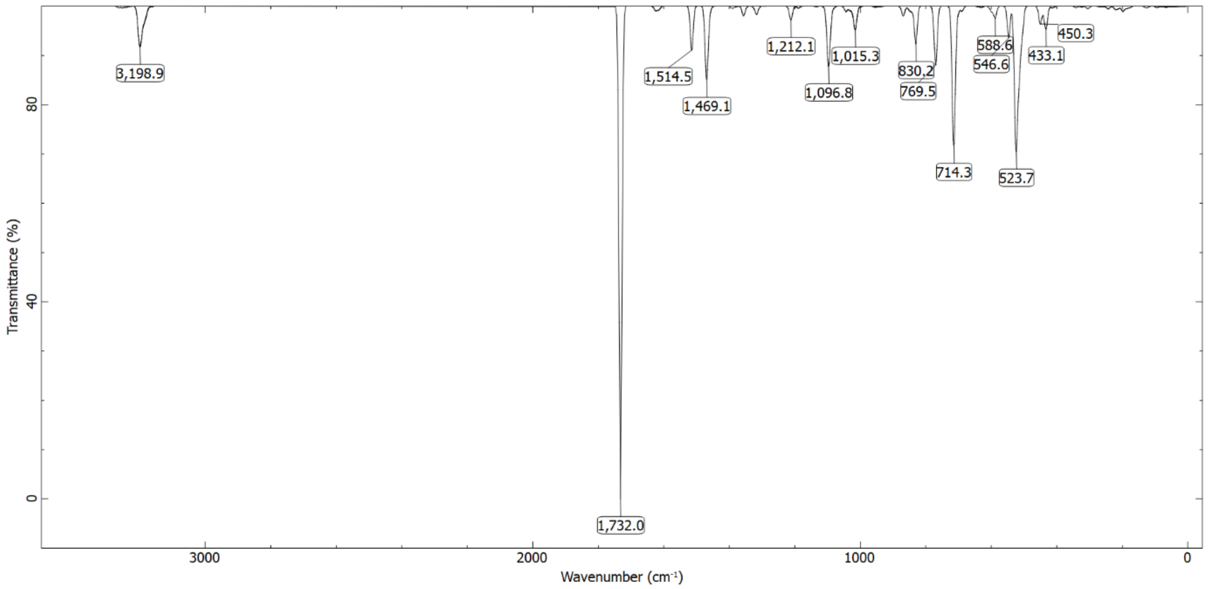

Figure S71: Theoretical IR spectrum (intensity cut-off for peak-labels: 20%) of the gas-phase optimized structure of $[Tc(NO)(Cp)(PPh_3)(SePPh_3)]^+$.

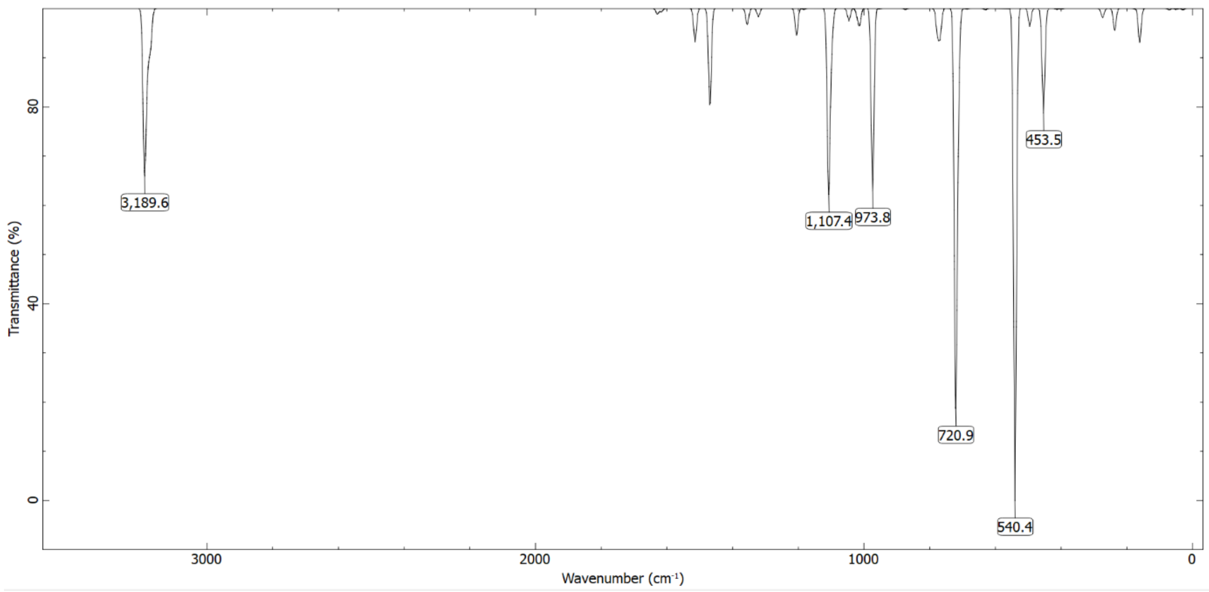

Figure S72: Theoretical IR spectrum (intensity cut-off for peak-labels: 20%) of the gas-phase optimized structure of $OPPh_3$.

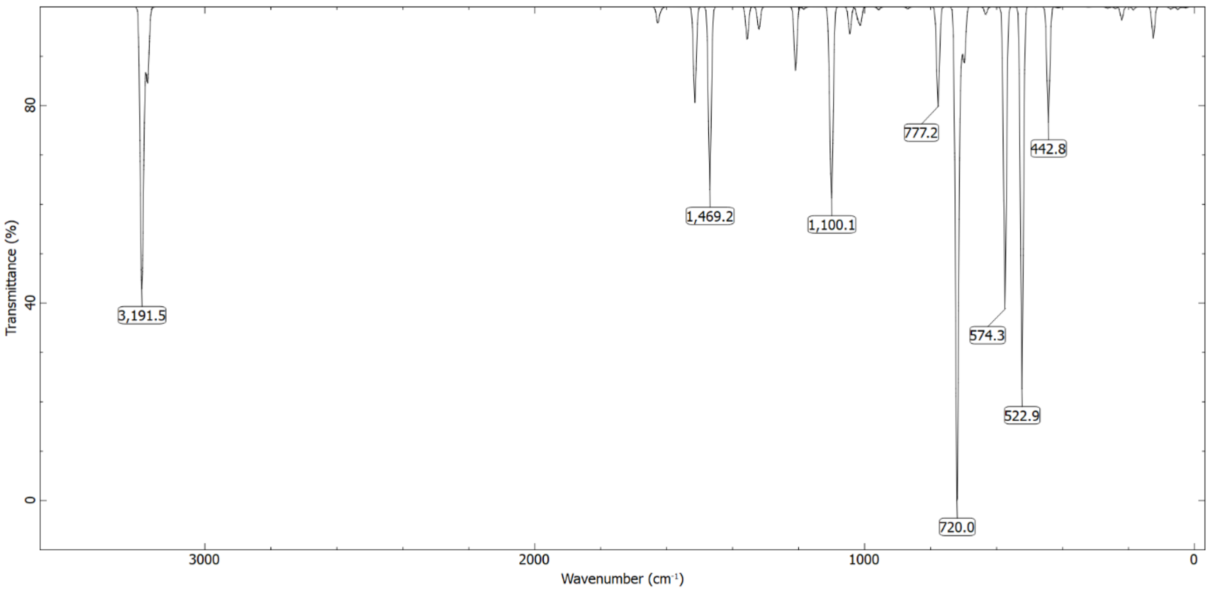

Figure S73: Theoretical IR spectrum (intensity cut-off for peak-labels: 20%) of the gas-phase optimized structure of $SPPh_3$.

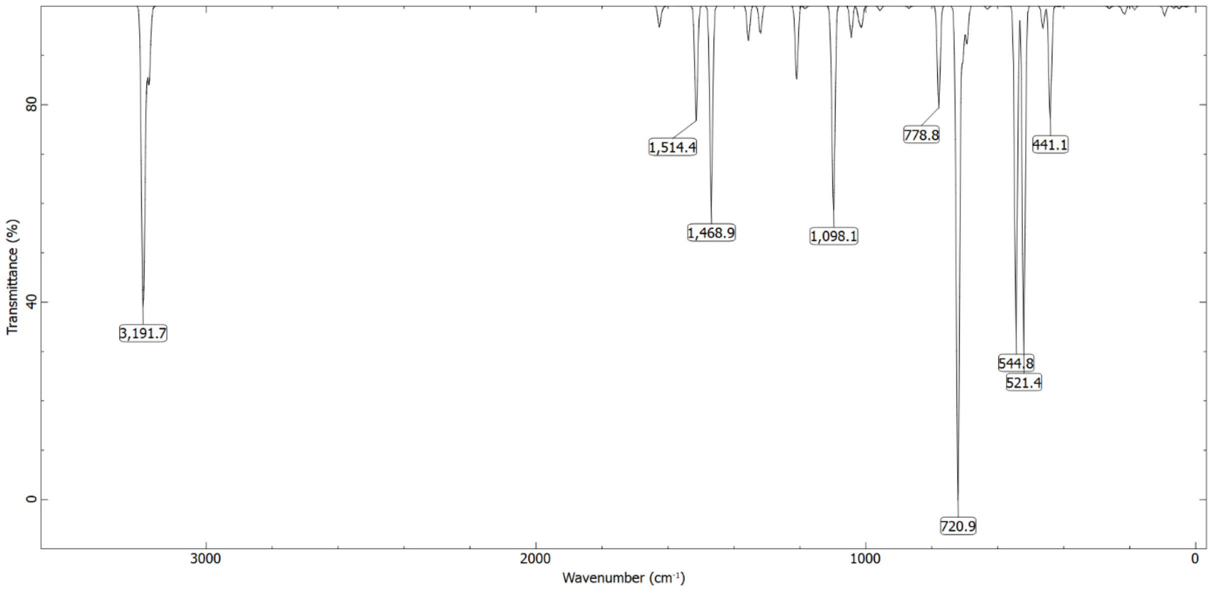

Figure S74: Theoretical IR spectrum (intensity cut-off for peak-labels: 20%) of the gas-phase optimized structure of $SePPh_3$.

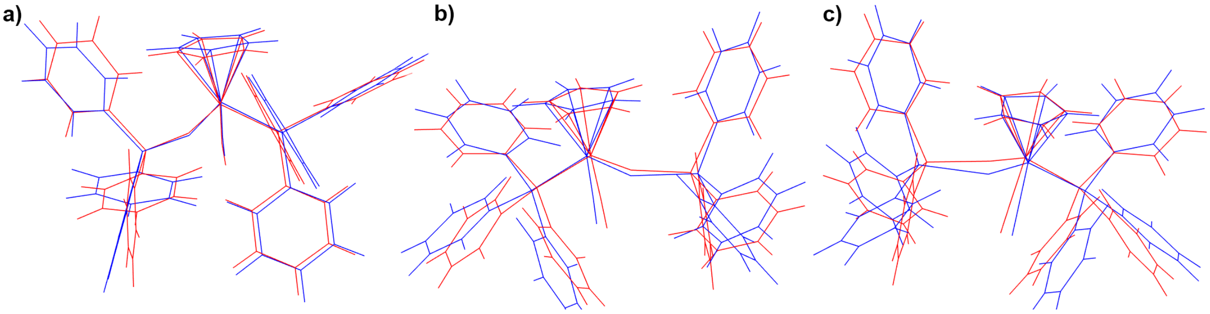

Figure S75: Overlays of the computed gas-phase structures (blue) of a) $[Tc(NO)(Cp)(PPh_3)(OPPh_3)]^+$, b) $[Tc(NO)(Cp)(PPh_3)(SPPh_3)]^+$ and c) $[Tc(NO)(Cp)(PPh_3)(SePPh_3)]^+$ with the corresponding structures derived from the X-ray diffraction data (red).

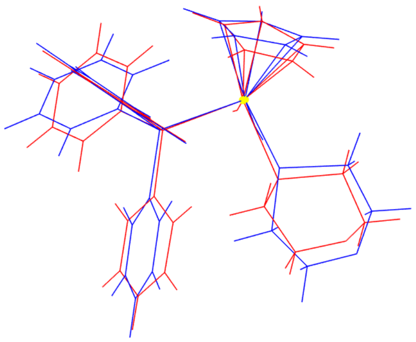

Figure S76: Overlay of the computed gas-phase structures (blue) of $[Tc(NO)(Cp)(PPh_3)(thioxane)]^+$ with the corresponding structures derived from the X-ray diffraction data (red).

Computational Data 2: Implicit Solvation Model

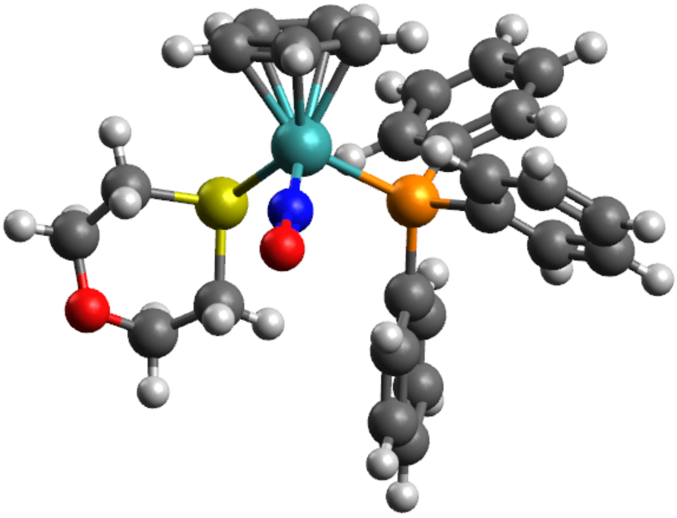

Figure S77: Optimized structure of $[Tc(NO)(Cp)(PPh_3)(\textit{S}\text{-thioxane})]^+$ in THF solution.

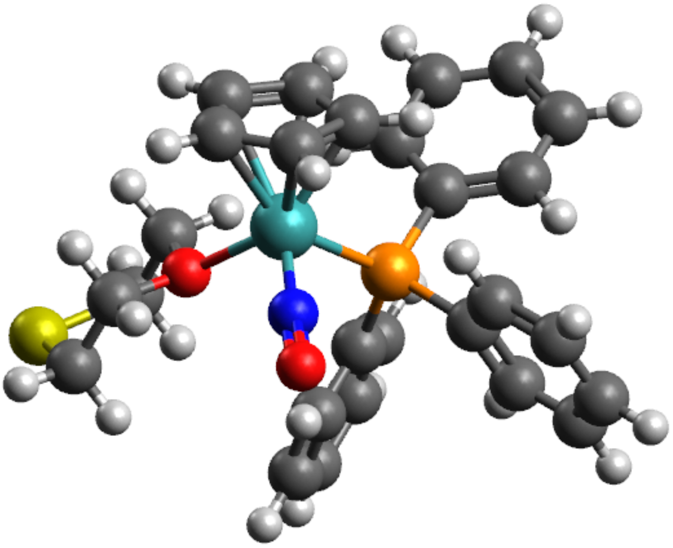

Figure S78: Optimized structure of $[Tc(NO)(Cp)(PPh_3)(\textit{O}\text{-thioxane})]^+$ in THF solution.

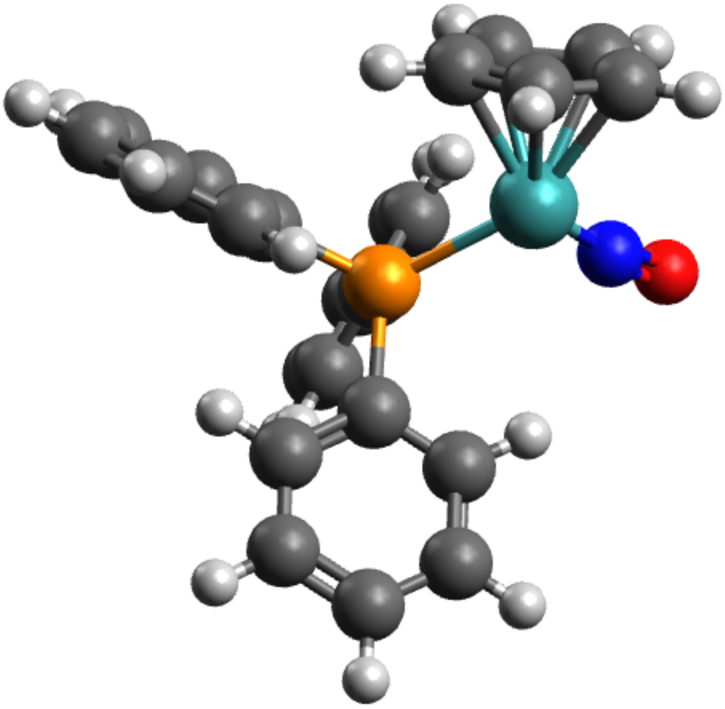

Figure S79: Optimized structure of $[Tc(NO)(Cp)(PPh_3)]^+$ in THF solution.

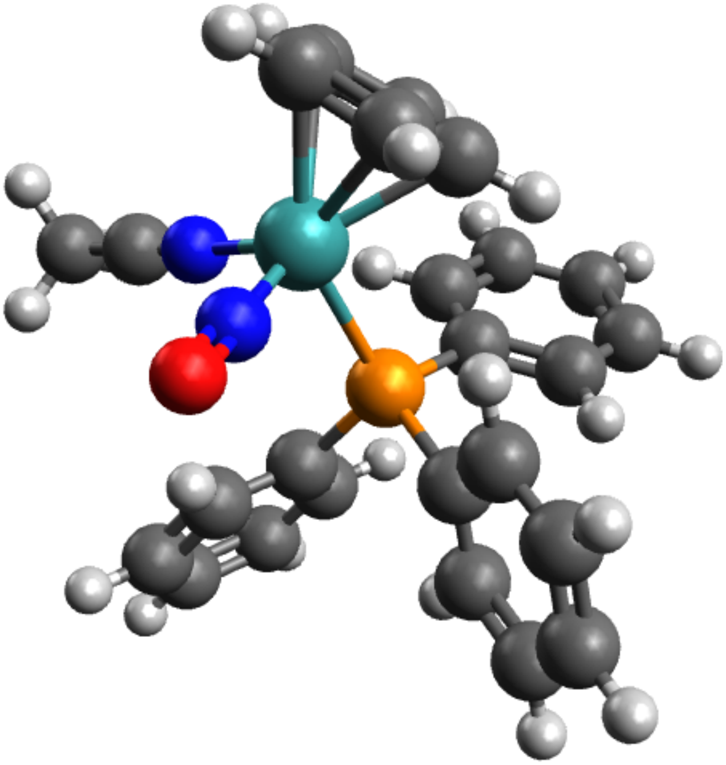

Figure S80: Optimized structure of $[Tc(NO)(Cp)(PPh_3)(NCCH_3)]^+$ in THF solution.

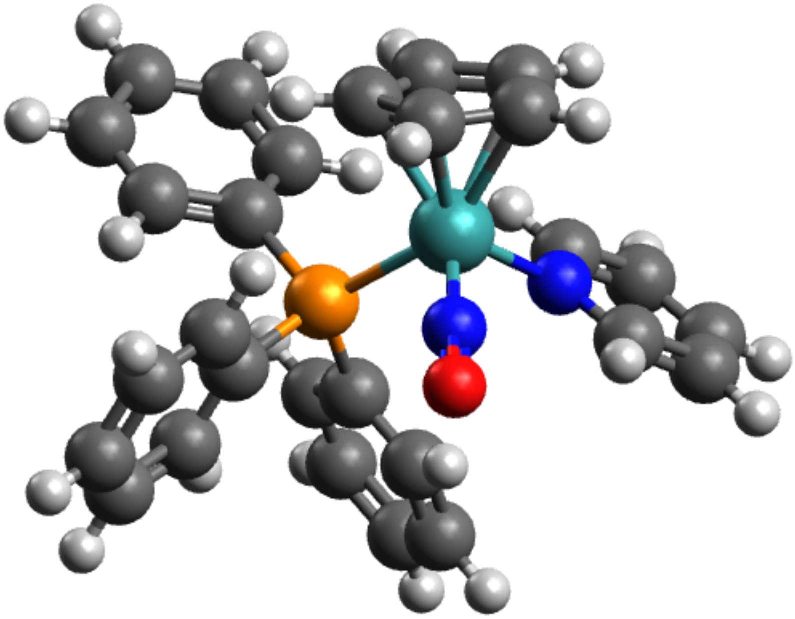

Figure S81: Optimized structure of $[Tc(NO)(Cp)(PPh_3)(pyridine)]^+$ in THF solution.

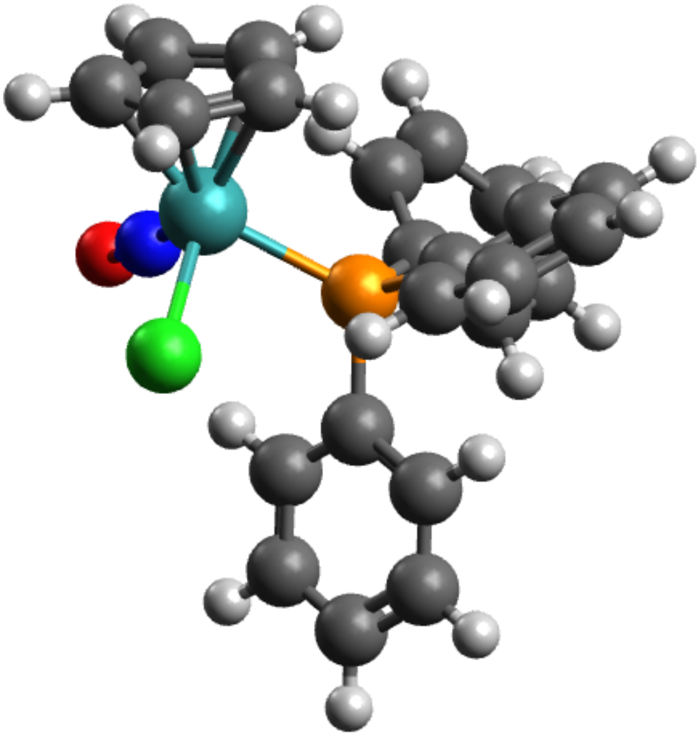

Figure S82: Optimized structure of $[Tc(NO)(Cp)(PPh_3)Cl]$ in THF solution.

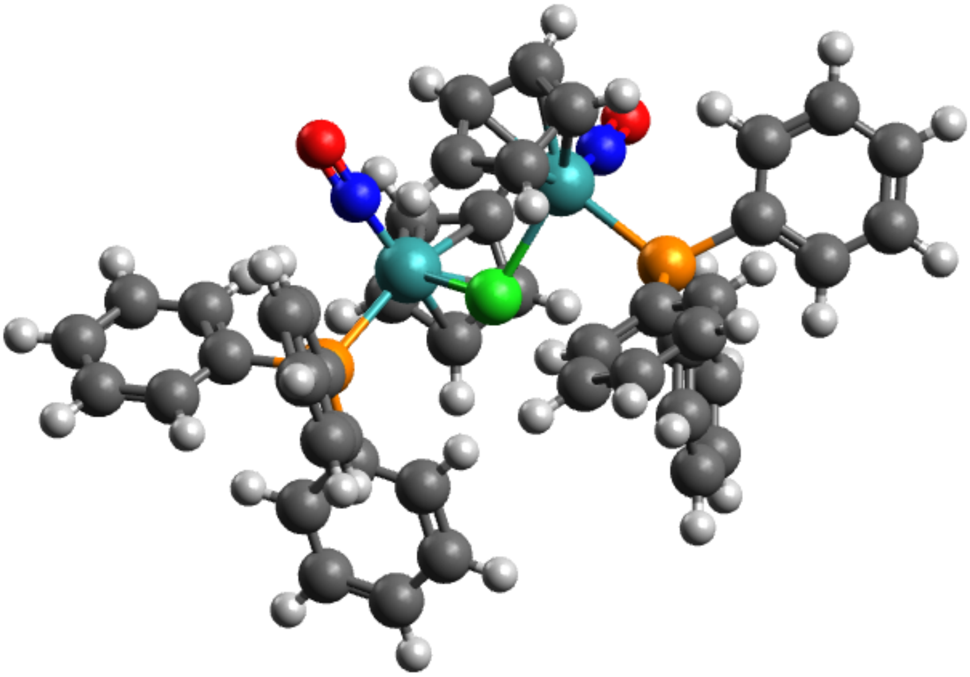

Figure S83: Optimized structure of [Tc(NO)(Cp)(PPh_3){μ-ClTc(NO)(Cp)(PPh_3)}]$^+$ in THF solution.

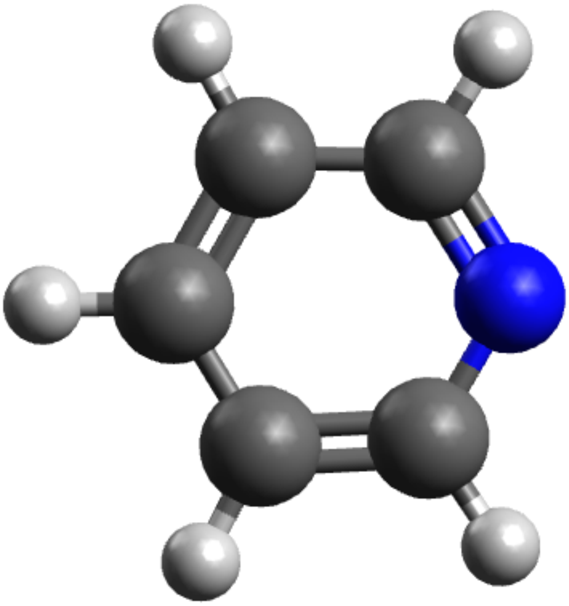

Figure S84: Optimized structure of pyridine in THF solution.

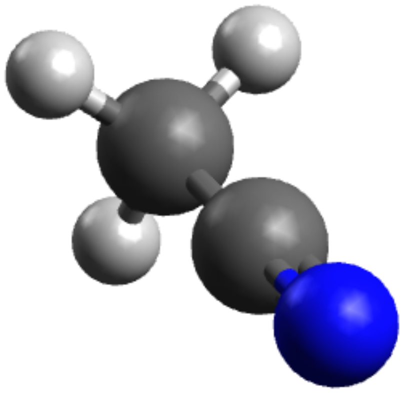

Figure S85: Optimized structure of acetonitrile in THF solution.

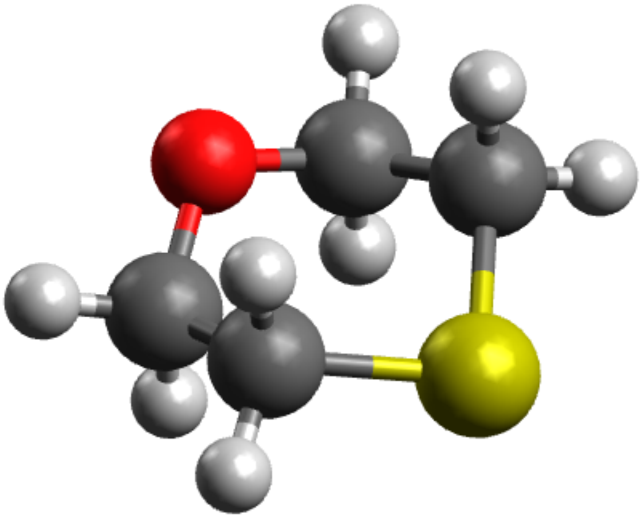

Figure S86: Optimized structure of thioxane in THF solution.

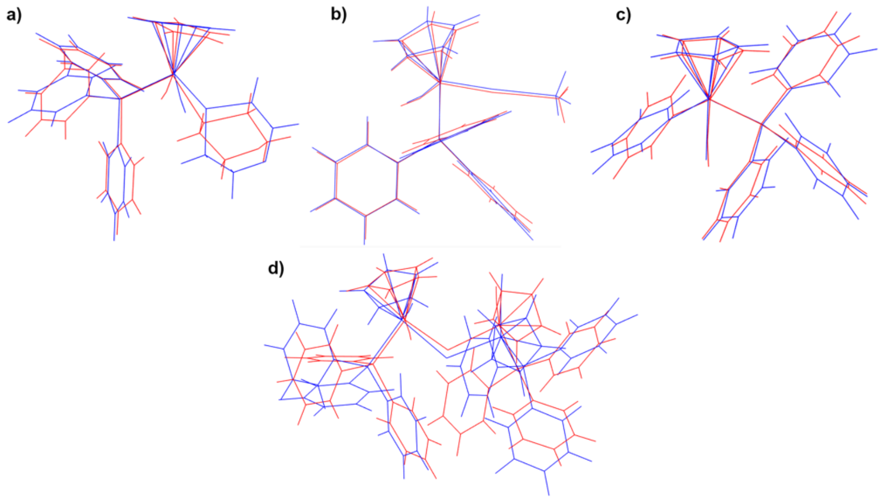

Figure S87: Overlays of the computed structures (blue) of a) $[Tc(NO)(Cp)(thioxane)]^{+}$, b) $[Tc(NO)(Cp)(NCCH_3)]^{+}$, c) $[Tc(NO)(Cp)(py)]^{+}$ and d) $[\{Tc(NO)(Cp)(PPh_3)\}_2Cl]^{+}$ in THF with the corresponding structure derived from the X-ray diffraction data (red).

Table S11: Thermochemistry of *S* versus *O* coordination in thioxane. $k = 8.314462618 \times 10^{-3}$ kJ/(mol·K); energy conversion: 1 [a.u.] = 2625.50 [kJ/mol].

	gas	solution
ΔG(*O*-thioxane) [Hartree]	-1348.03082	-1348.084856
ΔG(*S*-thioxane) [Hartree]	-1348.04602	-1348.095816
$\Delta\Delta G(\Delta G(S)-\Delta G(O))$ [Hartree]	-0.015196	-0.01096
$\Delta\Delta G$ [kJ/mol]	-39.9	-28.8
$N_O/N_S = \exp(\Delta\Delta G/kT)$; RT	7.72E-06	7.42E-04
$N_O/N_S = \exp(\Delta\Delta G/kT)$; 120°C	4.98E-04	1.50E-02

Table S12: Thermochemistry (ΔG) for the dissociation of ligands from $[Tc(NO)(Cp)(PPh_3)]^+$ with some ligands of this study in THF solution. Energy conversion: 1 [a.u.] = 2625.50 [kJ/mol].

Ligand	$[Tc(NO)(Cp)(PPh_3)]^+$ adducts	Free L	$\Sigma([Tc(NO)(Cp)(PPh_3)]^+ + L)$	$\Delta\Delta G$ [Hartree]	$\Delta\Delta G$ [kJ/mol]
None	-1105.548				
Cl^-	-1120.738	-15	-1120.666	0.072	189
CH_3CN	-1238.339	-133	-1238.308	0.032	83
Py	-1353.851	-248	-1353.824	0.027	72
thioxane_S	-1348.096	-243	-1348.087	0.009	24
**1***	-2226.295	-1121	-2226.286	0.009	23
thioxane_O	-1348.085	-243	-1348.087	-0.002	-5

* = {μ-ClTc(NO)(Cp)(PPh_3)}

Table S13: Experimental *versus* some preliminary DFT-based chemical shifts of technetium compounds.

	experiment	DFT			
	δ [ppm]	$\delta_{isotropic}$[ppm]	anisotropy	δ_{theor}	$\lvert\Delta(\delta_{exp}-\delta_{theor})\rvert$
TcO_4^-	0	-2229	0	0	
$[Tc(NO)(Cp)Cl(PPh_3)]$	**-231**	-2287	1658	**58**	289
$[Tc(NO)(Cp)(OPPh_3)(PPh_3)]^+$	**254**	-2826	2315	**598**	353
$[Tc(NO)(Cp)(SPPh_3)(PPh_3)]^+$	**-781**	-1746	919	**-483**	300
$[Tc(NO)(Cp)(SePPh_3)(PPh_3)]^+$	**-881**	-1648	763	**-581**	295

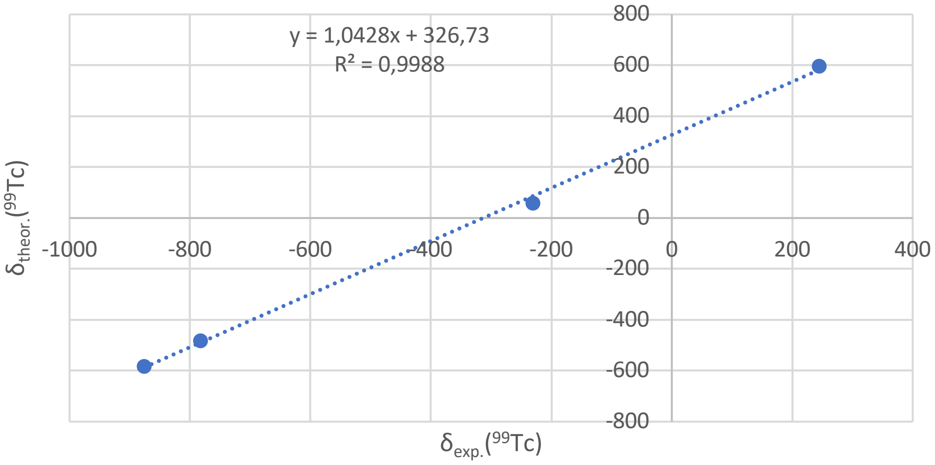

Figure S88: Linear correlation for the theoretical and experimental chemical shifts shown in Table S12. Note that while these compounds correlate fairly well, the other complexes of this study are much more difficult to model with regard to their theoretical ^{99}Tc NMR properties due to complex solvent effects or potential dynamic behavior and a dedicated manuscript for the theoretical description of ^{99}Tc NMR chemical shifts is planned for the future to address these issues in detail.

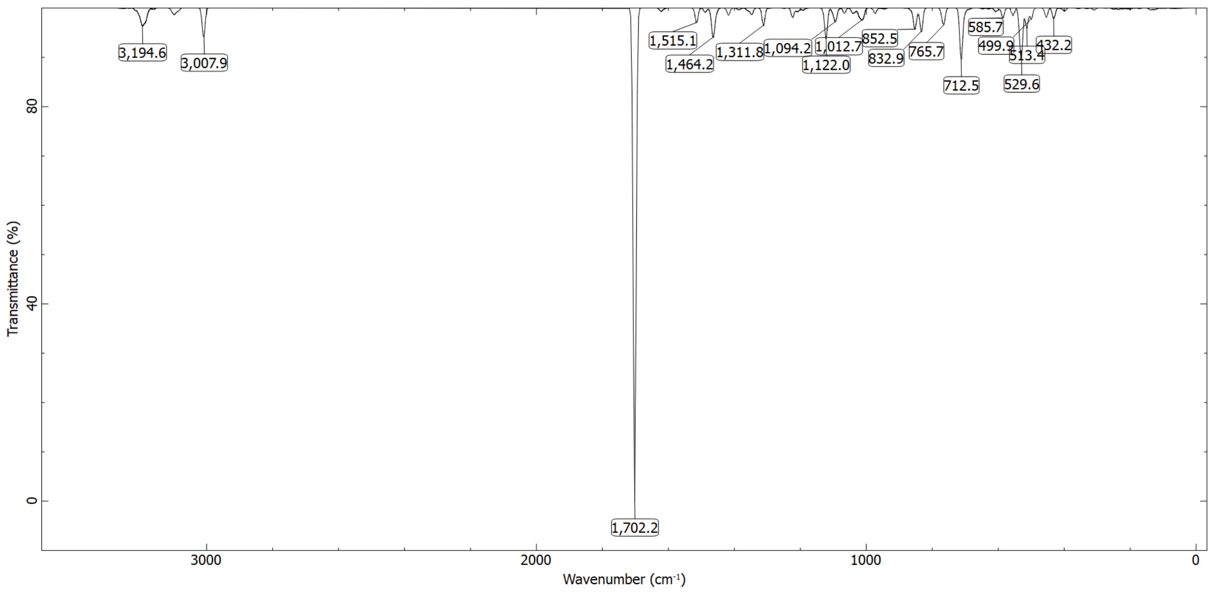

Figure S89: Theoretical IR spectrum (intensity cut-off for peak-labels: 2%) of [Tc(NO)(Cp)(PPh$_3$)(*S*-thioxane)]$^+$ in THF solution.

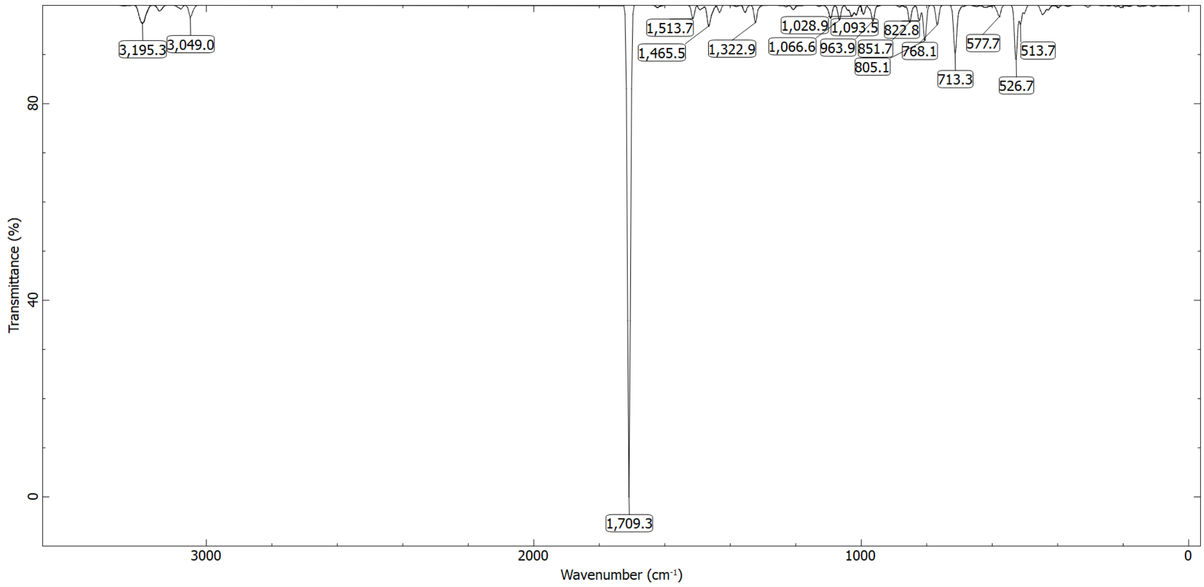

Figure S90: Theoretical IR spectrum (intensity cut-off for peak-labels: 2%) of [Tc(NO)(Cp)(PPh_3)(*O*-thioxane)]$^+$ in THF solution.

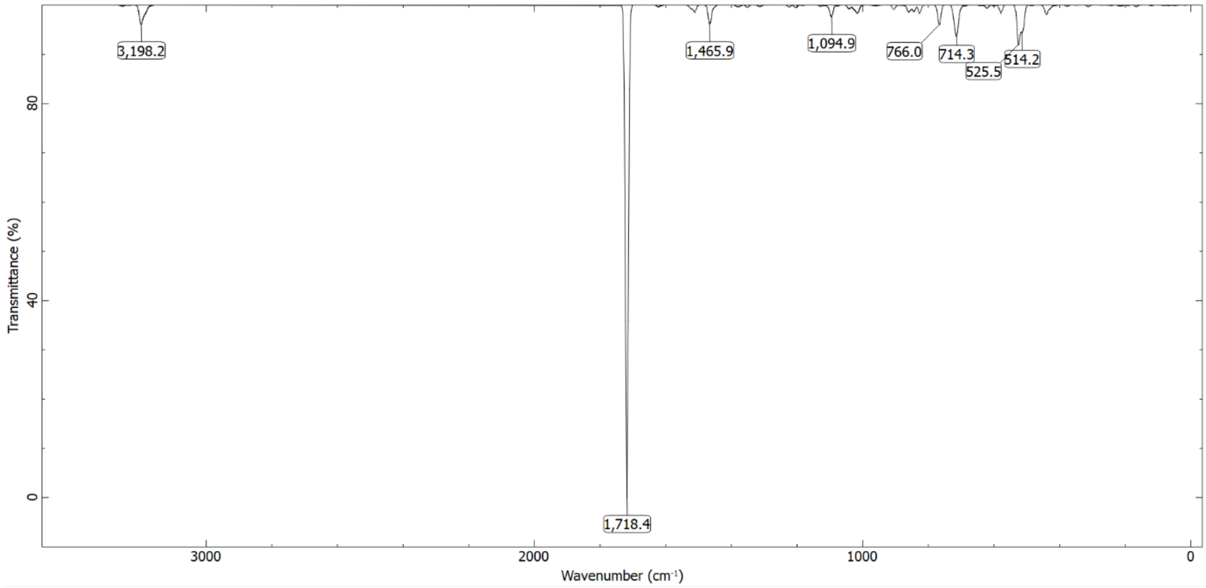

Figure S91: Theoretical IR spectrum (intensity cut-off for peak-labels: 2%) of [Tc(NO)(Cp)(PPh_3)]$^+$ in THF solution.

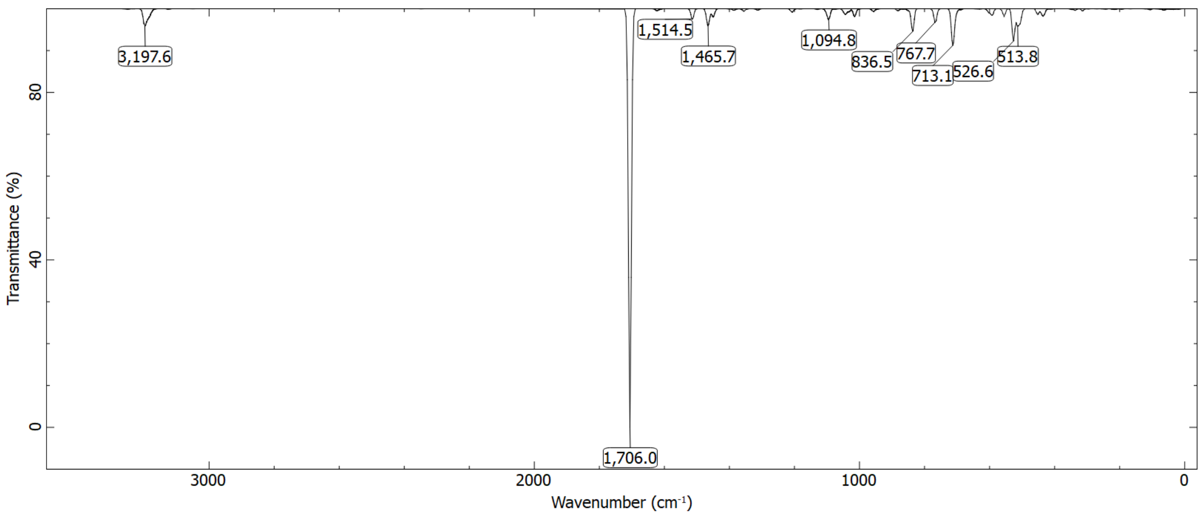

Figure S92: Theoretical IR spectrum (intensity cut-off for peak-labels: 2%) of $[Tc(NO)(Cp)(PPh_3)(NCCH_3)]^+$ in THF solution.

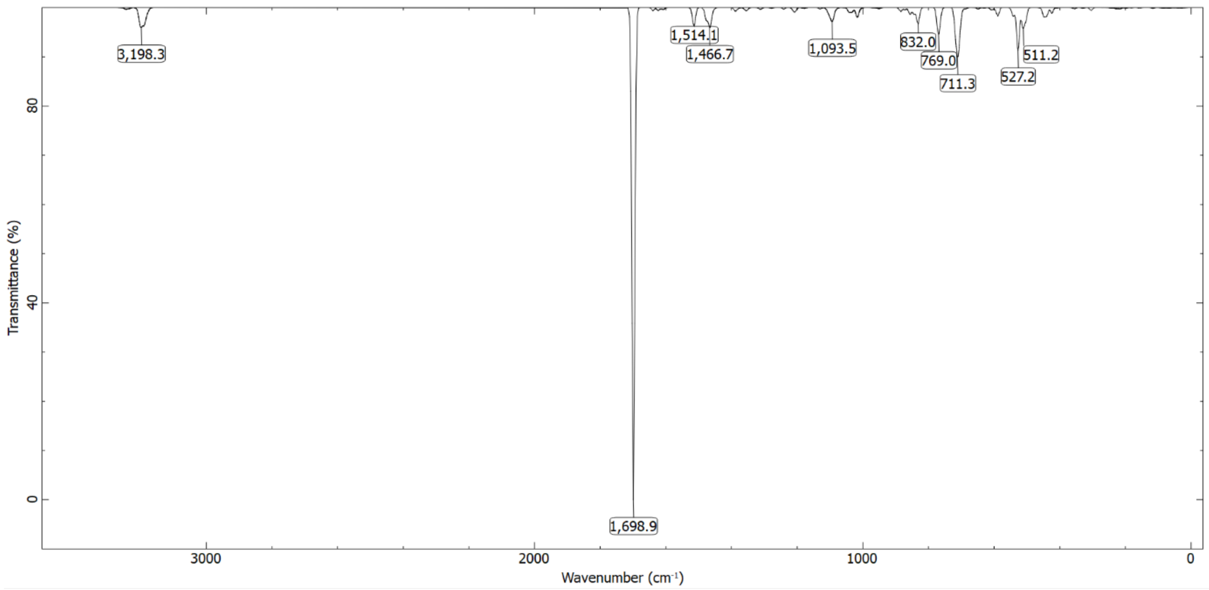

Figure S93: Theoretical IR spectrum (intensity cut-off for peak-labels: 2%) of $[Tc(NO)(Cp)(PPh_3)(pyridine)]^+$ in THF solution.

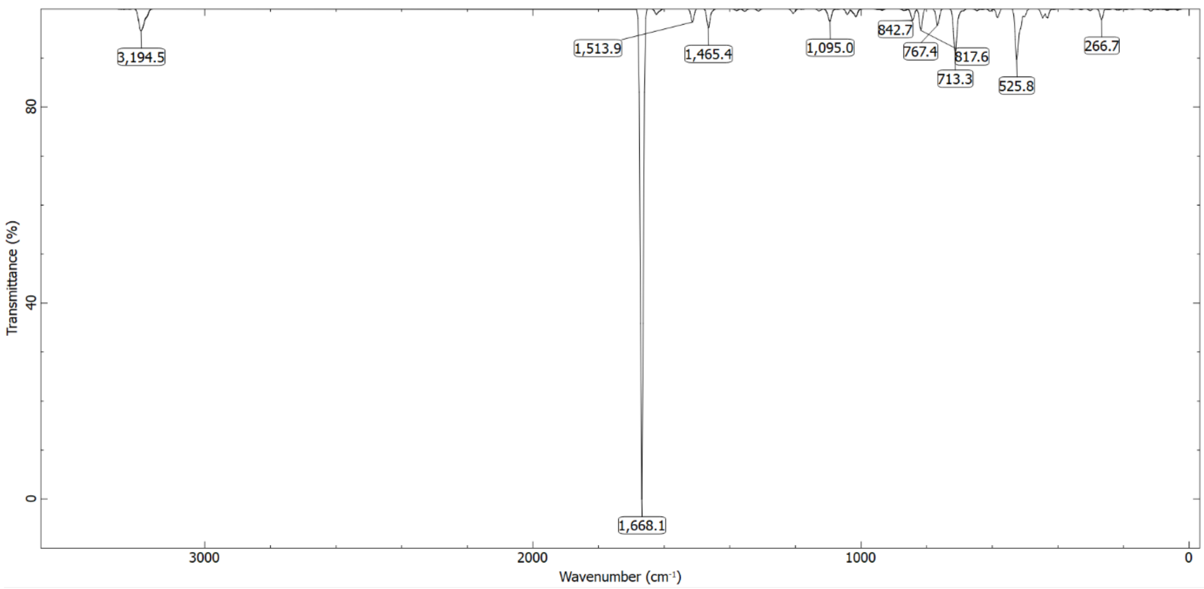

Figure S94: Theoretical IR spectrum (intensity cut-off for peak-labels: 2%) of [Tc(NO)(Cp)(PPh_3)Cl] in THF solution.

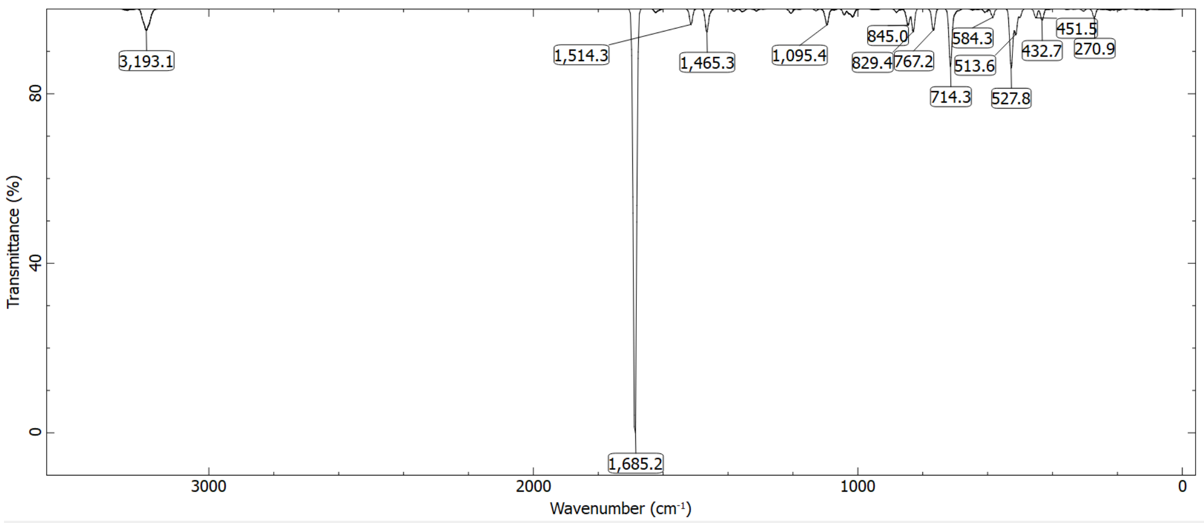

Figure S95: Theoretical IR spectrum (intensity cut-off for peak-labels: 2%) of [Tc(NO)(Cp)(PPh_3){μ-ClTc(NO)(Cp)(PPh_3)}]$^+$ in THF solution.

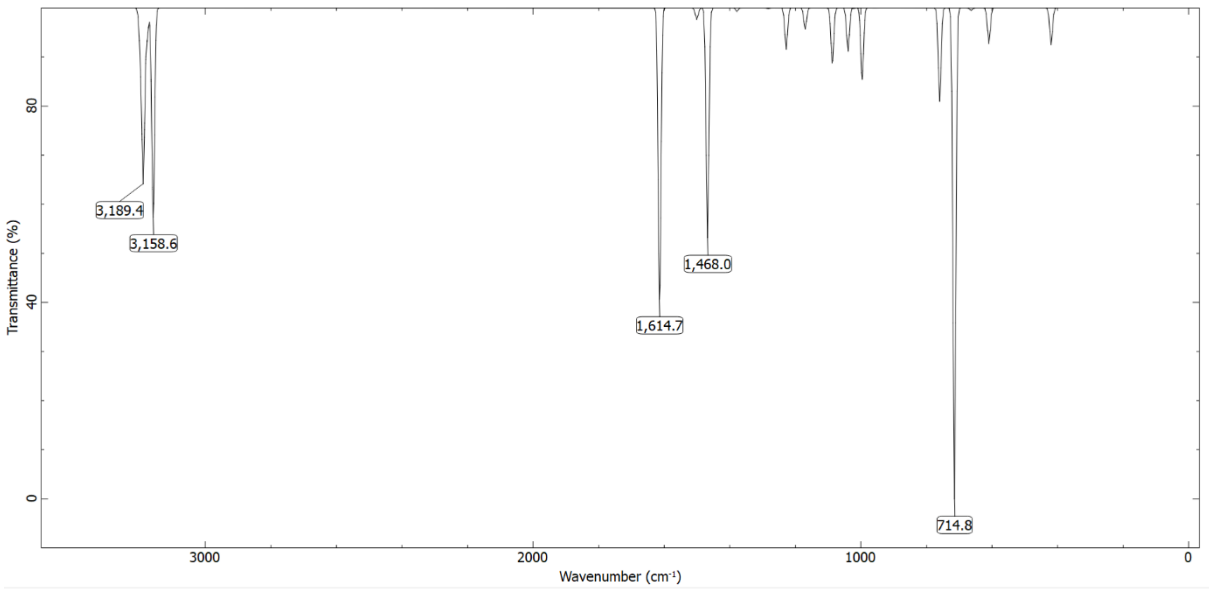

Figure S96: Theoretical IR spectrum (intensity cut-off for peak-labels: 20%) of pyridine in THF solution.

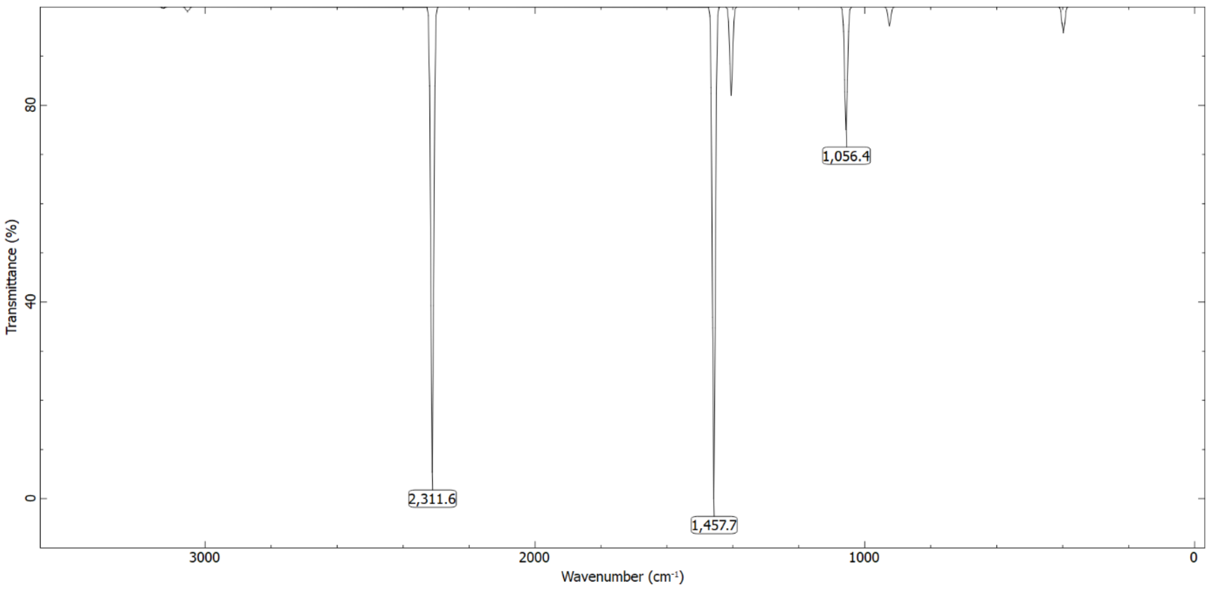

Figure S97: Theoretical IR spectrum (intensity cut-off for peak-labels: 20%) of acetonitrile in THF solution.

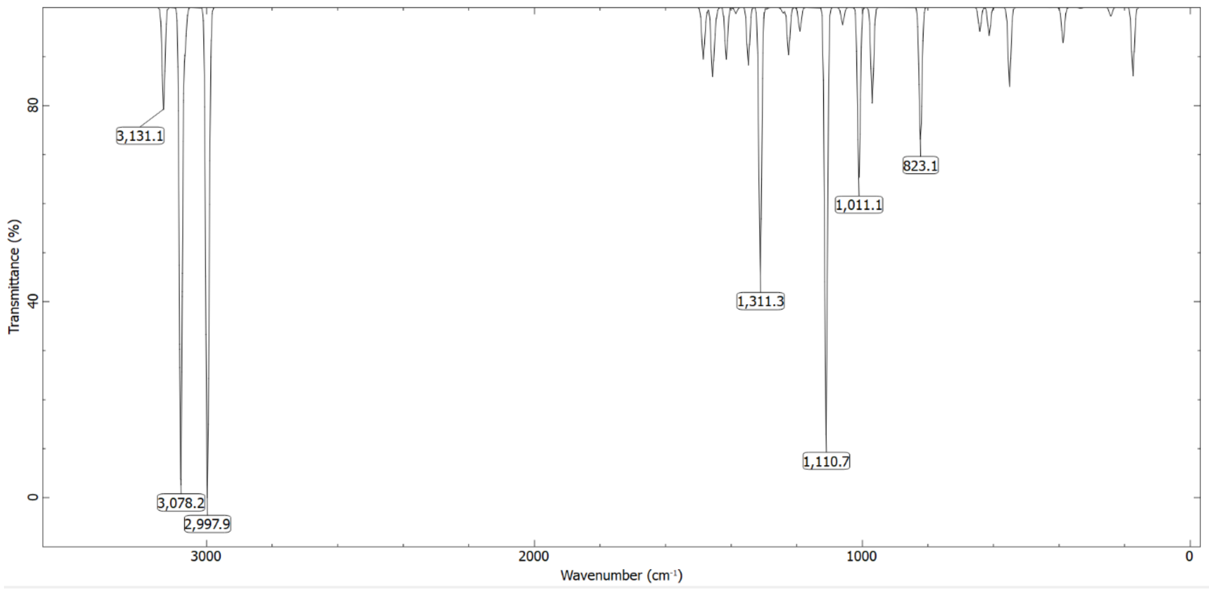

Figure S98: Theoretical IR spectrum (intensity cut-off for peak-labels: 20%) of thioxane in THF solution.
